# Genome-wide analysis of brain age identifies 59 associated loci and unveils relationships with mental and physical health

**DOI:** 10.1038/s43587-025-00962-7

**Published:** 2025-10-03

**Authors:** Philippe Jawinski, Helena Forstbach, Holger Kirsten, Frauke Beyer, Arno Villringer, A. Veronica Witte, Markus Scholz, Stephan Ripke, Sebastian Markett

**Affiliations:** 1 Department of Psychology, Humboldt-Universität zu Berlin, Berlin, Germany; 2 LIFE-Leipzig Research Center for Civilization Diseases, Leipzig University, Leipzig, Germany; 3 Institute for Medical Informatics, Statistics and Epidemiology, Leipzig University, Leipzig, Germany; 4 Cognitive Neurology, University of Leipzig Medical Center & Department of Neurology, Max Planck Institute for Human Cognitive and Brain Sciences, Leipzig, Germany; 5 Stanley Center for Psychiatric Research, Broad Institute of the Massachusetts Institute of Technology and Harvard University, Cambridge, MA USA; 6 Department of Psychiatry and Psychotherapy, Charité-Universitätsmedizin, Berlin, Germany

**Keywords:** Genome-wide association studies, Genetics of the nervous system, Ageing, Brain, Neural ageing

## Abstract

Neuroimaging and machine learning are advancing research into the mechanisms of biological aging. In this field, ‘brain age gap’ has emerged as a promising magnetic resonance imaging-based biomarker that quantifies the deviation between an individual’s biological and chronological age of the brain. Here we conducted an in-depth genomic analysis of the brain age gap and its relationships with over 1,000 health traits. Genome-wide analyses in up to 56,348 individuals unveiled a heritability of 23–29% attributable to common genetic variants and highlighted 59 associated loci (39 novel). The leading locus encompasses *MAPT*, encoding the tau protein central to Alzheimer’s disease. Genetic correlations revealed relationships with mental health, physical health, lifestyle and socioeconomic traits, including depressed mood, diabetes, alcohol intake and income. Mendelian randomization indicated a causal role of high blood pressure and type 2 diabetes in accelerated brain aging. Our study highlights key genes and pathways related to neurogenesis, immune-system-related processes and small GTPase binding, laying the foundation for further mechanistic exploration.

## Abstract (web summary)

This genomic study of magnetic resonance imaging-based brain age in 56,348 people identifies 59 genetic loci, links brain aging to mental and physical health, and suggests high blood pressure and type 2 diabetes as causal factors of brain aging.

## Main

Aging is a complex phenomenon inherent to most organisms^1–3^. As human lifespans extend and global populations age, age-related disabilities, including dementia, are rising^4^. Thus, understanding the biological mechanisms of aging is an urgent priority for social systems, to sustain longer lives with reduced periods of disability.

The use of neuroimaging methods in conjunction with machine learning has become a promising avenue in biomedical research to capture an individual’s biological age, particularly ‘brain age’^5,6^. Brain age is typically assessed by training an age prediction model on in vivo magnetic resonance imaging (MRI) data from a normative lifespan sample. This model is then applied to the MRI data of unseen individuals to predict their age. The discrepancy between an individual’s brain-predicted and chronological age is termed ‘brain age gap’ (BAG) and it is used to infer typical and atypical aging trajectories^6,7^.

A positive BAG, interpreted as accelerated aging, has been linked to reduced mental and physical health^5^; including weaker grip strength, higher blood pressure, diabetes, adverse drinking and smoking behavior, poorer cognitive abilities and depression^8–13^. BAG is also enhanced in neurological and psychiatric disorders such as Alzheimer’s disease (AD), schizophrenia and bipolar disorder^14,15^. While previous genetic studies suggested that BAG exhibits a substantial heritable component, few genetic variants have been identified^15–24^. To refine the genetic architecture of BAG and identify potential therapeutic targets for healthy aging, further research is imperative.

In this article, we present what is to our knowledge the largest genome-wide association study (GWAS) of BAG to date. We begin by discovering new loci in a sample of 32,634 individuals of White British ancestry and replicate our findings in a multi-ancestry sample of up to 23,714 individuals. Next, we conduct meta-analyses across the discovery and replication samples, with an aggregated sample size of up to 56,348 individuals. This represents a 79% increase (~25,000 more) over previous GWAS^23,24^. To prioritize genes, we use complementary fine-mapping, annotation and transcriptomic analyses that integrate multiple omics resources. We also calculate polygenic scores (PGS) to estimate the present yield in variance explanation, compute genetic correlations with over 1,000 traits and test causal effects using Mendelian randomization. Finally, we examine the degree of polygenicity of BAG and project future discovery potential. Through these efforts, we unravel new biological mechanisms behind BAG, including pathways related to neurogenesis, immune system processes and binding of small GTPases—evolutionarily conserved proteins that act as cellular timers.

## Results

We estimated brain age with a well-established and extensively validated workflow based on CAT12 voxel-based morphometry^5,25^. Using T1-weighed MRI scans and supervised machine learning, we estimated brain age through cross-prediction in a discovery sample of 32,634 individuals of White British ancestry from the UK Biobank (UKB) cohort (age range = 45–81 years)^26^. To capture tissue-specific aging patterns, we conducted separate analyses for gray matter (GM) and white matter (WM) segmentations^18^. Brain age estimation relied on an ensemble of complementary machine learning algorithms: the sparse Bayesian relevance vector machine^27^ (RVM) and extreme gradient boosting (XGBoost) with tree and linear boosters^28^. Models were stacked within and across tissue classes, yielding three brain-predicted age estimates per individual: for GM, WM and combined GM and WM.

**Fig. 1:**
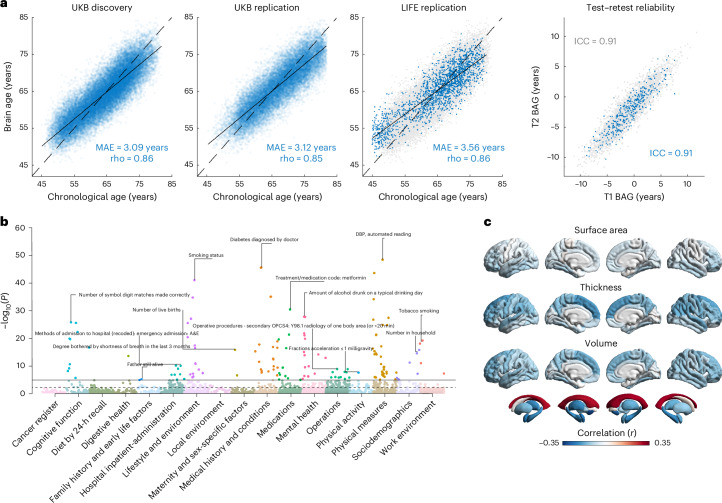
Phenotypic characteristics and associations of combined BAG. **a**, The blue dots in the first three plots (left to right) show brain-predicted age estimates (combined GM and WM) plotted against chronological age in the UKB discovery sample (*n* = 32,634), UKB replication sample (*n* = 21,881, merged across ancestries) and the LIFE-Adult replication sample (*n* = 1,833). To facilitate comparisons, the results of the UKB discovery sample are also shown as gray dots in the background of the LIFE replication plot. At this stage, brain-predicted age estimates have not yet been bias-corrected for regression dilution, as indicated by the solid linear regression line crossing the dashed identity line. The fourth plot shows the test–retest reliabilities of combined BAG in a subset of the UKB discovery (gray dots, *n* = 3,751) and UKB replication sample (blue dots, *n* = 395). BAG was residualized for sex, age, age^2^, scanner site and total intracranial volume. **b**, Cross-trait association results between combined BAG and 7,088 UKB phenotypes across several health domains. Analyses were conducted using PHESANT, which applies data-type-specific regression models (linear, logistic, ordered logistic or multinomial logistic regression). All models included sex, age, age^2^, scanner site and total intracranial volume as covariates. The horizontal lines indicate the Bonferroni-adjusted (solid) and FDR-adjusted (dashed) two-sided level of significance. The top associations per category are annotated. **c**, Surface plots showing the correlations between combined BAG and 220 FreeSurfer brain structure variables. The colors reflect the strength and direction of partial product-moment correlations (sex, age, age^2^, scanner site and total intracranial volume served as covariates). ICC, intraclass correlation coefficient (C, 1); rho, product-moment correlation coefficient.

**Extended Data Fig. 1:**
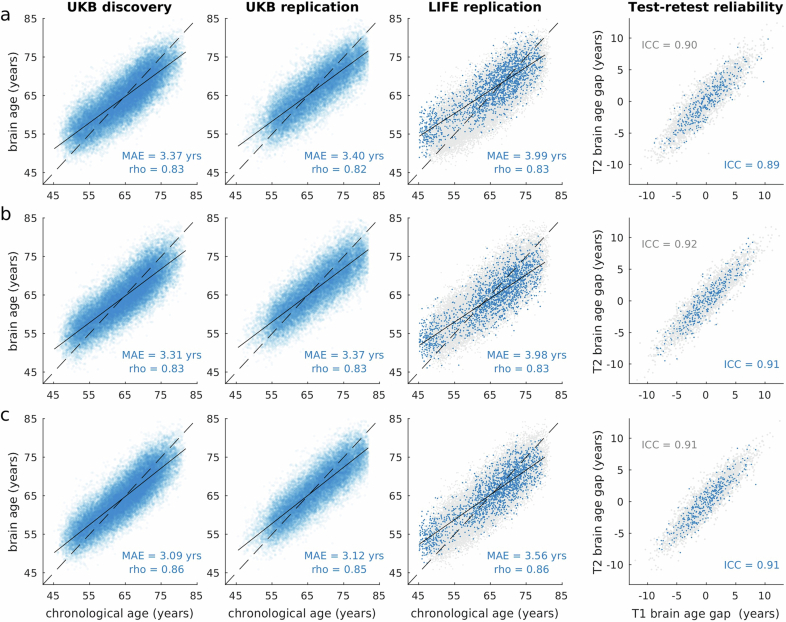
Accuracy and reliability of brain age estimation models across tissue classes and samples. (**a**) grey matter, (**b**) white matter, and (**c**) combined grey and white matter age estimation models. Blue dots in the first three plots (from left to right) show brain-predicted age estimates plotted against the chronological age in the UKB discovery sample (*n* = 32,634; white-British ancestry), UKB replication sample (*n* = 21,881; multi-ancestry), and LIFE-Adult replication sample (*n* = 1,833; European ancestry). To facilitate comparisons, results of the UKB discovery sample are also shown as grey dots in the background of the LIFE replication plots. At this stage, brain-predicted age estimates have not yet been bias-corrected for regression dilution, that is, younger participants’ ages are systematically overestimated and vice versa, as indicated by the linear regression line (solid) crossing the identity line (dashed). The fourth plot shows the test-retest reliabilities of brain age gap (i.e., the difference between brain-predicted and chronological age) in a subset of the UKB discovery (grey dots, *n* = 3,751) and UKB replication sample (blue dots, *n* = 395). For test-retest comparisons, brain age gap was residualized for effects of age, age^2^, sex, scanner site, and total intracranial volume. MAE: mean absolute error; rho: product-moment correlation coefficient. ICC: intraclass correlation coefficient (C, 1).

In the discovery sample, we observed accurate predictions for chronological age, with mean absolute errors (MAEs) reaching 3.09 years and correlation coefficients attaining *r* = 0.86 (Fig. 1a, Table 1, Supplementary Table 1 and Extended Data Fig. 1). Model performances were similar in the multi-ancestry UKB replication samples (*n* = 21,881; age range = 45-81 years) and the European-ancestry Leipzig Research Centre for Civilization Diseases (LIFE)-Adult replication sample (*n* = 1,833; age range = 45-80 years)^29,30^. Genetic association analyses were performed on BAG, that is, the difference between brain-predicted and chronological age. These BAG estimates—residualized for sex, age, age^2^, scanner site and total intracranial volume—showed high test–retest reliabilities, with intraclass correlation coefficients (ICCs) ranging from 0.89 to 0.92.

**Table 1:** Prediction accuracies of the stacked age estimation models stratified according to tissue class

	UKB discovery (*n* = 32,634) 45–81 years				UKB replication (*n* = 21,881) 45–81 years				LIFE replication (*n* = 1,833) 45–80 years		
	*r*	*R*^2^	MAE	ICC_BAG_	*r*	*R*^2^	MAE	ICC_BAG_	*r*	*R*^2^	MAE
GM	0.827	0.683	3.372	0.899	0.825	0.676	3.405	0.888	0.828	0.653	3.990
WM	0.835	0.696	3.307	0.920	0.830	0.683	3.368	0.911	0.829	0.667	3.979
GM and WM	0.857	0.734	3.089	0.915	0.854	0.726	3.123	0.908	0.862	0.729	3.557

The imaging data of the UKB discovery sample were released until January 2020 (release 1.7), whereas the data of the UKB replication sample were released until May 2024 (release 1.10). *r* indicates the product-moment correlation between brain-predicted age (without bias correction) and chronological age. *R*^2^ indicates the coefficient of determination (not equivalent to *r*^2^). ICC_BAG_ indicates the ICC between the test and retest assessment of BAG. MAE is the MAE of brain-predicted versus chronological age. BAG was bias-corrected for age, age^2^, sex, scanner site and total intracranial volume. ICCs are based on a subset of 3,751 individuals in the UKB discovery sample and 395 individuals in the UKB replication sample.

### Phenotypic associations

**Extended Data Fig. 2:**
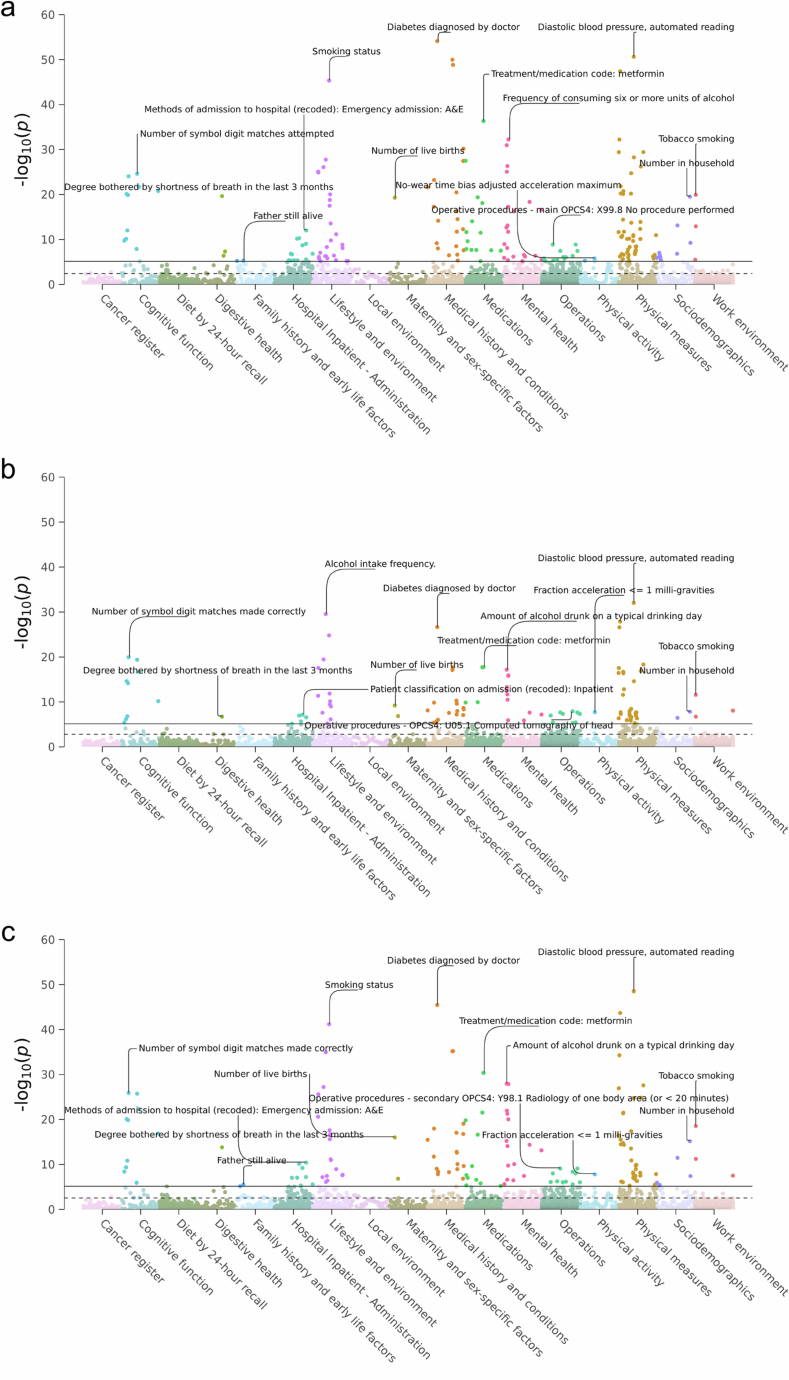
Cross-trait associations between brain age gap and other UK Biobank phenotypes. Each dot represents an association between brain age gap and one of 7,088 non-imaging derived phenotypes in the UKB discovery sample (*n* = 32,634). *P* values (-log10 scale) are shown on the y-axis, with phenotypes on the x-axis categorized by their UK Biobank data dictionary path. Analyses were conducted using PHESANT, which applies data-type-specific regression models (linear, logistic, ordered logistic, or multinomial logistic regression). All models included sex, age, age^2^, scanner site, and total intracranial volume as covariates. Horizontal lines indicate the Bonferroni-adjusted (solid) and FDR-adjusted (dashed) level of significance (two-sided). The top associations in each category are annotated. (**a**) GM BAG, (**b**) WM BAG, (**c**) combined BAG.

To validate our BAG estimates and extend previous evidence on their health relevance^31,32^, we conducted cross-trait association analyses between BAG and 7,088 non-imaging-derived phenotypes using PHESANT^33^. A total of 210 associations reached Bonferroni significance (*P* < 7.1 × 10^−6^) for at least 1 of the 3 BAG traits (Supplementary Table 2 and Extended Data Fig. 2). Figure 1b presents the cross-trait results for combined GM and WM BAG.

Top associations for combined BAG (all *P* ≤ 1.8 × 10^−12^) included pack years of smoking (*r* = 0.091), diastolic blood pressure (DBP) (*r* = 0.084), number of symbol digit matches made correctly (that is, a measure of cognitive performance; *r* = −0.082), diabetes diagnosed by doctor (*r* = 0.079), amount of alcohol drunk on a typical drinking day (*r* = 0.076) and overall health rating (*r* = 0.039; note that higher scores indicate poorer health). These results corroborate earlier BAG associations^31^ and expand known health-related links.

To examine regional contributions to BAG and facilitate comparisons with prior surface-based morphometry studies^15,19,34^, we analyzed associations with FreeSurfer-derived^35^ cortical surface measures and subcortical volumes (Fig. 1c, Supplementary Fig. 3 and Supplementary Table 3). For combined BAG, the strongest associations (all *P* ≤ 1.1 × 10^−209^) were observed with the volumes of the accumbens (*r* = −0.31), lateral ventricles (*r* = 0.30), amygdala (*r* = −0.25), hippocampus (*r* = −0.23) and thalamus (*r* = −0.22), and the cortical thickness of the superior frontal (*r* = −0.20) and inferior parietal (*r* = −0.17) cortex. These findings suggest that our models capture patterns of aging distributed throughout the brain, rather than being confined to specific areas.

Following up on Smith et al.^31^, we also performed sex-stratified analyses, which revealed largely similar BAG associations in males and females for non-imaging-derived (Supplementary Figs. 1 and 2 and Supplementary Table 4) and brain structure phenotypes (Supplementary Figs. 4 and 5 and Supplementary Table 5). Some differences emerged, for example, stronger associations between GM BAG and body fat percentage in males, but were generally small in magnitude.

### Concordant genomic signals in discovery and replication GWAS

**Fig. 2:**
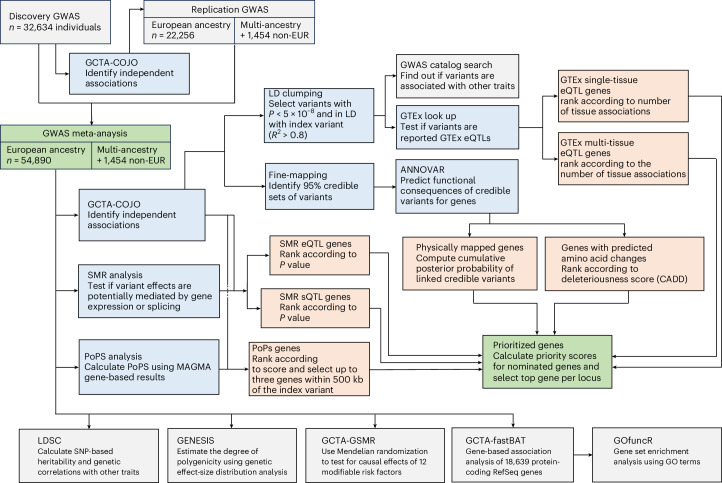
Genomic analysis workflow and gene prioritization strategy. Overview of the genomic analysis workflow with particular emphasis on the gene prioritization procedure. The green boxes represent data input (GWAS meta-analysis) and output (prioritized genes). The blue boxes represent analyses whose outcomes were used for gene nomination and subsequent prioritization. The apricot-colored boxes reflect the gene nomination categories. The gray boxes reflect all other analyses carried out to refine the genetic architecture of BAG, such as heritability and polygenicity analyses. Genes were prioritized by integrating data from multiple strategies, such as functional annotation of credible variants, SMR, GTEx eQTL lookup and PoPS.

An overview of our genomic analysis workflow is shown in Fig. 2. To examine the reliability and replicability of our findings, we first compared results from a discovery GWAS of 32,634 individuals of White British ancestry (UKB imaging release v.1.7) with a replication GWAS of 22,256 individuals of European ancestry (UKB imaging release v.1.10 and LIFE-Adult).

The discovery and replication GWAS results were highly consistent, with strong genetic correlations derived from bivariate linkage disequilibrium (LD) score regression (LDSC) (all *r*_g_ > 0.996, all *P* > 2.9 × 10^−36^; Supplementary Table 6)^36^. We discovered 25 independent genome-wide significant loci across the 3 BAG traits, all showing concordant effect directions (binomial test: *P* = 3.0 × 10^−08^), and 18 reaching one-tailed nominal significance in replication (binomial test: *P* = 1.3 × 10^−18^; Supplementary Table 7). These findings closely align with our power analysis, which predicted 19 nominal replications. Among 45 additional suggestive discoveries (*P* < 1.0 × 10^−6^), 36 showed concordant directions (binomial test: *P* = 3.3 × 10^−5^) and 24 reached nominal significance in replication (binomial test: *P* = 8.0 × 10^−20^). Incorporating 1,458 non-European UKB participants into an extended multi-ancestry GWAS also yielded above-chance consistency (Supplementary Table 8). Together, these findings strongly support locus replicability, reinforcing the robustness of our results. Additional details are provided in Supplementary Figs. 6–23.

### Identification of 59 associated loci

**Fig. 3:**
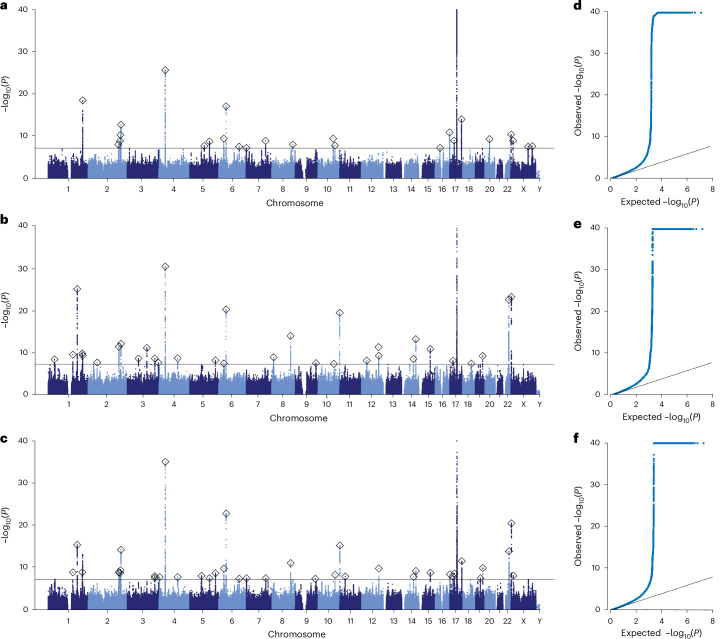
Genome-wide association meta-analyses of BAG traits. Manhattan (**a**–**c**) and quantile–quantile (QQ) plots (**d**–**f**) showing the results of the European-ancestry GWAS meta-analyses for the three BAG traits (*n* = 54,890). The Manhattan plots show the *P* values (−log_10_ scale) of the tested genetic variants on the *y* axis and base-pair positions along the chromosomes on the *x* axis. *P* values were derived from two-sided linear regression models using PLINK, followed by meta-analysis using inverse-variance weighting in METAL. The solid horizontal line indicates the threshold of genome-wide significance (two-sided *P* = 5.0 × 10^−8^, accounting for multiple testing). Index variants are highlighted by the diamonds. The results of the pseudoautosomal variants have been added to chromosome X. Quantile–quantile plots show the observed *P* values from the association analysis versus the expected *P* values under the null hypothesis of no effect (−log_10_ scale). For illustrative reasons, the *y* axis has been truncated at *P* = 1.0 × 10^−40^. **a**,**d**, GM BAG (Manhattan and QQ). **b**,**e**, WM BAG (Manhattan and QQ). **c**,**f**, Combined GM and WM BAG (Manhattan and QQ).

**Extended Data Fig. 3:**
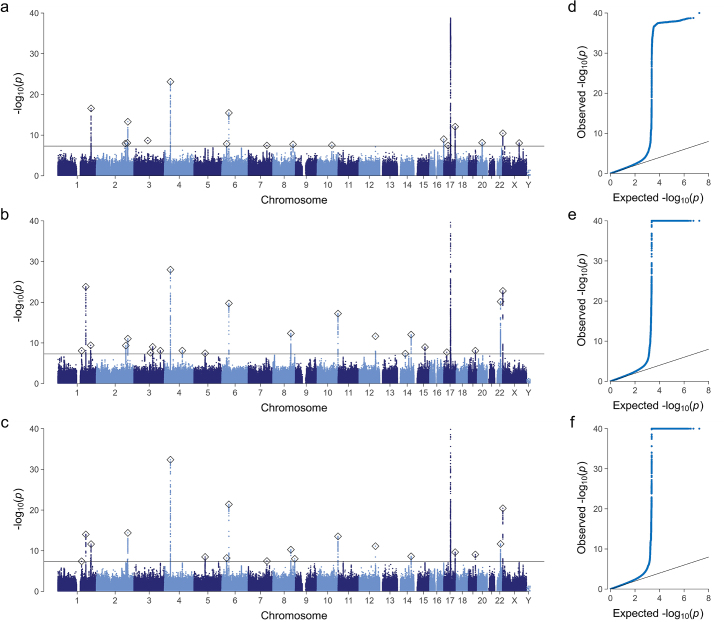
Results of the multi-ancestry GWAS meta-regression for brain age gap. Manhattan plots (**a-c**) and quantile-quantile (QQ) plots (**d-f**) show the results of the multi-ancestry meta-regression (MR-MEGA) for the three brain age gap traits (up to *N* = 56,348). Multi-ancestry analyses combine results from the discovery sample (*n* = 32,634) and up to seven replication samples: UKB African ancestry (*n* = 337), UKB Admixed American ancestry (*n* = 94); UKB Central/South Asian ancestry (*n* = 638), UKB East Asian ancestry (*n* = 291), UKB European ancestry (*n* = 20,423), UKB Middle Eastern ancestry (*n* = 98), and LIFE-Adult (European ancestry; *n* = 1,833). Manhattan plots show the *P* values (-log_10_ scale) of the tested genetic variants on the y-axis and base-pair positions along the chromosomes on the x-axis. *P* values were derived from two-sided linear regression models using PLINK, followed by meta-regression using MR-MEGA. The solid horizontal line indicates the threshold of genome-wide significance (*p* = 5.0e-08, two-sided, accounting for multiple testing). Pseudoautosomal variations have been added to chromosome ‘X’. Index variants are highlighted by diamonds and were identified by positional clumping with 3,000 kb window-size (no LD threshold was applied in multi-ancestry analyses). Quantile-quantile plots show the observed *P* values from the association analysis vs. the expected *P* values under the null hypothesis of no effect (-log_10_ scale). For illustrative reasons, the y-axis has been truncated at *p* = 1.0e-40. **a,d** grey matter brain age gap (Manhattan and QQ); **b,e** white matter brain age gap (Manhattan and QQ); **c,f** combined brain age gap (Manhattan and QQ).

To maximize statistical power and improve genetic discovery, we meta-analyzed GWAS data from 54,890 individuals of European ancestry, combining the UKB discovery (*n* = 32,634), UKB-EUR replication (*n* = 20,423) and LIFE-Adult (*n* = 1,833) cohorts. Analyses included 9.6 million single-nucleotide polymorphisms (SNPs) and insertions and deletions (indels) with a minor allele frequency (MAF) greater than 0.01 and imputation quality score (INFO) greater than 0.80. We modeled additive genetic effects with covariates for sex, age, age^2^, total intracranial volume, scanner site and type of genotyping array, and up to 20 genetic principal components. Results for the three BAG traits are shown in Fig. 3 (multi-ancestry results are shown in Extended Data Fig. 3).

LDSC intercepts did not indicate a bias of test statistics due to reasons other than polygenicity, suggesting no confounding inflation caused by population stratification (intercept range: 1.011–1.017; Supplementary Table 9)^37^. SNP-based heritability estimates ranged from 22.7% (GM BAG) to 29.0% (WM BAG). The genetic correlation between GM and WM BAG was *r*_g_ = 0.74 (s.e. = 0.023), indicating both shared and distinct genetic influences (phenotypic correlation: *r*_P_ = 0.62, s.e. = 0.003). Combined BAG showed strong genetic correlations with both GM (*r*_g_ = 0.91, s.e. = 0.008; cf. *r*_P_ = 0.88, s.e. = 0.002) and WM (*r*_g_ = 0.951, s.e. = 0.006; cf. *r*_P_ = 0.91, s.e. = 0.002) BAG. Strong genetic correlations were observed between sex-stratified results (all *r*_g_ ≥ 0.934; Supplementary Table 10), indicating highly concordant architectures across sexes.

**Extended Data Fig. 4:**
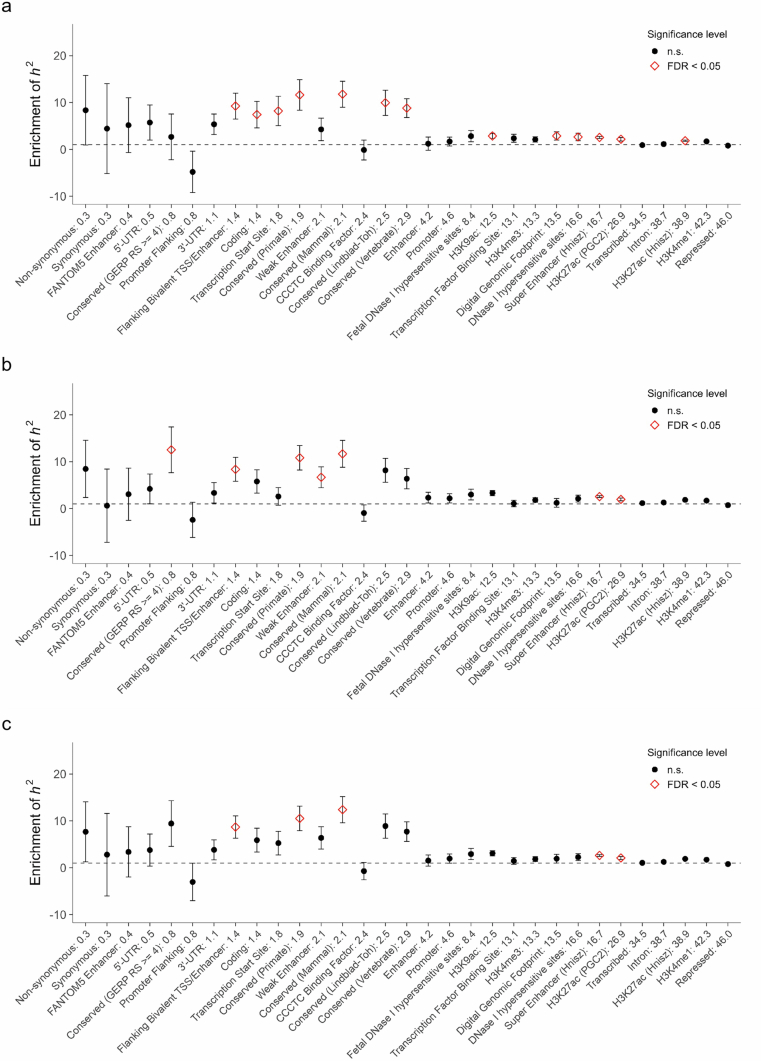
Functional partitioning of heritability for brain age gap. Enrichment of heritability (*h*^2^) across functional genomic categories for (**a**) grey matter brain age gap, (**b**) white matter brain age gap, (**c**) combined brain age gap. Results are based on GWAS meta-analyses in individuals of European ancestry (*n* = 54,890). Heritability enrichment is defined as the proportion of *h*^2^ attributable to a given category divided by the proportion of SNPs in that category. Error bars represent jackknife standard errors around the estimates of enrichment. Please note that significance is tested based on the covariance matrix for the tau coefficient estimates (adjusted for all other baseline categories), which may differ from interpretations based solely on jackknife SEs. Functional categories are ordered by the proportion of SNPs they contain (x-axis), with percentages shown at tick marks. Categories with significant enrichment are marked by red diamonds.

**Extended Data Fig. 5:**
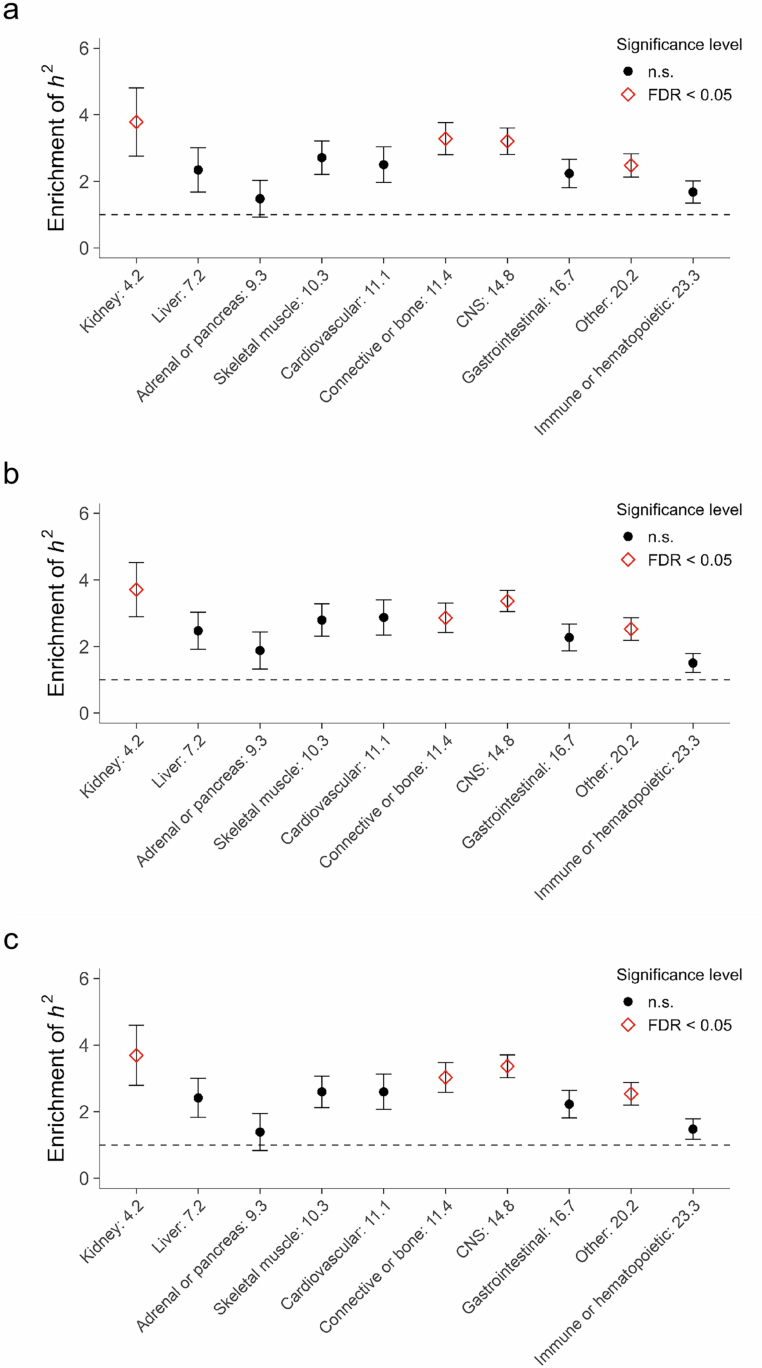
Heritability enrichment in genes specifically expressed in selected tissue groups. SNP-based heritability (*h*^2^) enrichment across genomic regions near genes with specific expression in various tissue types, for (**a**) grey matter brain age gap, (**b**) white matter brain age gap, (**c**) combined brain age gap. Results are based on GWAS meta-analyses in individuals of European ancestry (*n* = 54,890). Heritability enrichment is defined as the proportion of *h*^2^ attributable to a given category divided by the proportion of SNPs in that category. Error bars represent jackknife standard errors around the estimates of enrichment. Please note that significance is tested based on the covariance matrix for the tau coefficient estimates (adjusted for all other baseline categories), which may differ from interpretations based solely on jackknife SEs. Functional categories are ordered by the proportion of SNPs they contain (x-axis), with percentages shown at tick marks. Categories with significant enrichment are marked by red diamonds.

Partitioned LDSC^38^ (Supplementary Table 11 and Extended Data Fig. 4) revealed an enrichment of heritability (false discovery rate (FDR) < 0.05) in regions conserved across mammals (fold enrichment (FE) = 12.4) and primates (FE = 10.5). Additional enrichment was observed in super-enhancer (FE = 2.6), flanking bivalent transcriptional start sites/enhancers (FE = 8.7) and epigenetically modified H3K27ac (FE = 1.8) and H3K9ac (FE = 2.9) regions. Cell-type group analyses (Supplementary Table 12 and Extended Data Fig. 5) revealed enrichment near genes expressed in central nervous system (FE = 3.4), connective or bone (FE = 3.0) and kidney (FE = 3.7) tissue.

To identify independent genome-wide significant associations, we conducted stepwise conditional analyses using genome-wide complex trait analysis–conditional and joint association analysis (GCTA–COJO)^39^. This resulted in 26, 34 and 39 independent discoveries for GM, WM and combined BAG, respectively (regional plots are shown in Supplementary Figs. 25–42). After cross-trait LD clumping of index variants (*r*^2^ > 0.1, 10,000-kb window), we identified 59 distinct loci (≥460 kb apart; Table 2 and Supplementary Table 13). Of these, 39 represent novel discoveries not previously reported in BAG GWAS (see Methods for the definition of novelty)^17–24^.

**Table 2:** Identification of 59 genomic loci associated with BAG in *n* = 54,890 individuals

Chr	Position	ID	A1/A2	Frequency of A1	Beta (s.e.)	*P*	Credible	Prioritized gene	Phenotype(s)	Ref.
1	39471759	1:39471759	CT/C	0.89	0.20 (0.03)	4 × 10^−9^	3	*NDUFS5*	WM	*
1	153888217	rs552429854	T/TA	0.37	0.14 (0.02)	3 × 10^−^^10^	6	*DENND4B*	WM, GM and WM	^18^
1	180956936	rs35306826	A/T	0.59	−0.22 (0.02)	1 × 10^−25^	6	*STX6*	WM, GM and WM	^18,21^
1	212557599	rs3767867	T/C	0.62	−0.14 (0.02)	2 × 10^−10^	–	*PACC1*	WM, GM and WM	*
1	215139887	rs1452628	A/T	0.62	0.19 (0.02)	3 × 10^−19^	7	*KCNK2*	GM, WM	^17,23^
2	56116193	2:56116193	T/TTG	0.10	0.20 (0.04)	2 × 10^−8^	–	*EFEMP1*	WM	*
2	188028317	rs62172472	A/G	0.20	−0.15 (0.03)	7 × 10^−9^	5	*TFPI*	GM	*
2	190694175	rs1233297	T/C	0.26	0.16 (0.02)	4 × 10^−12^	32	*ORMDL1*	WM, GM and WM	^18^
2	198567638	rs12619333	C/G	0.67	0.14 (0.02)	1 × 10^−9^	17	*SF3B1*	GM, GM and WM	*
2	201160771	rs1367858	T/C	0.34	0.15 (0.02)	4 × 10^−11^	8	*SPATS2L*	GM, GM and WM	*
2	203877365	rs530464314	CA/C	0.14	−0.23 (0.03)	5 × 10^−15^	6	*CARF*	GM and WM, GM, WM	^23^
3	69893971	rs62252239	T/G	0.21	−0.15 (0.03)	2 × 10^−9^	11	*MITF*	WM	*
3	121628658	3:121628658	A/AT	0.48	−0.14 (0.02)	7 × 10^−12^	14	*SLC15A2*	WM	*
3	171040852	rs776970253	CTC/C	0.74	−0.14 (0.02)	2 × 10^−9^	7	*TNIK*	WM, GM and WM	*
3	171500441	rs72622537	A/C	0.35	−0.12 (0.02)	2 × 10^−8^	15	*PLD1*	GM and WM	*
3	193549408	rs1146045	T/C	0.59	−0.12 (0.02)	2 × 10^−8^	12	*LINC02038*	WM	*
4	2944571	rs66571798	CGT/C	0.41	−0.12 (0.02)	1 × 10^−8^	12	*GRK4*	GM and WM	*
4	38680015	rs13132853	A/G	0.63	−0.26 (0.02)	9 × 10^−36^	1	*KLF3-AS1*	GM and WM, WM, GM	^18–22,24^
4	115534729	rs75563007	T/C	0.97	−0.40 (0.07)	2 × 10^−9^	8	*UGT8*	WM, GM and WM	*
5	72257180	rs2548331	T/G	0.48	0.12 (0.02)	8 × 10^−9^	18	*FCHO2*	GM and WM	*
5	90567689	5:90567689	T/TTA	0.93	−0.24 (0.04)	2 × 10^−8^	12	*LUCAT1*	GM	*
5	122879901	rs36048468	T/C	0.21	0.16 (0.03)	1 × 10^−9^	15	*CSNK1G3*	GM, GM and WM	*
5	159528663	rs55790564	A/AT	0.40	−0.12 (0.02)	2 × 10^−9^	12	*PWWP2A*	GM and WM, WM	*
6	31249217	rs2253491	A/G	0.21	−0.16 (0.02)	1 × 10^−10^	–	*CLIC1*	GM and WM, GM, WM	^18^
6	45410312	rs910586	T/C	0.36	−0.21 (0.02)	1 × 10^−23^	2	*RUNX2*	GM and WM, WM, GM	^17–20,22,24^
6	126690257	6:126690257	A/AT	0.44	0.12 (0.02)	2 × 10^−8^	10	*CENPW*	GM, GM and WM	^17^
7	1213127	rs1543985	A/G	0.36	0.12 (0.02)	3 × 10^−8^	17	*ZFAND2A-DT*	GM and WM, GM	^24^
7	120803286	rs35789132	A/G	0.65	−0.13 (0.02)	1 × 10^−9^	11	*CPED1*	GM, GM and WM	*
8	10813904	rs10096381	T/G	0.52	0.13 (0.02)	1 × 10^−9^	–	*XKR6*	WM	^18^
8	116635942	rs2721939	T/C	0.60	0.16 (0.02)	1 × 10^−14^	4	*TRPS1*	WM, GM and WM	*
8	130903153	rs12548781	A/T	0.79	0.15 (0.03)	7 × 10^−9^	12	*FAM49B*	GM	*
9	123543953	rs5021405	T/C	0.47	0.12 (0.02)	4 × 10^−8^	–	*PHF19*	GM and WM	*
9	128010901	rs755594165	CA/C	0.61	−0.12 (0.02)	3 × 10^−8^	–	*HSPA5*	WM	*
10	94839642	rs2068888	A/G	0.45	−0.13 (0.02)	3 × 10^−10^	–	*FRA10AC1*	GM	*
10	98115019	rs41306852	A/G	0.02	0.42 (0.08)	5 × 10^−8^	–	*OPALIN*	WM	*
10	105454043	rs2863994	T/G	0.51	0.12 (0.02)	4 × 10^−9^	16	*SH3PXD2A*	GM and WM, GM	*
10	134573767	rs12258248	A/G	0.75	−0.22 (0.02)	4 × 10^−20^	3	*INPP5A*	WM, GM and WM	^19–22,24^
11	32758240	rs10767960	A/G	0.54	−0.12 (0.02)	1 × 10^−8^	17	*EIF3M*	GM and WM	*
12	32526829	rs6488048	T/C	0.65	0.13 (0.02)	7 × 10^−9^	18	*ENSG00000274964*	WM	*
12	106476805	rs12146713	T/C	0.91	−0.25 (0.04)	5 × 10^−12^	1	*ENSG00000257890*	WM, GM and WM	^18,23^
12	107349294	rs2287163	T/C	0.37	0.13 (0.02)	5 × 10^−10^	15	*TMEM263*	WM	*
14	73297741	rs2215590	T/C	0.25	0.14 (0.02)	3 × 10^−9^	8	*DPF3*	WM, GM and WM	*
14	88433660	rs413420	T/C	0.52	−0.16 (0.02)	7 × 10^−14^	23	*GALC*	WM, GM and WM	^22^
15	71169352	rs2031017	A/T	0.40	−0.14 (0.02)	1 × 10^−11^	9	*LARP6*	WM, GM and WM	^18^
16	30122181	rs536906899	CAA/C	0.40	−0.12 (0.02)	4 × 10^−8^	39	*MAPK3*	GM	*
16	90051269	rs76839250	A/G	0.91	0.25 (0.04)	1 × 10^−11^	6	*DEF8*	GM, GM and WM	^18^
17	19889274	rs111513543	A/T	0.64	−0.13 (0.02)	8 × 10^−9^	30	*AKAP10*	WM, GM and WM	*
17	27962571	17:27962571	G/GCC	0.47	0.14 (0.02)	8 × 10^−10^	10	*SSH2*	GM, GM and WM	*
17	44305199	rs2260227	T/C	0.78	−0.48 (0.03)	9 × 10^−83^	5	*MAPT*	WM, GM and WM, GM	^17–24^
17	73873656	rs1105917	T/C	0.15	0.23 (0.03)	8 × 10^−15^	4	*TRIM47*	GM, GM and WM	*
18	53277143	rs763283047	CCT/C	0.66	0.12 (0.02)	4 × 10^−8^	18	*LINC01415*	WM	*
19	31036276	19:31036276	A/AC	0.16	0.15 (0.03)	3 × 10^−8^	7	*ZNF536*	GM and WM	*
19	45416178	rs483082	T/G	0.23	0.15 (0.02)	1 × 10^−10^	4	*APOE*	GM and WM, WM	^18^
20	30336992	rs6060924	A/G	0.71	0.14 (0.02)	4 × 10^−10^	9	*BCL2L1*	GM	^18^
22	38457329	rs738442	T/C	0.38	−0.21 (0.02)	3 × 10^−23^	23	*PICK1*	WM, GM and WM	^18,23,24^
X	13891499	rs2188767	A/G	0.42	0.11 (0.02)	9 × 10^−10^	1|10	*GEMIN8*	GM, GM and WM	*
X	107888149	X:107888149	CAA/C	0.74	−0.11 (0.02)	2 × 10^−8^	–	–	GM	*
X	133781440	X:133781440	CTG/C	0.29	−0.11 (0.02)	2 × 10^−8^	41|60	–	GM	*
XY	2149565	rs34250447	T/C	0.74	−0.25 (0.02)	7 × 10^−24^	54	*DHRSX*	WM, GM and WM, GM	*

For each of the 59 discoveries across the 3 BAG traits, only the strongest variant–phenotype association is shown. The ‘Phenotype(s)’ column lists all BAG traits with significant locus associations, with the strongest listed first. For indels, the A1 and A2 alleles are truncated to three nucleotide bases. The Beta, s.e. and *P* values were derived from two-sided linear regression models using PLINK, and were meta-analyzed using inverse-variance weighting in METAL. Position indicates the base-pair position of the index variant. ID indicates the identifier of the index variant. A1 indicates the effect allele; A2 indicates the other allele. Credible indicates the number of variants in the 95% credible set identified using SBayesRC (susieR sets for X and XY; multiple signals are separated by pipe symbol ‘|’). Prioritized gene indicates the gene selected using our gene prioritization procedure. Phenotype(s) indicates traits with genome-wide significant associations at this locus (GM BAG, WM BAG and combined GM and WM BAG), with the trait with the strongest association mentioned first. Ref. indicates prior studies reporting this locus; * indicates new.

We observed most index variants in intronic regions of protein-coding genes. ANNOtate VARiation (ANNOVAR) enrichment tests confirmed that variants in high LD with the lead variants were underrepresented in intergenic regions and overrepresented in 3′-UTR, 5′-UTR, intronic, exonic noncoding RNA and intronic noncoding RNA regions (Supplementary Fig. 24 and Supplementary Table 14).

### Fine-mapping and gene prioritization

To identify putative causal genes, we used several fine-mapping, functional annotation and transcriptomic analyses that integrate information from multiple omics resources. For each genome-wide significant locus, we (1) constructed 95% credible sets of variants that likely include the causal variant using SBayesRC^40,41^, complemented by susieR and FINEMAP^42,43^; (2) physically mapped credible variants to genes using ANNOVAR^44^; (3) predicted the transcript consequences of nonsynonymous exonic variants and scored their deleteriousness using combined annotation dependent depletion (CADD)^45^; (4) mapped variants to genes using expression quantitative trait locus (eQTL) lookup in 49 Genotype-Tissue Expression (GTEx) Project v.8 tissues^46^; (5) conducted summary-data-based Mendelian randomization (SMR)^47^ with the RNA sequence (RNA-seq) data of 2,865 brain cortex samples^48^ to test for mediation through gene expression and splicing; and (6) calculated polygenic priority scores (PoPS)^49^ that incorporate data from single-cell RNA-seq datasets, curated biological pathways and protein–protein interaction networks. We integrated these results to compute a gene priority score and selected the most plausible candidate per locus (Methods). Figure 2 shows an overview of the analysis workflow; full results are found in Supplementary Table 13 (with further details in Supplementary Tables 14–24). The key findings are summarized below.

Across the 59 discovered loci, SBayesRC genome-wide fine-mapping produced, on average, the smallest 95% credible sets (median = 9 variants), compared to region-specific approaches using susieR (median = 34 variants) and FINEMAP (median = 36 variants). susieR and FINEMAP showed strong concordance, with a median overlap of 97.2%. Variants from SBayesRC were included in the susieR and FINEMAP credible sets at median rates of 75.0% and 80.0%, respectively. Most loci contained a single signal, although susieR and FINEMAP identified four loci with potential secondary signals. Estimated regional heritability ranged from 0.03% to 0.67%, reflecting modest variant effects, with few exceptions.

We observed the strongest association at locus 17q21.31 (index variant: rs2260227, *P* = 9.4 × 10^−83^), which tags a well-known 900-kb inversion polymorphism^50,51^. Consistent with the strong LD cluster in the inverted region^50^, the region-specific fine-mapping approaches yielded large credible sets of variants (>1,500 variants). A National Human Genome Research Institute GWAS Catalog search^52^ revealed associations with many locus-associated traits, including educational attainment^53^, depressed affect^54^, alcohol consumption^55^, sleep duration^56^, lung function^57^, male puberty timing^58^, age at onset of menarche^57^ and AD^59^. The region spans multiple genes, including *MAPT, STH*, *KANSL1* and *CRHR1*. Several genome-wide significant variants in these genes are GTEx single-tissue and multi-tissue eQTLs (Supplementary Tables 21 and 22). SMR analyses implicated expression and splicing of *MAPT* and *KANSL1* (along with other genes) in mediating variant effects on BAG (Supplementary Tables 19 and 20). We also identified credible exonic variants causing amino acid changes (Supplementary Table 18); notably, rs17651549 (*P* = 1.5 × 10^−81^), which had the highest deleteriousness (CADD score = 34), results in an arginine-to-tryptophan substitution at MAPT protein position 370. *MAPT* encodes the well-known tau protein implicated in AD and other neurodegenerative diseases^60^. Altogether, we prioritized *MAPT* as the most likely causal gene for brain aging at this locus.

In 6 regions, all fine-mapping methods yielded 95% credible sets with fewer than 10 likely causal variants. One locus, with only a single credible variant (rs12146713, posterior inclusion probability (PIP) > 0.99; *P* = 4.7 × 10^−12^), lies in an intron of *NUAK1* at 12q23.3 and tags a multi-tissue eQTL for the long noncoding RNA gene *Lnc-NUAK1-1*, expressed in the brain cortex and cerebellum. GWAS Catalog matches link this region to cortical thickness^61^, surface area^62^ and subcortical volume^61^.

A second locus, led by rs1452628 (*P* = 2.5 × 10^−19^), with up to 7 credible variants, refers to an intergenic region at 1q41, 41 kb upstream of *KCNK2*, encoding a potassium channel subunit. *KCNK2* is also the prioritized gene supported by the GTEx and PoPS analyses. KCNK2 has been implicated in neuroinflammation, blood–brain barrier dysfunction, and cerebral ischemia^63,64^. GWAS Catalog matches include associations with cortical thickness^61^, surface area^62^ and sulcal opening^65^.

The third locus refers to the well-known apolipoprotein E (*APOE*) gene region, led by rs483082 (*P* = 1.0 × 10^−10^), with four credible variants. The *APOE* ε4 allele, defined by rs429358 and rs7412, is the strongest known genetic risk factor for AD. Notably, the exonic variant rs429358 was identified by SBayesRC as the most likely causal variant (PIP = 0.62).

We also discovered several novel loci that offer new insights into the mechanisms of brain aging. Among these loci, one is led by rs2215590 (*P* = 2.9 × 10^−9^) and maps to an intronic region of *DPF3*, which encodes double PHD fingers 3. Supported by GTEx eQTLs and PoPS, *DPF3* was prioritized as the most likely causal gene. *DPF3* also represents the prioritized gene supported by the GTEx eQTL lookup and PoPS analyses. GWAS Catalog matches link this locus to pulse pressure^66^ and serum urate levels^67^. DPF3 serves as a subunit of the neuron-specific chromatin remodeling nBAF complex, which is crucial for neurogenesis and neurodevelopment^68^.

Another novel locus, led by rs776970253 (*P* = 2.4 × 10^−9^), implicates an intronic region of *TNIK*, encoding TRAF2-interacting and NCK-interacting kinases. Notably, TNIK has been recognized for its role in several biological pathways linked to the hallmarks of aging and has been identified as a promising drug target to improve neuronal health^69^.

Altogether, by integrating fine-mapping, functional annotation and transcriptomic data, we prioritized several genes potentially involved in brain aging, thereby offering new, testable hypotheses about its biological underpinnings.

### Polygenic score analysis

To evaluate the predictive power of the genetic variants identified in our GWAS, we performed PGS analyses using SBayesRC (Supplementary Table 25). Compared to previous reports (~2% prediction accuracy)^23^, our PGS showed substantially improved performance. Using the discovery GWAS data alone (*n* = 32,634), PGS explained 4.1% (GM BAG) to 7.0% (WM BAG) of the phenotypic variance in the European-ancestry replication sample. Incorporating the meta-analysis results (*n* = 52,890, excluding 2,000 test individuals) further improved variance explanation to 6.8% (GM BAG) and 10.3% (WM BAG). As expected, prediction accuracy was lower in the non-European UKB replication samples, explaining 0.4–3.2% of the BAG variance in African (AFR) ancestry (*n* = 337), 3.1–3.9% in Central/South Asian (CSA) ancestry (*n* = 638) and 4.1–9.1% in East Asian (EAS) ancestry (*n* = 291) individuals.

### Gene-based analysis

**Extended Data Fig. 6:**
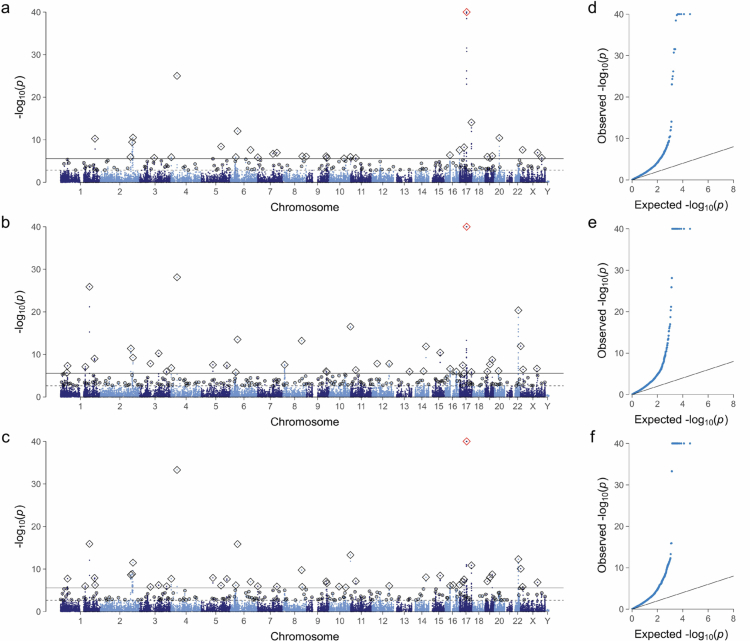
Gene-based association results for brain age gap traits. Manhattan plots (**a-c**) and quantile-quantile (QQ) plots (**d-f**) showing the results of the gene-based association analyses for the three brain age gap traits (*n* = 54,890 individuals of European ancestry). Manhattan plots display the *P* values (-log_10_ scale) of the tested genes on the y-axis and base-pair positions (gene start coordinates) along the chromosomes on the x-axis. In total, 18,639 protein-coding genes (RefSeq assembly GRCh37.p13, 09-05-2019) have been included. The solid horizontal line reflects the Bonferroni-corrected level of significance. The dashed horizontal line reflects the FDR-corrected level of significance. Diamonds and circles highlight the index gene in each genomic locus (diamonds: Bonferroni-significant index gene; red diamonds: Bonferroni-significant index gene exceeding the y-axis limit; circles: FDR-significant index gene). Genes within 3,000 kb of each other are considered to belong to the same locus and share the same index gene. Quantile-quantile plots show the observed *P* values from the association analysis vs. the expected *P* values under the null hypothesis of no effect (-log_10_ scale). For illustrative reasons, the y-axis has been truncated at *p* = 1.0e-40. **a,d** grey matter brain age gap (Manhattan and QQ); **b,e** white matter brain age gap (Manhattan and QQ); **c,f** combined brain age gap (Manhattan and QQ).

To assess the contribution of protein-coding genes, we performed gene-based association analyses using GCTA fastBAT^70^. Gene-based analyses aggregate variant-level signals across genes, reducing the multiple-testing burden. We tested 18,639 genes and identified 528, 886 and 776 genes significantly associated (FDR < 0.05) with the GM, WM and combined GM and WM BAG, respectively. To define independent loci, we applied *P* value-informed clumping to genes within 3,000 kb, yielding 151 loci, 230 loci and 203 loci per trait, of which 285 were unique (Supplementary Table 26 and Extended Data Fig. 6). The strongest signal was again observed at 17q21.31 covering *MAPT*. In total, gene-based analyses provide evidence for an extended set of genomic loci involved in human brain aging.

### Pathway analysis

To gain deeper insight into the biological mechanisms underlying brain aging, we performed gene set enrichment analyses using GOfuncR^71^, testing for the enrichment of Gene Ontology (GO) terms—sets of genes known to serve a common biological function^72^. After correcting for hierarchical dependencies (Methods), we identified 25 significant GO terms (Supplementary Table 27). Analyses highlighted immune-related and pathogen-related processes in brain aging, with significant enrichments for the major histocompatibility (MHC) protein complex (GO:0042611), peptide antigen binding (GO:0042605) and regulation of viral transcription (GO:0046782). Further significant terms, such as positive regulation of neurogenesis (GO:0050769) and regulation of axon extension (GO:0050769) align with the conceptualization of BAG as a neurodevelopmental marker. We also observed enrichment for small GTPase binding (GO:0031267l), a superfamily of evolutionary conserved proteins that act as biological timers of essential cellular processes^73^, including cell differentiation, proliferation and signal transduction^74^. Several small GTPase proteins are implicated in premature senescence^75,76^.

### Genetic correlations with other complex traits

To assess a potential shared genetic architecture between BAG and other traits, we applied bivariate LDSC^36,37^ to GWAS summary statistics, calculating genetic correlations with 38 commonly studied mental and physical health traits (Supplementary Table 28)^77–79^, as well as 989 heritable traits from a broader set of GWAS^80^.

**Fig. 4:**
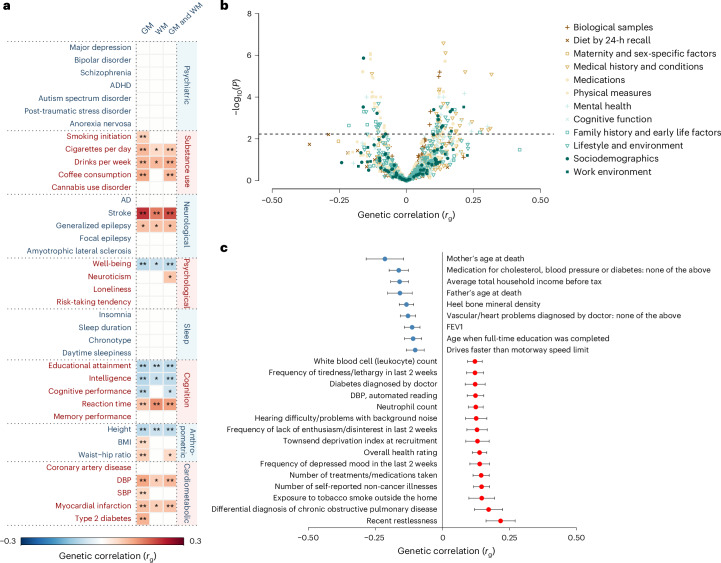
Genetic correlations between BAG and a wide range of complex traits. **a**, Genetic correlation matrix between BAG (columns) and 38 selected phenotypes from different health domains (rows). *P < 0.05 (nominal significance). **FDR < 0.05 (level of significance after correction for multiple testing). **b**, Volcano plot showing the magnitude (*x* axis) and significance (*y* axis) of LDSC-based genetic correlations between GM BAG and 989 traits, whose summary statistics were provided in ref. ^80^. The dashed horizontal line indicates the FDR-adjusted level of significance. All *P* values are two-sided. **c**, Forest plot showing the genetic correlation coefficients and standard errors for a subset of 23 exemplary traits that showed significant genetic correlations with GM BAG.

Among the 38 selected traits, 17 showed significant correlations (FDR < 0.05) with at least 1 BAG phenotype (Fig. 4a and Supplementary Table 29). GM BAG showed the highest number of associations (17) relative to WM (4) and combined GM and WM (11) BAG. Notable associations for GM BAG included substance use (cigarettes per day: *r*_g_ = 0.134), neurological (stroke: *r*_g_ = 0.217), psychological (well-being: *r*_g_ = 0.100), cognition-related (educational attainment: *r*_g_ = −0.083), anthropometric (body mass index (BMI): *r*_g_ = 0.075) and cardiovascular and metabolic syndrome traits (DBP: *r*_g_ = 0.115).

Similarly, for the 989 traits, we found 118, 7 and 48 significant associations (FDR < 0.05) for GM, WM and combined GM and WM BAG, respectively (Figs. 4b,c and Supplementary Table 30). BAG showed significant genetic correlations with parental longevity (mother’s age at death, *r*_g_ = −0.214; father’s age at death, *r*_g_ = −0.158), socioeconomic status (average total household income before tax, *r*_g_ = −0.160), mental health (frequency of tiredness/lethargy in the last 2 weeks, *r*_g_ = 0.122), medical conditions (vascular/heart problems diagnosed by doctor: high blood pressure, *r*_g_ = 0.122), cognitive function (snap game—mean time to correctly identify matches, *r*_g_ = −0.069), respiratory traits (forced expiratory volume in 1 s (FEV1), best measure, *r*_g_ = −0.128), blood markers (white blood cell (leukocyte) count, *r*_g_ = 0.121) and early life exposures (maternal smoking around birth, *r*_g_ = 0.099), among others (Supplementary Table 30). These results suggest genetic overlap between BAG and a broad range of health-related traits.

### Mendelian randomization analyses

**Extended Data Fig. 7:**
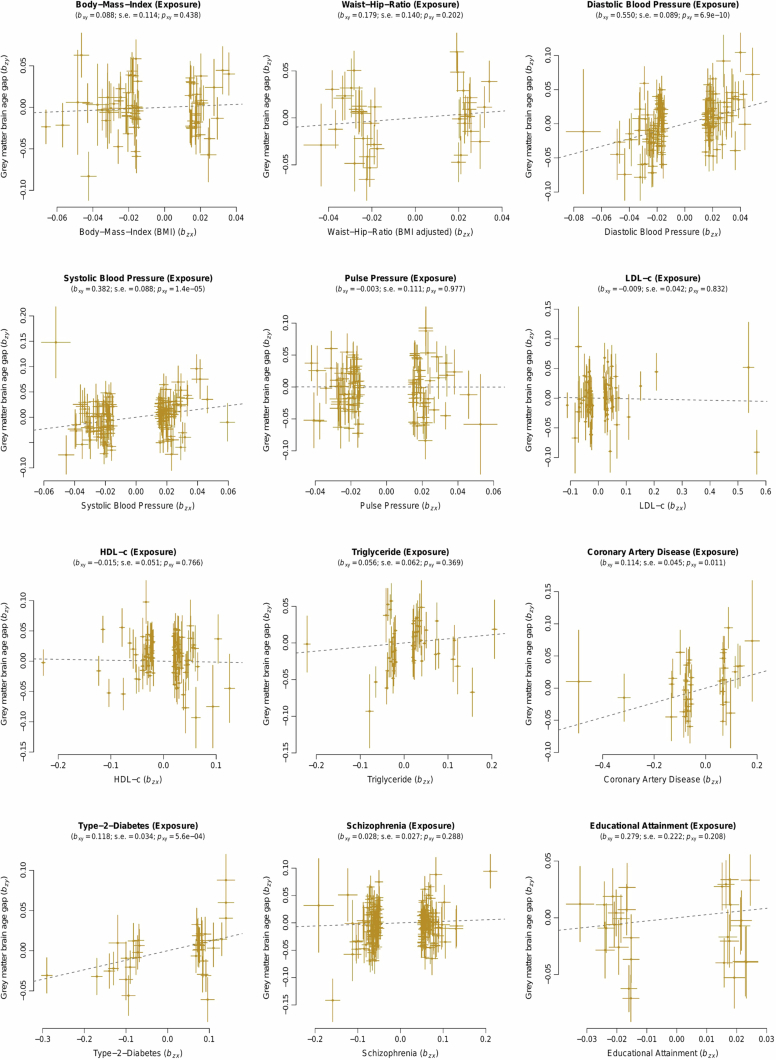
Mendelian randomization analyses linking modifiable risk factors to brain age gap. Results of the generalized summary-data-based Mendelian randomization (GSMR) results for 12 risk factors (exposures) and combined brain age gap (outcome). Each plot shows multiple genetic variants serving as instruments to test for causality between the exposure and outcome variable. Under a causal model, variant effects on the outcome (*b*_zy_; y-axis) are expected to be linearly proportional to the variant effects on the exposure variable (*b*_zx_; x-axis). The ratio between *b*_zy_ and *b*_zx_ provides an estimate of the mediation effect of x on y (*b*_xy_). Variants flagged for potential horizontal pleiotropy were excluded using the HEIDI-outlier method. s.e. standard error of the mediation effect; *p*_xy_ *P* value of the mediation effect.

We used two-sample generalized summary-data-based Mendelian randomization (GSMR)^81^ to investigate the potential causal effects of 12 modifiable risk and resilience factors on BAG. These included BMI, waist–hip ratio adjusted for BMI, low-density lipoprotein cholesterol, high-density lipoprotein cholesterol, triglycerides, systolic blood pressure (SBP), DBP, pulse pressure, type 2 diabetes, coronary artery disease, schizophrenia and years of education (Supplementary Table 31). Across all BAG traits, we found significant effects of DBP (combined BAG: *β*_xz_ = 0.550, *P* = 6.9 × 10^−10^) and SBP (combined BAG: *β*_xz_ = 0.382, *P* = 1.4 × 10^−5^), indicating that a one standard deviation increase in blood pressure causally contributes to an ~0.5-year increase in BAG (Supplementary Figs. 43–48 and Extended Data Fig. 7). We also observed significant effects of type 2 diabetes (combined BAG: *β*_xz_ = 0.118, *P* = 5.6 × 10^−4^) and coronary artery disease (combined BAG: *β*_xz_ = 0.114, *P* = 0.01)^82^. These findings were largely confirmed by nine alternative MR analyses, except for coronary artery disease (Supplementary Table 32).

Reverse GSMR analyses (Supplementary Table 33) indicated potential negative feedback effects of WM BAG on SBP, DBP and pulse pressure (all *β*_xz_ = −0.009, all *P* ≤ 3.2 × 10^−3^), which is consistent with lower blood pressure in late life and frailty^83^. Additionally, reverse GSMR analyses hinted at BAG effects on elevated low-density lipoprotein cholesterol and increased risk for coronary artery disease, although these were not supported by other MR methods.

### Polygenicity and projection of discoveries to future GWAS

**Fig. 5:**
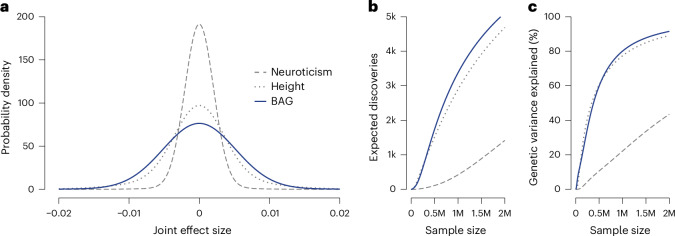
Genetic effect-size distribution analysis of BAG. **a**, Results are shown for combined GM and WM BAG, with neuroticism and standing height included as reference traits; effect-size distributions of the underlying susceptibility variants are shown; wider tails indicate a greater proportion of large-effect variants. **b**, Predicted number of genome-wide significant loci as a function of sample size. **c**, Proportion of genetic variance explained by genome-wide significant loci as a function of sample size.

To quantify the BAG degree of polygenicity and estimate discovery potential in future GWAS, we used GENESIS^84^ to estimate the number of underlying susceptibility variants and their effect sizes. We also selected height and neuroticism as benchmark traits because of their distinct degrees of polygenicity^84–87^. The number of susceptibility variants was estimated at 8.7k (s.e. = 1.8k) for GM, 9.8k (s.e. = 1.3k) for WM and 11.0k (s.e. = 1.3k) for combined GM and WM BAG (Supplementary Table 34). For comparison, height showed 12.6k (s.e. = 1.3k) and neuroticism 16.2k (s.e. = 1.2k) susceptibility variants. Effect-size distributions (Fig. 5a) revealed that BAG includes a larger proportion of variants with stronger effects compared to neuroticism, but similar to height. Our projections also suggest rapid growth in the number of BAG discoveries with increasing sample size (Fig. 5b). Approximately 1 million individuals are needed to explain 80% of the SNP-based heritability for BAG via genome-wide significant variants (Fig. 5c), a threshold comparable to height but lower than for neuroticism (~6 million). These findings suggest that while BAG is genetically complex, its relatively lower polygenicity enhances discovery prospects in future studies.

## Discussion

In this study, we leveraged genomic and neuroimaging methods to establish BAG as a promising biomarker of aging with potential utility for therapeutic discovery. Using machine learning and MRI, we derived highly reliable brain age estimates that capture aging-related structural patterns across the brain. BAG showed robust phenotypic associations with many health traits and was substantially heritable, with 23–29% of variance attributable to common genetic variation. We identified 59 independent genome-wide significant loci, of which 39 are novel. The genomic signals unveiled enriched biological pathways, for example, immune-system-related processes and small GTPase binding, prompting further mechanistic exploration. Through genetic correlations, we demonstrated shared genetic influences between BAG and a broad spectrum of physical and mental health traits. Mendelian randomization supported a causal role of elevated blood pressure and diabetes in accelerated brain aging. Finally, BAG showed a relatively low degree of polygenicity, which increases the likelihood of variant discovery in future studies.

Brain aging is not a uniform process; rather, it encompasses diverse aspects of structural and functional change. Studying distinct aspects of brain aging has been advocated to increase the yield of biologically meaningful insights^18^. In this study, we estimated separate BAG scores for GM and WM, alongside a composite measure. While all three showed similar age prediction accuracy and test–retest reliability, GM BAG exhibited stronger phenotypic and genetic associations, potentially reflecting a closer link to health-related outcomes. One explanation is that our volumetric approach to brain age estimation captures biologically meaningful differences more effectively in GM—tied to dendritic complexity and synaptic density—than in WM, which depends on microstructural features less directly represented in bulk volume. As such, GM BAG may better reflect deviations from normative aging across diverse health domains.

While brain aging follows multiple biological trajectories, differential aging rates across systems underscore the need to conceptualize bodily aging as a heterogeneous process^6^. Consistent with this, previous research showed weak correlations between biological age measures derived from telomere length, epigenetic clocks, transcriptomics and immunometabolic markers^88^. Similarly, in our previous work, we found modest correlations between brain age, epigenetic clocks, skin age, clinical biological age composites and subjective age^89,90^. Although combining multiple biomarkers may improve chronological age prediction, it may also obscure biologically meaningful deviations at the domain level^18^. Recognizing this heterogeneity could help refine aging biomarker panels, and improve specificity and prognostic value for disease risk.

Several GWAS of BAG have been conducted previously^15,17–24^; however, few have incorporated extensive post-GWAS analyses. Leveraging increased statistical power, we present the most comprehensive genetic analysis of BAG to date, identifying 26, 34 and 39 loci across GM, WM and combined GM and WM BAG—59 in total, of which 39 are new. This surpasses the fewer than ten loci reported per phenotype in earlier studies. Expanding prior fine-mapping efforts^22^, we constructed credible sets of likely causal variants (median = 9 per locus) and developed PGS explaining 7–10% of BAG variance, up from 2% previously^23^. Additionally, we here report results in groups of non-European ancestry, showing reduced yet significant polygenic predictions in AFR, CSA and EAS populations. We also identified new gene sets linked to neurodevelopment, immune function and signal transduction. Extending previous Mendelian randomization efforts^21–24^, we confirmed a causal role of diabetes in BAG and newly identified blood pressure as a risk factor. Moreover, we expanded genetic correlations^15,21–24^ and identified over 140 BAG associations across health domains. Finally, we introduced genetic effect-size distribution modeling, estimating ~8,700–11,000 contributing variants and providing key projections for discovery potential in future studies.

In addition to these new insights, our study also reinforces previously reported genetic associations. We confirmed the inversion locus at 17q21.31 as the strongest genetic contributor to BAG^17–24^, with *MAPT*—a gene encoding the tau protein implicated in AD—prioritized as the most likely causal gene. We also identified the well-known AD risk gene *APOE* and other apolipoprotein genes. The presence of both tau-related and apolipoprotein-related signals suggests that key hallmarks of AD are reflected in accelerated brain aging, reinforcing BAG’s relevance as a marker for neurodegenerative risk^91^.

Our findings suggest that BAG integrates signals from neurodevelopmental and neurodegenerative processes, lifestyle factors, vascular and metabolic health, and immune function. This supports the idea that BAG is not purely a neurodegenerative marker but reflects genetic susceptibility, systemic health and environmental exposures. This supports viewing BAG as composite indicator shaped by aging, disease susceptibility and lifestyle exposures.

**Extended Data Fig. 8:**
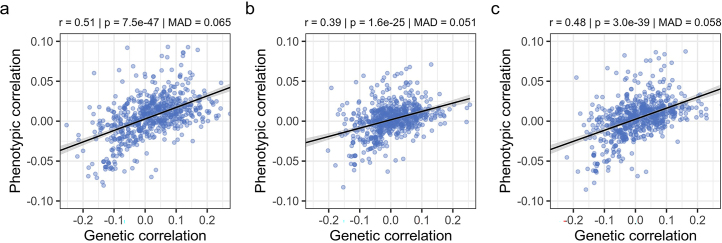
Relationship between genetic and phenotypic correlations of brain age gap with other complex traits. This figure compares genetic and phenotypic correlation coefficients between brain age gap and 673 other complex traits. Genetic correlations were estimated using GWAS meta-analysis summary statistics for BAG (*n* = 54,890, European ancestry), while phenotypic correlations were calculated in the UK Biobank discovery sample (*n* = 32,634, European ancestry) using PHESANT. Summary statistics for other UK Biobank traits were obtained from Neale et al. (10.5281/zenodo.7186871). Results indicate a positive association between genetic and phenotypic correlation estimates. r: product-moment correlation coefficient, p: *P* value, MAD: mean absolute difference. (**a**) grey matter brain age gap, (**b**) white matter brain age gap, (**c**) combined brain age gap.

By reporting both phenotypic and genetic correlations for BAG, our study allows a direct comparison between the two. We observed a positive relationship between genetic and phenotypic correlations (Extended Data Fig. 8), with correlation coefficients ranging from 0.39 (WM BAG) to 0.51 (GM BAG). Although weaker than previously reported, probably because of the restricted range of correlation strengths in our dataset, the small mean absolute difference between genetic and phenotypic estimates suggests that phenotypic correlations approximate genetic ones and vice versa^92,93^.

A future research direction is to explore how model precision affects the genetic and phenotypic associations of BAG^94^. Improved age prediction accuracy may enhance heritability estimates and genetic signals, but it could also obscure meaningful deviations from normative aging. Future studies may investigate this balance to understand the trade-off between predictive performance and biological interpretability.

The current study has several limitations. First, the gene prioritization techniques face challenges in pinpointing causal genes^49^, particularly in loci characterized by high gene density and complex linkage structures. To streamline interpretation, we adopted a winner-takes-all approach, prioritizing a single candidate gene per locus. However, this may overlook other plausible genes with similarly high prioritization scores. Second, BAG was estimated from cross-sectional data, typically interpreted as accelerated or decelerated aging. However, an alternative view posits BAG as stable, early-emerging individual differences that persist into old age^95^. Third, although we used an ensemble of three machine learning models, expanding the number and diversity of models may further enhance prediction by leveraging complementary strengths. Fourth, polygenic overlap was assessed using genetic correlations, which do not capture shared variants with opposing effects. Future studies could apply tools such as MiXeR to quantify genetic overlap by considering mixtures of variant effects^96^. Fifth, polygenicity estimates probably underestimate the true number of contributing variants because models may classify those with very small effects as null. Finally, as our primary GWAS was based on individuals of European ancestry, PGS predicted less accurately in groups of non-European ancestry, limiting generalizability across ancestries. This reflects known transfer challenges due to differences in allele frequencies, LD and environmental context. Expanding genomic data and sample sizes in diverse populations will be essential to improve accuracy and broaden the applicability of brain age genetics.

In conclusion, our study refines the genetic architecture of BAG and its relationships to other traits. We added 39 new genetic loci and nominated plausible candidate genes, including *DPF3*, which is implicated in neurodevelopment, and *TNIK*, which is linked to neuronal health and aging-related diseases. This will facilitate further work on the pathway mechanisms of BAG and potential therapy targets.

## Methods

### Ethical approval

This study used individual-level data from the UKB (www.ukbiobank.ac.uk) and LIFE-Adult (www.uniklinikum-leipzig.de/einrichtungen/life)^26,29,30^. Both studies were conducted in accordance with applicable ethical regulations and the principles of the Declaration of Helsinki (2008). The UKB received approval from the North West–Haydock Research Ethics Committee (ref. nos. 11/NW/0382, 16/NW/0274, 21/NW/0157). LIFE-Adult was approved by the Ethics Committee of Leipzig University (ref. nos. 263–2009-14122009, 263/09-ff, 201/17-ek). All participants provided written informed consent. LIFE-Adult participants received a fixed compensation of 20 EUR per visit. UKB participants could claim travel reimbursement.

### Statistics and reproducibility

This study presents results from a GWAS alongside a broad set of post-GWAS analyses, including fine-mapping, polygenic scoring, genetic correlation and Mendelian randomization. To enhance transparency and reproducibility, we have provided all analysis scripts, conda environments and software details in a public GitHub repository (https://github.com/pjawinski/ukb_brainage). Analyses were run on Debian GNU/Linux 11 (kernel 5.10.0-23-amd64). Unless stated otherwise, all *P* values are two-sided. Associations with *P* < 0.05 were considered nominally significant; Bonferroni correction and FDR control according to the Benjamini–Hochberg procedure were used to adjust for multiple testing. No formal power calculation was used to predetermine sample size. Instead, we included all eligible individuals from the UKB and LIFE-Adult who passed predefined quality control criteria.

Data exclusions were limited to prespecified quality control steps, described in detail elsewhere in the Methods. Analytical assumptions were addressed at each stage of the analysis. In cross-trait association testing, regression models were automatically selected based on the type of variable, with continuous variables normalized to meet distributional assumptions. In the GWAS, standard variant-based and sample-based quality control was applied; LDSC confirmed that the test statistic inflation was driven by polygenicity rather than confounding. This study was observational and nonexperimental; thus, participants were not randomly assigned and no blinding was applied. We report how our target samples were defined, all data exclusions, quality control procedures and all measures used in the study. A full list of UKB variables is provided in the UKB data dictionary (https://biobank.ndph.ox.ac.uk/showcase/) and LIFE-Adult data portal (https://ldp.life.uni-leipzig.de/).

### Sample characteristics

Participants were drawn from the UKB under application no. 423032. A detailed description of the UKB study design and quality control methods has been published previously^26^. For our discovery sample, participants were drawn from the UKB January 2020 imaging release (v.1.7). These data contained 40,681 participants with structural T1-weighted MRI scans (UKB data-field 20252). Scans in folders labeled ‘unusable’ were excluded, leaving 39,679 participants. Voxel-based morphometry preprocessing was successfully completed for 39,677 MRI scans (see the ‘MRI preprocessing’ section of the Methods). Analyses were restricted to participants whose self-reported sex matched the genetic sex (data-fields 31 and 2200), without sex chromosome aneuploidy (data-field 22019) and who were no outliers in heterozygosity and missingness (data-field 22027). We only included unrelated participants as suggested by pairwise kinship coefficients below 0.0442 (precalculated coefficients retrieved using ‘ukbgene rel’). We included participants of White British ancestry (data-field 22006), yielding a final discovery sample of 32,634 participants (17,084 female, age range = 45.2–81.9 years, mean age = 64.3 years).

For replication, we selected all remaining individuals without White British ancestry from the UKB January 2020 release (*n* = 4,870). Applying the same inclusion criteria, we added European and non-European UKB participants with imaging data released until May 2024 (v.1.10), yielding 25,668 individuals. None of them were related to the discovery participants. We included individuals with valid ancestry assignment from the Pan-ancestry return (no. 2442; https://pan.ukbb.broadinstitute.org/). This resulted in 337 African, 94 Admixed American, 638 Central/South Asian, 291 East Asian, 20,423 European and 98 Middle Eastern ancestry participants. In total, we included 21,881 UKB participants for replication (11,451 female, age range = 45.5–81.9 years, mean age = 67.1 years). From the LIFE-Adult study^29,30^, we included another 1,833 unrelated participants of European ancestry (888 female, age range = 45.2–80.3 years, mean age = 65.3 years) with available T1-weighted MRI and genotype data, selected to match the UKB age range^97^. Altogether, the final replication sample included 23,714 participants (12,339 female, age range = 45.2–81.9 years, mean age = 67.0 years) from 7 subsamples.

### MRI data acquisition

The UKB imaging acquisition protocol and processing pipeline have been detailed previously (http://biobank.ctsu.ox.ac.uk/crystal/refer.cgi?id=1977). Brain MRI data were acquired at four UKB imaging centers (Cheadle, Newcastle, Reading and Bristol) on Siemens Skyra 3T MRI scanners (Siemens Healthcare) running the VD13A SP4 software, with a standard 32-channel radiofrequency head coil. We used T1-weighted structural MRI scans (UKB data-field 20252) acquired using a 3D magnetization-prepared rapid gradient-echo (MPRAGE) sequence in the sagittal plane, with a voxel size of 1 × 1 × 1 mm, 208 × 256 × 256 acquisition matrix, 2,000-ms repetition time (TR), 2.01-ms echo time (TE), 880-ms inversion time (TI), 6.1-ms echo spacing, 8 ° flip angle, a bandwidth of 240 Hz per pixel, an in-plane acceleration factor of *R* = 2 and duration of 4 min 54 s.

In LIFE-Adult, brain imaging was performed on a 3T Verio MRI scanner (Siemens Healthcare) with a standard 32-channel head coil. T1-weighted images were obtained using a 3D MPRAGE sequence with a voxel size of 1 × 1 × 1 mm, 256 × 240 × 176 acquisition matrix, TR = 2,300 ms, TE = 2.98 ms, TI = 900 ms and 9 ° flip angle.

### MRI preprocessing

T1-weighted MRI scans in NIfTI format were preprocessed using the voxel-based morphometry pipeline of CAT12 (r1364, http://dbm.neuro.uni-jena.de) for SPM12 (r7487) in MATLAB R2021a (MathWorks). CAT12 preprocessing included affine and DARTEL registration to a reference brain, segmentation into GM, WM and cerebrospinal fluid, bias correction for intensity inhomogeneity and modulation to account for volume changes because of spatial registration. Images were then smoothed using an 8 × 8 × 8-mm full-width-at-half-maximum Gaussian kernel and resampled to a voxel size of 8 mm^3^. Only scans with a CAT12 overall image quality rating of less than 3.0 were retained, excluding 119 (~0.3%) of 39,677 scans from the UKB imaging release v.1.7, and 101 (0.4%) of 23,000 additional scans from release v.1.10.

### Feature set for machine learning

Machine learning features were derived from CAT12-preprocessed GM and WM segmentations. Each smoothed, resampled brain image included 16,128 voxels. Voxels without interindividual variation were excluded, yielding 5,416 GM and 5,123 WM voxels. Because of spatial correlation across voxels, we applied principal component analysis (PCA) in MATLAB to reduce dimensionality. The first 500 principal components—explaining ~90% of the total variance—were selected as features.

### Machine learning algorithms

We implemented three complementary algorithms to model age from the brain imaging data: the sparse Bayesian RVM using the MATLAB toolbox SparseBayes v.2 with the wrapper and kernel in refs. ^27,98^, and extreme gradient boosting using XGBoost v.0.82.1 in R^28^, using both decision tree (gbtree) and linear (gblinear) boosters. These algorithms were chosen for their demonstrated efficacy in previous brain age studies^5,15,99,100^. XGBoost was configured with a learning rate of *η* = 0.02, 5,000 training iterations, early stopping after 50 iterations without improvement and maximum tree depth of 3. Default settings were used for all other training parameters. To exploit their complementary strengths in handling high-dimensional data, modeling linear and nonlinear relationships, and regularization, we combined all three (RVM, XGBoost tree and XGBoost linear) in an ensemble.

### Age estimation models and BAG calculation

Age estimation models were trained using PCA-derived brain imaging features to predict chronological age. Training and application were performed in the discovery sample using tenfold cross-prediction with 100 repeats. This cross-prediction approach was chosen to maximize precision and avoid bias from external datasets with differing MRI protocols, similar to previous studies^15,20–22^. The discovery sample was split into ten equally sized subsets. In each iteration, nine subsets served for model training and one for testing. PCA was performed on the training data and the transformation parameters were applied to the test set. This procedure was cycled through all ten folds, so each subset served once as the test set. The entire tenfold cross-prediction procedure was repeated 100 times, generating 100 predictions for each individual. This process was run for each tissue type (GM and WM) and model type (RVM, XGBoost tree and XGBoost linear), yielding 600 brain-predicted age estimates per individual (2 tissues × 3 models × 100 repeats). A nested tenfold cross-prediction was used to stack model-type predictions into tissue-specific ensemble estimates for GM and WM. To derive estimates for combined GM and WM, we stacked tissue-specific predictions rather than training new models on combined inputs. This yielded 100 age estimates per tissue type (GM, WM, combined), which were averaged to obtain 1 final brain-predicted age estimate per tissue type. Model performance was evaluated using the product-moment correlation coefficient (*r*), the coefficient of determination (*R*^2^) and MAE^100^.

In the replication samples, age predictions were generated using all tenfold discovery models and compared to predictions from models trained on the full discovery sample. Results were highly concordant for all three tissue types (*r* > 0.997; Supplementary Fig. 15). For improved practicability, subsequent replication analyses used models trained on the full discovery sample.

BAG was calculated as the difference between predicted brain age and chronological age as:

$${\rm{BAG}}={\hat{{\rm{A}}}}_{{\rm{brain}}}-{{\rm{A}}}_{{\rm{chron}}}$$

where BAG reflects the brain age gap estimate. Â_brain_ is the predicted (modeled) age based on an individual’s brain imaging data and A_chron_ is the actual chronological age of the individual.

Because of regression dilution, BAG is typically confounded by age, with younger individuals showing higher and older individuals lower BAG values^31^. To correct this bias, we included both age and age^2^, alongside additional covariates (sex, scanner site, total intracranial volume, genotyping array, genetic principal components), in all association analyses^31,32,100^.

### Cross-trait association analysis

We performed cross-trait association analyses using PHESANT v.1.1 (ref. ^33^), an automated pipeline for phenome-wide association analyses in the UKB. Each BAG phenotype was tested against 7,088 nonimaging UKB variables. Covariates included sex (field 31), age (derived from fields 34, 52 and 53), age^2^, scanner site (field 54) and total intracranial volume (from CAT12 segmentation). PHESANT selected regression models (linear, logistic, ordinal logistic or multinomial logistic) based on the type of variable. Continuous variables were inverse-rank-normalized before linear regression. To obtain standardized effect sizes, we calculated product-moment correlations (*r*) via corresponding *z*-statistics: $r={\mathrm{sign}}\left(\beta \right)\sqrt{{z}^{2}/({z}^{2}+(N-k-2))}$. For visualization, variables were grouped into categories based on the UKB data dictionary path. We also performed sex-stratified analyses and tested sex differences by comparing PHESANT beta coefficients in males (*β*_m_) and females (*β*_f_): $z=({{{\beta }}}_{{\rm{m}}}-{{{\beta }}}_{{\rm{f}}})/\sqrt{({\rm{s.}}{{\rm{e.}}}_{{\rm{m}}}^{2}+{\rm{s.}}{{\rm{e.}}}_{{\rm{f}}}^{2})}$. The resulting *z*-values were converted into *P* values using standard normal probabilities.

### FreeSurfer associations

To examine associations between BAG and individual brain regions, we analyzed brain measures from the FreeSurfer aparc and aseg output files (UKB data-field 20263)^35^, including surface area, cortical thickness and volume from 34 bilateral cortical and 8 bilateral subcortical regions (220 measures in total). We calculated partial product-moment correlations between BAG and brain measures, adjusting for sex, age, age^2^, scanner site and total intracranial volume. Visualizations were created using the ENIGMA toolbox v.2.0.3 for MATLAB^101^. We also performed sex-stratified analyses and tested sex differences using Fisher’s *r*-to-*z* transformation with the cocor R package^102^. Associations between brain regions and chronological age are reported in Supplementary Table 3.

### UKB genotyping and imputation

We retrieved genotype data (called: BED; imputed: BGEN v.3) from the UKB. Genotype collection, processing and quality control have been described previously^26,103^. Genotyping was performed on DNA from EDTA blood using 2 Affymetrix arrays with 95% marker overlap: the UKB BiLEVE Axiom Array (807,411 markers used in 49,950 participants) and the UKB Axiom Array (825,927 markers used in 438,427 participants). Marker-based quality control included a call rate greater than 0.90, tests for batch, plate, array and sex effects, and Hardy–Weinberg equilibrium (*P* < 1.0 × 10^−12^). Sample-based quality control excluded individuals with a missingness greater than 0.05, high heterozygosity, sex discordance or sex chromosome aneuploidy. Relatedness was inferred using KING^104^. White British ancestry (data-field 22006) was defined via self-report and genetic principal components. Genotypes were phased using SHAPEIT3 and imputed using IMPUTE4 (https://jmarchini.org/software/) with the Haplotype Reference Consortium, UK10K Project and 1000 Genomes Project Phase 3 serving as reference. Imputation yielded ~97 M markers. We selected biallelic SNPs and indels with MAF > 0.01 and INFO > 0.80. Biallelic variants were defined as those without duplicate coordinates or duplicate identifiers. This resulted in 9,669,330 variants for the discovery GWAS. In the replication samples, the number of variants passing quality control ranged between 8,345,339 (EAS ancestry) and 15,371,587 (AFR ancestry).

### LIFE-Adult genotyping and imputation

Genotype collection, processing and quality control in LIFE-Adult have been described previously^97^. DNA from peripheral blood leukocytes was genotyped on the Axiom Genome-Wide CEU 1 Array (Applied Biosystems) (587,352 markers). Marker-based quality control removed variants with call rate lower than 0.97, Hardy–Weinberg equilibrium *P* < 1.0 × 10^−6^ or plate effects *P* < 1.0 × 10^−7^. Sample quality control excluded individuals with Dish QC < 0.82, missingness > 0.03, sex discordance or cryptic relatedness. Genotypes were phased using SHAPEIT and imputed with IMPUTE2 using the 1000 Genomes Project Phase 3 as reference. This yielded 85,063,807 markers in 7,776 individuals. Quality control after imputation (MAF > 0.01, INFO > 0.8) left 9,472,504 biallelic SNPs or indels that also passed UKB quality control for inclusion in the meta-analysis.

### Control for population structure

In the discovery sample, we calculated 20 genetic principal components using the randomized PCA algorithm (--pca 20 approx) implemented in PLINK v.2.00a2LM^105^, based on the same variants used by the UKB group (resource 1955; 146,988 markers passing our own quality checks). For the UKB replication samples, we used principal components from the Pan-ancestry UKB project (return 2442), using 20 components for the larger UKB European-ancestry sample and 4 for all other groups.

### Heritability and partitioned heritability

Estimates of SNP-based heritability (*h*^2^_SNP_) were derived by applying LDSC^36,37^ to our GWAS summary statistics, with precalculated LD scores and regression weights from the 1000 Genomes Project Phase 3. Analyses were limited to HapMap3 variants with MAF > 0.01, excluding the MHC region. Partitioned heritability was assessed using stratified LDSC^38^ with baseline-LD model v.2.2. We tested the 33 main annotations reported in ref. ^106^, considering annotations with an FDR < 0.05 as significant.

### Genome-wide association analysis

GWAS analyses were performed in PLINK v.2.00a2LM^105^ using allelic dosage data, including autosomal (chromosomes 1 and 22), gonosomal (chromosomes X and Y) and pseudoautosomal (chromosomes XY) variants. Dosage scales were 0–2 for diploid regions (chromosomes 1–22, chromosomes XY), 0–1 for haploid chromosome Y and 0–2 for chromosome X. We modeled additive genetic effects and used sex, age, age^2^, total intracranial volume, scanner site, type of genotyping array and the first 20 genetic principal components as covariates (4 components for the LIFE-Adult and non-European-ancestry samples).

### Genome-wide association meta-analysis

The European-ancestry GWAS results were meta-analyzed using fixed-effects inverse-variance-weighted models in METAL (v.2020-05-05)^107^. Variants with a sample size of less than 67% of the 90th percentile (adapted from LDSC)^37^ or heterogeneity *P* < 1.0 × 10^−6^ were excluded. The final GWAS meta-analysis in individuals of European ancestry included 9,628,877 variants for GM and WM BAG, and 9,628,868 variants for the combined BAG, analyzed in up to 54,890 individuals. Multi-ancestry meta-analyses were performed with MR-MEGA v.0.2 (ref. ^108^), including the White British discovery sample, six UKB replication samples (European, African, Admixed American, Central/South Asian, East Asian, Middle Eastern ancestry) and the European-ancestry LIFE-Adult cohort. Ancestry effects were modeled using three axes of genetic variation derived from allele frequency differences. For comparison, fixed-effects and random-effects meta-analyses were also conducted in GWAMA v.2.2.2 (ref. ^109^). The multi-ancestry GWAS results included 8,618,923 variants in up to 56,348 individuals.

### Identification of independent discoveries

We identified independent association signals using stepwise conditional analyses in GCTA-COJO^39^. A 10,000-kb window size and collinearity cutoff of 0.9 were applied. Multiple signals within a locus were only considered independent if the *P* value of the subsidiary signal did not increase by more than two orders of magnitude relative to its unadjusted value. Variants reaching *P* < 5.0 × 10^−8^ in the conditional analysis were considered genome-wide significant; those with *P* < 1.0 × 10^−6^ were deemed suggestive. We refer to lead variants from these signals as index variants. To identify nonredundant signals across the three BAG GWAS, all index variants were LD-clumped (*r*^2^ < 0.1, window size = 10,000 kb) using PLINK v.1.90b6.8.^105^.

### Definition of variant replication and power calculations

From the discovery GWAS, we selected index variants from genome-wide significant loci (conditional *P* < 5.0 × 10^−8^) and 45 additional suggestive loci (conditional *P* values between 1.0 × 10^−6^ and 5.0 × 10^−^^8^) for replication. Consistency between discovery and replication was tested using sign tests (binomial), based on the agreement in effect direction. Variants with a replication *P* < 0.05 (one-tailed nominal significance) were considered replicated. To estimate the expected replication yield, we performed power calculations based on standardized discovery betas, MAF and replication sample size^110^. Beta coefficients were corrected for winner’s curse^111^. Expected replications were computed as the sum of individual variant-level power estimates.

### Novelty of the discovered loci

To assess novelty, we compared our findings against nine prior BAG GWAS reporting genome-wide significant loci^15,17–24^. Using PLINK v.1.90b6, we clumped variants based on LD (*R*^2^ > 0.1, window size = 10,000 kb)^105^ and defined loci as novel if they did not cluster with previously reported variants. Parameter choices were guided by GCTA-COJO^39^ and Psychiatric Genomics Consortium studies^79,112^. Of the 59 loci identified, 39 were classified as novel, a result consistent across clumping thresholds (*R*^2^ of 0.10 or 0.05) and window sizes (10,000 kb or 3,000 kb).

### ANNOVAR enrichment test

We used the ANNOVAR (v.2017-07-17)^44^ enrichment test implemented in FUMA v.1.6.0 (https://fuma.ctglab.nl/)^113^ to evaluate whether genome-wide significant regions were enriched for specific functional annotations. All candidate variants in LD (*R*^2^ > 0.6) with independent significant autosomal variants (*P* < 5.0 × 10^−8^) were included. Candidate variants were defined as those with *P* < 0.05 and *R*^2^ > 0.60 with an independent significant variant. UKB release 2 served as the LD reference panel. If a variant had multiple annotations, each was counted separately. Enrichment was computed as the proportion of candidate variants with a given annotation relative to the proportion of variants with that annotation among all variants in the reference panel. Significance was tested using a two-tailed Fisher’s exact test.

### Credible sets of variants

We used SBayesRC, a Bayesian mixture model implemented in GCTB v.2.5.2 (refs. ^40,41^), to construct 95% credible sets of variants per locus, capturing the cumulative posterior probability of including a causal variant. Unlike region-specific fine-mapping approaches, such as susieR^42^ and FINEMAP^43^, SBayesRC jointly models multiple genomic regions alongside functional annotations. We used SBayesRC with the eigendecomposition data of LD matrices from our discovery dataset of 32,634 individuals (~9.7 M imputed SNPs), and functional annotations from the stratified LDSC baseline-LF UKB model (v.2.2)^114^. We used default settings with five mixture components (scaling factors of 0, 0.001, 0.01, 0.1 and 1%). Credible sets were assigned to a discovered locus if they contained at least 1 genome-wide significant credible variant in strong LD (*R*^2^ > 0.8) within 3,000 kb from the index variant. We report sets with PIP > 0.95 and posterior enrichment probability > 0.50. For comparison, we also applied susieR v.0.12.35 (ref. ^42^) and FINEMAP v.1.4.2 (ref. ^43^). For each locus, a 10,000-kb window was used to identify the outermost variants in LD (*R*^2^ > 0.1), defining region boundaries. LD matrices were computed using LDstore v.2.0 (ref. ^115^) in 53,074 individuals of European ancestry from the combined discovery and UKB replication sample. For FINEMAP, we allowed up to *k* = 10 causal variants per region, reporting 95% credible sets for the most probable *k* model. For susieR, we allowed up to *L* = 10 causal signals per region, reporting 95% credible sets with minimum purity greater than 0.5.

### Functional annotation of variants

Variants were annotated using ANNOVAR^44^, which assigns functional categories based on physical position relative to genes. RefSeq gene annotations (hg19) were retrieved from the UCSC Genome Browser (https://genome.ucsc.edu/)^116^. The nearest gene was identified using ANNOVAR’s default prioritization of variant function and genomic distance. The transcript consequences of nonsynonymous exonic variants were predicted; deleteriousness scores from CADD were obtained from dbnsfp35a (hg19)^45,117^.

### Gene nomination through functional annotation of credible variants

Credible variants were annotated using ANNOVAR^44^, and variant posterior probabilities were aggregated per gene implicated. Genes were then ranked according to their total variant posterior probabilities and nominated for gene prioritization. Additionally, genes implicated by nonsynonymous exonic variants were ranked based on the highest CADD Phred-scaled score among those variants.

### Gene nomination through SMR

We applied summary-data-based Mendelian randomization implemented in SMR v.1.03 (refs. ^47,48^) to test whether variant effects were potentially mediated by gene expression or splicing. SMR integrates GWAS summary statistics with omics data to prioritize gene targets and regulatory elements. It adopts the Mendelian randomization strategy by using a single genetic instrument (z) to test for pleiotropic association between gene regulation (exposure, x) and a trait of interest (outcome, y). The effect of gene regulation on a trait (*β*_xy_) is calculated as a two-step least squares estimate and defined as the ratio of the instrument’s effect on the outcome (*β*_zy_) to its effect on the exposure (*β*_zx_), that is, *β*_xy_ = *β*_zy_/*β*_yz_. To distinguish pleiotropy from linkage, SMR incorporates the HEterogeneity In Dependent Instruments (HEIDI) test, which leverages multiple instruments in the regulatory region. We used *cis*-eQTL (gene expression) and *cis*-sQTL (gene splicing) summary statistics from BrainMeta v.2, derived from RNA-seq data of 2,865 brain cortex samples from 2,443 individuals of European ancestry^48^. Our GWAS variants were mapped to 16,375 eQTL and 58,941 sQTL probes. We retained results with an FDR < 0.05, *P*_HEIDI_ > 0.01 and those mapping to genome-wide-significant GWAS loci. Significant SMR hits were assigned to index variants using PLINK clumping (window size = 3,000 kb; *R*^2^ > 0.80). Genes implicated by eQTL and sQTL SMR were nominated separately and ranked using the SMR *P* value.

### Gene nomination through GTEx eQTL lookup

Index variants and their genome-wide significant neighbors in strong LD (*R*^2^ > 0.8) were mapped to *cis*-QTLs from the GTEx v.8 database^46^. Single-tissue QTLs were retrieved from the prefiltered file (GTEx_Analysis_v8_eQTL.tar), covering 49 tissues. Multi-tissue QTLs were obtained from the METASOFT results (GTEx_Analysis_v8.metasoft.txt.gz), retaining variant–gene associations available in 10 or more tissues and with an *m* value equal to or greater than 0.9 (that is, the posterior probability that the effect exists) in 50% or more of tissues. To ensure robustness, only associations with meta-analytical *P* < 5.0 × 10^−8^ (Han and Eskin’s random-effects (RE2) model) were considered, yielding 4,420,841 multi-tissue QTLs. Variant mapping was done using the GTEx hg19 liftover variant IDs. If multiple variants implicated the same gene within a locus, we reported the variant in strongest LD with the index variant. Ensembl gene IDs were converted to HUGO Gene Nomenclature Committee symbols using biomaRt^118^. Genes implicated by single-tissue and multi-tissue QTLs were nominated separately for prioritization and ranked according to the number of significant tissue associations.

### Gene nomination through polygenic priority scores

We used PoPS v.0.2 (ref. ^49^) to identify likely causal genes within significant GWAS loci. PoPS builds on MAGMA^119^ gene-level associations and leverages subthreshold polygenic signals, integrating more than 57,000 features from sources such as single-cell RNA-seq datasets, curated biological pathways and protein–protein interaction networks. We used the same PoPS feature map and MAGMA gene annotation file as in the original publication (www.finucanelab.org/data)^49^. MAGMA v.1.10 was applied to our GWAS summary statistics using SNP-wise mean gene analysis, with LD data from 53,057 individuals of European ancestry (combined discovery and UKB replication sample). For each index variant identified through the conditional analyses, up to 3 genes within 500 kb with the highest PoPS scores were nominated for gene prioritization.

### Gene prioritization

Genes were prioritized based on seven evidence streams: (1) ANNOVAR functional annotation of credible variants, summing the posterior probabilities per gene; (2) transcript consequences of nonsynonymous exonic variants, ranked according to CADD score; (3) SMR eQTLs ranked according to *P* value; (4) sQTLs ranked according to *P* value; (5) GTEx single-tissue eQTLs; and (6) multi-tissue eQTLs, ranked according to the number of significant associations across tissues; and (7) PoPS ranked according to prioritization score. A composite priority score was calculated for each nominated gene as described below.

Let *i* denote a gene and *j* denote the index of the nomination category. The priority score (*P*_*i*_) for gene *i* combines the cumulative posterior probability (*C*_*i*_) from variant annotations and the gene’s rank (*R*_*ij*_) across six additional evidence categories (${{j}}\in \left[1,6\right]$) as

$${{{P}}}_{{{i}}}={{{C}}}_{{{i}}}+\mathop{\sum }\limits_{{{j}}=1}^{6}\frac{2\left({{{n}}}_{{{j}}}+1-{{{R}}}_{{{ij}}}\right)}{{{{n}}}_{{{j}}}\left({{n}}_{{j}}+1\right)}$$

where *P*_*i*_ denotes the priority score for gene *i*, *C*_*i*_ denotes the cumulative posterior probability of variants mapped to gene *i*, *n*_*j*_ denotes the number of genes ranked in nomination category *j* and *R*_*ij*_ denotes the rank of gene *i* in nomination category *j*.This formulation assigns greater weight to top-ranked genes and ensures that each category contributes equally (one point per category). The gene with the highest *P*_*i*_ per locus was designated the prioritized gene.

### GWAS Catalog search

We queried the National Human Genome Research Institute GWAS Catalog (13 September 2024 release; gwas_catalog_v1.0-associations_e112_r2024-09-13.tsv)^52^ for all index variants identified using conditional analysis and their genome-wide significant neighbors in strong LD (3,000-kb window, *R*^2^ > 0.8). Neighboring variants were identified through *P*-value-informed clumping in PLINK v.1.90b6.8 (ref. ^105^). Only GWAS Catalog entries reaching genome-wide significance were retained.

### Gene-based analysis

We performed gene-based analyses using fastBAT in GCTA v.1.93.1f^70^. Gene coordinates were obtained from the RefSeq GFF3 annotation file (GRCh37.p13; release 105.20201022)^120^. NCBI chromosome names were converted to UCSC format. We selected protein-coding genes located on chromosomes 1–22, X and Y, removing duplicates gene names, by keeping the first entry sorted according to chromosome, symbol and coordinates. This yielded 19,299 genes, of which 18,632 were successfully mapped to GWAS variants. Analyses used linkage data from the combined UKB discovery and replication sample (*n* = 53,057, European ancestry), applying no flanking window to reduce gene-level dependency. Genes with an FDR < 0.05 were considered significant. To identify independent associations, we applied *P*-value-informed clumping with a 3,000-kb window size. Distinct associations across the 3 BAG traits were determined with second-level clumping, using each gene’s top *P* value, again with a 3,000-kb window size.

### Pathway analyses

We conducted GO pathway analyses using the R package GOfuncR v.1.14.0, based on the GO.db v.3.14.0 and *Homo*.*sapiens* v.1.3.1 annotations^71,121,122^. GO provides a curated framework to categorize genes based their molecular function, cellular components where they perform actions and the higher-order biological processes they contribute to. Gene set enrichment analyses were performed on the full fastBAT gene-based results, testing for lower-than-expected *P* value ranks using the Wilcoxon rank-sum test. By default, GOfuncR calculates family-wise error rates (FWERs) in each of the three GO aspects using random permutations. To reduce false discoveries, we joined these permutation-based results to calculate FWERs across the three GO aspects. We further refined significant results (FWER < 0.05) by applying the elim algorithm to decorrelate overlapping terms and retain the most specific^123^. For interpretation, we also determined the number of distinct loci contributing to each enriched term, applying 3,000 kb clumping to account for spatial gene clustering.

### PGS analysis

To estimate the variance in BAG explained by PGS, we used a conventional clumping and *P* thresholding (C + P) approach implemented in PRSice-2 v.2.3.3 (ref. ^124^) along with two Bayesian polygenic prediction methods, SBayesR and SBayesRC, implemented in GCTB v.2.5.2 (ref. ^40^). For the C + P approach, we used *R*^2^ > 0.1, a 500-kb window size and 10 predefined *P* value thresholds^79,112^. Unlike the C + P approach, SBayesR and SBayesRC jointly model all variant effects, with SBayesRC additionally incorporating functional annotations. SBayesR/RC were used with eigendecomposed LD matrices (~7 M variants), and stratified LDSC baseline-LD v.2.2 annotations. Missing variants were imputed. Default settings were used (--gamma 0,0.001,0.01,0.1,1 --pi 0.99,0.005,0.003,0.001,0.001 --chain-length 3,000 --burn-in 1,000). The resulting weights were applied to calculate the PGS in target samples using PLINK (--score)^105^.

PGS weights derived from the discovery sample (*n* = 32,634) were tested in the European-ancestry replication sample (*n* = 20,423). To estimate PGS performance in the combined discovery and replication sample, we reran the GWAS meta-analysis excluding 2,000 individuals of European ancestry held out as a target set (total training *n* = 52,890). Transferability was assessed in AFR (*n* = 337), CSA (*n* = 638) and EAS (*n* = 291) UKB subsamples. Associations between PGS and BAG were evaluated using partial product-moment correlations, adjusting for sex, age, age^2^, scanner site, total intracranial volume, genotyping array and 20 genetic principal components (4 for non-European samples).

### Genetic correlations

We used bivariate LDSC (v.1.0.1)^36^ to compute pairwise genetic correlations among BAG traits and between BAG and other complex traits. These included 38 commonly studied traits spanning the mental and physical health domains^77–79^ (Supplementary Table 28), as well as 989 heritable UKB traits with publicly available GWAS summary statistics^80^. Analyses were restricted to HapMap3 variants, excluding the MHC region. Genetic correlations with an FDR < 0.05 were considered significant.

### Mendelian randomization

Potential causal associations were examined using GSMR implemented in GCTA v.1.93.1f^81^. GSMR uses multiple genetic variants (here clumped with an *R*^2^ < 0.001 and a 10,000-kb window) as instruments (z) to test for causality between an exposure (x) and outcome variable (y), using the ratio *β*_xy_ = *β*_zy_/*β*_zx_. Designed for two-sample scenarios, GSMR estimates the exposure–outcome effects using GWAS summary statistics from independent samples. Estimates from multiple instruments are integrated using generalized least squares. Instrument heterogeneity is assessed via HEIDI (*P* < 0.01), removing outliers deviating from the causal model. To facilitate effect-size comparisons, we standardized instrument effects on continuous exposures (*β*_zx_) based on *z*-statistic, allele frequency and sample size. GSMR has been demonstrated with superior power to inverse-variance-weighted MR and MR Egger regression^81^. We used GSMR as the primary method for inferring causality and conducted sensitivity analyses using nine alternative MR approaches: inverse-variance-weighted MR (simple, debiased and penalized); MR Egger regression; weighted median-base; maximum-likelihood; mode-based; MR lasso; and contamination-mixture MR, implemented in MendelianRandomization v.0.10.0 (ref. ^125^). These methods used the same variants as GSMR but without HEIDI-based outlier removal. Twelve risk factors were selected based on the availability of large-scale GWAS, not including UKB individuals^79,81^. Both forward and reverse MR were performed to assess any potential bidirectional effects between risk factors and BAG.

### Polygenicity

We used GENESIS v.1.0 (commit e4e6894) to infer genetic effect-size distributions and estimate the number of susceptibility variants underlying BAG under a normal-mixture model of variant effects^84^. Analyses included 1.07 million HapMap3 variants with MAF > 0.05, excluding the MHC region, SNPs with a sample size of less than 0.67 × 90th percentile and those with extreme effect sizes (*z*^2^ > 80). We fitted the GENESIS three-component model, which assumes that 99% of variant effects are null, while the remaining 1% follow a mixture of 2 normal distributions, allowing a subset of susceptibility variants to exhibit larger effects. We chose the three-component model over the simpler two-component model because it provides better fits across diverse traits, is robust to model misspecification and reduces downward bias in polygenicity estimates^84^. Default settings were used for defining tagging SNPs (*R*^2^ > 0.1 and 1,000-kb window). Neuroticism and height served as benchmark traits for comparison^86,87^.

### Reporting summary

Further information on research design is available in the Nature Portfolio Reporting Summary linked to this article.

## Supplementary information

Supplementary Information Supplementary Figs. 1–48 and Tables 1–34.

Reporting Summary

Supplementary Tables 1–34 Supplementary Tables 1–34.

## Data Availability

The individual-level data used in this study were obtained from the UKB and LIFE-Adult study. Access to these datasets is restricted to researchers with approved projects. The GWAS summary statistics and polygenic score weights generated from our analyses are available via Zenodo at 10.5281/zenodo.14826943 (ref. ^126^). Genetic correlation analyses involving UKB traits were conducted using the GWAS summary statistics provided in ref. ^80^ (10.5281/zenodo.7186871). Additional GWAS summary statistics used for genetic correlation and Mendelian randomization analyses are detailed in Supplementary Tables 28 and 31.

## Code availability

All scripts, conda environments and a complete list of software and resources used in this study are available via GitHub at https://github.com/pjawinski/ukb_brainage.
