# Supplementary material for: Genome-wide analysis of brain age identifies 59 associated loci and unveils relationships with mental and physical health
Source: Nat Aging. 2025 Oct 3;5(10):2086–103. doi: 10.1038/s43587-025-00962-7 (PMC12532595; doi:10.1038/s43587-025-00962-7)
Supplement: Supplementary file 1 — Supplementary Figs. 1–48 and Tables 1–34. [file 43587_2025_962_MOESM1_ESM.pdf]

nature aging

Supplementary information https://doi.org/10.1038/s43587-025-00962-7

Genome-wide analysis of brain age identifies 59 associated loci and unveils relationships with mental and physical health

In the format provided by the authors and unedited

Supplementary Figures

Phenotypic results

Fig. A1 Comparison of cross-trait associations in males and females 4
Fig. A2 QQ-plots of differential cross-trait associations in males and females 4
Fig. A3 Correlations with FreeSurfer brain structure variables 5
Fig. A4 Comparison of FreeSurfer correlations in males and females 6
Fig. A5 QQ-plots of differential FreeSurfer correlations in males and females 6

Discovery GWAS: Identification of 25 associated loci

Fig. A6 Manhattan and quantile-quantile-plots 8
Fig. A7 Regional plots 01-06 for grey matter brain age gap 9
Fig. A8 Regional plots 07-12 for grey matter brain age gap 10
Fig. A9 Regional plots 01-06 for white matter brain age gap 11
Fig. A10 Regional plots 07-12 for white matter brain age gap 12
Fig. A11 Regional plots 13-26 for white matter brain age gap 13
Fig. A12 Regional plots 01-06 for grey and white matter brain age gap 14
Fig. A13 Regional plots 07-12 for grey and white matter brain age gap 15
Fig. A14 Regional plots 13-12 for grey and white matter brain age gap 16
Fig. A15 Relationships between genetic principal components and brain age gap 17

Replication GWAS: Corroborating discovered loci

Fig. A16 Agreement between age predictions from single-model vs. multi-model approach and feature importance in XGBoost tree-based models 19
Fig. A17 Manhattan and quantile-quantile-plots for grey matter BAG 20
Fig. A18 Manhattan and quantile-quantile-plots for white matter BAG 21
Fig. A19 Manhattan and quantile-quantile-plots for combined BAG 22
Fig. A20 EUR-ancestry GWAS: Manhattan and quantile-quantile plots 23
Fig. A21 EUR-ancestry GWAS: QQ-plots of variants with discovery $p <$ 1e-6 23
Fig. A22 Multi-ancestry GWAS: Manhattan and quantile-quantile plots 24
Fig. A23 Multi-ancestry GWAS: QQ-plots of variants with discovery $p <$ 1e-6 24

GWAS across discovery and replication: Identification of 59 associated loci

Fig. A24 ANNOVAR enrichment test of functional consequences 26
Fig. A25 Regional plots 01-06 for grey matter brain age gap 27
Fig. A26 Regional plots 07-12 for grey matter brain age gap 28
Fig. A27 Regional plots 13-18 for grey matter brain age gap 29
Fig. A28 Regional plots 19-24 for grey matter brain age gap 30
Fig. A29 Regional plots 25-26 for grey matter brain age gap 31
Fig. A30 Regional plots 01-06 for white matter brain age gap 32
Fig. A31 Regional plots 07-12 for white matter brain age gap 33

Fig. A32	Regional plots 13-18 for white matter brain age gap	34
Fig. A33	Regional plots 19-24 for white matter brain age gap	35
Fig. A34	Regional plots 25-30 for white matter brain age gap	36
Fig. A35	Regional plots 31-34 for white matter brain age gap	37
Fig. A36	Regional plots 01-06 for combined brain age gap	38
Fig. A37	Regional plots 07-12 for combined brain age gap	39
Fig. A38	Regional plots 13-18 for combined brain age gap	40
Fig. A39	Regional plots 19-24 for combined brain age gap	41
Fig. A40	Regional plots 25-30 for combined brain age gap	42
Fig. A41	Regional plots 31-36 for combined brain age gap	43
Fig. A42	Regional plots 37-39 for combined brain age gap	44
Fig. A43	Mendelian Randomization results for grey matter BAG (outcome)	45
Fig. A44	Mendelian Randomization results for grey matter BAG (exposure)	46
Fig. A45	Mendelian Randomization results for white matter BAG (outcome)	47
Fig. A46	Mendelian Randomization results for white matter BAG (exposure)	48
Fig. A47	Mendelian Randomization results for combined matter BAG (outcome)	49
Fig. A48	Mendelian Randomization results for combined matter BAG (exposure)	50

Phenotypic results

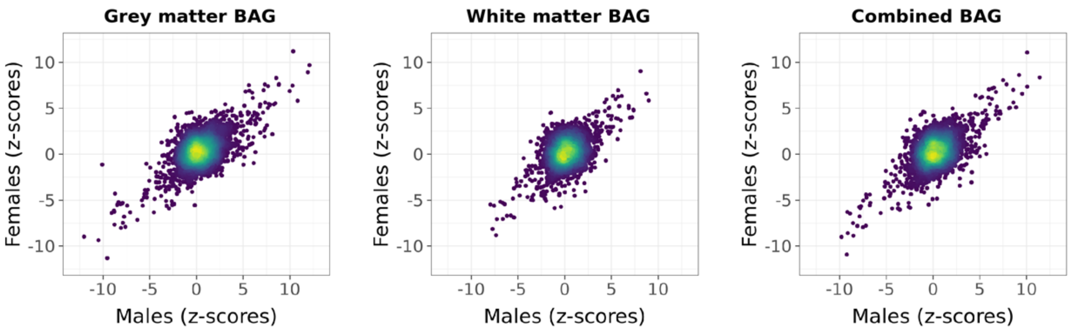

Fig. A1 Comparison of the PHESANT cross-trait associations in male and female individuals from the discovery sample. The x- and y-axes represent z-scores, derived from transforming observed PHESANT p-values to a standard normal distribution, with the direction indicated by regression beta weights. A total of 4,031 associations were analyzed in up to $n = 15{,}549$ males and $n = 17{,}084$ females. The plots indicate an overall positive linear relationship between male and female association results, suggesting that phenotypes with strong evidence of association with brain age gap (BAG) in males tend to show similarly strong evidence of association with BAG in females.

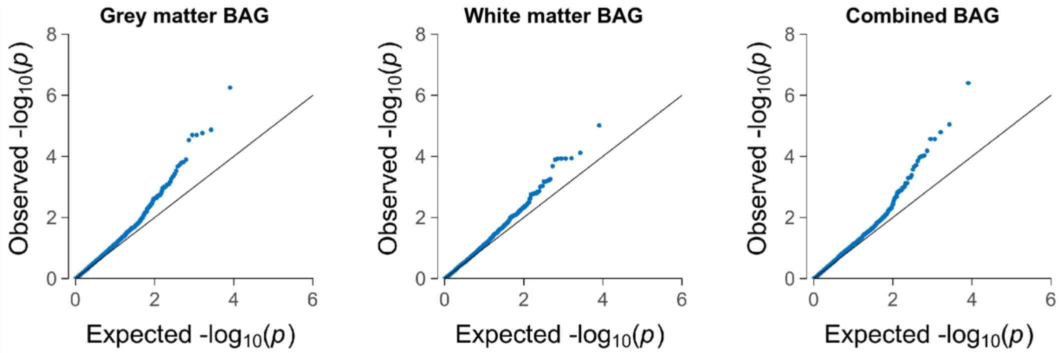

Fig. A2 Quantile-quantile (qq) plots of differential cross-trait associations in male and female individuals in the discovery sample. The QQ plots compare observed p-values from the differential association analysis to expected p-values under the null hypothesis of no effect, plotted on a -log10 scale. The differential effects were calculated as the difference between the PHESANT beta coefficients in males (β_m) and females (β_f), divided by the square root of the sum of the squared standard errors of the beta coefficients (SE_m and SE_f), as follows: $z = (\beta_m - \beta_f)/sqrt(SE_m^2 + SE_f^2)$. The resulting z-values were converted into p-values using standard normal probabilities. A total of 4,031 associations were analyzed, using data from up to $n = 15{,}549$ males and $n = 17{,}084$ females. The qq-plots show a trend towards left-deflation from the solid diagonal, indicating stronger evidence of differential effects between males and females than expected under the null hypothesis. Please note that due to high statistical power, significant results may not indicate large differences between the sexes.

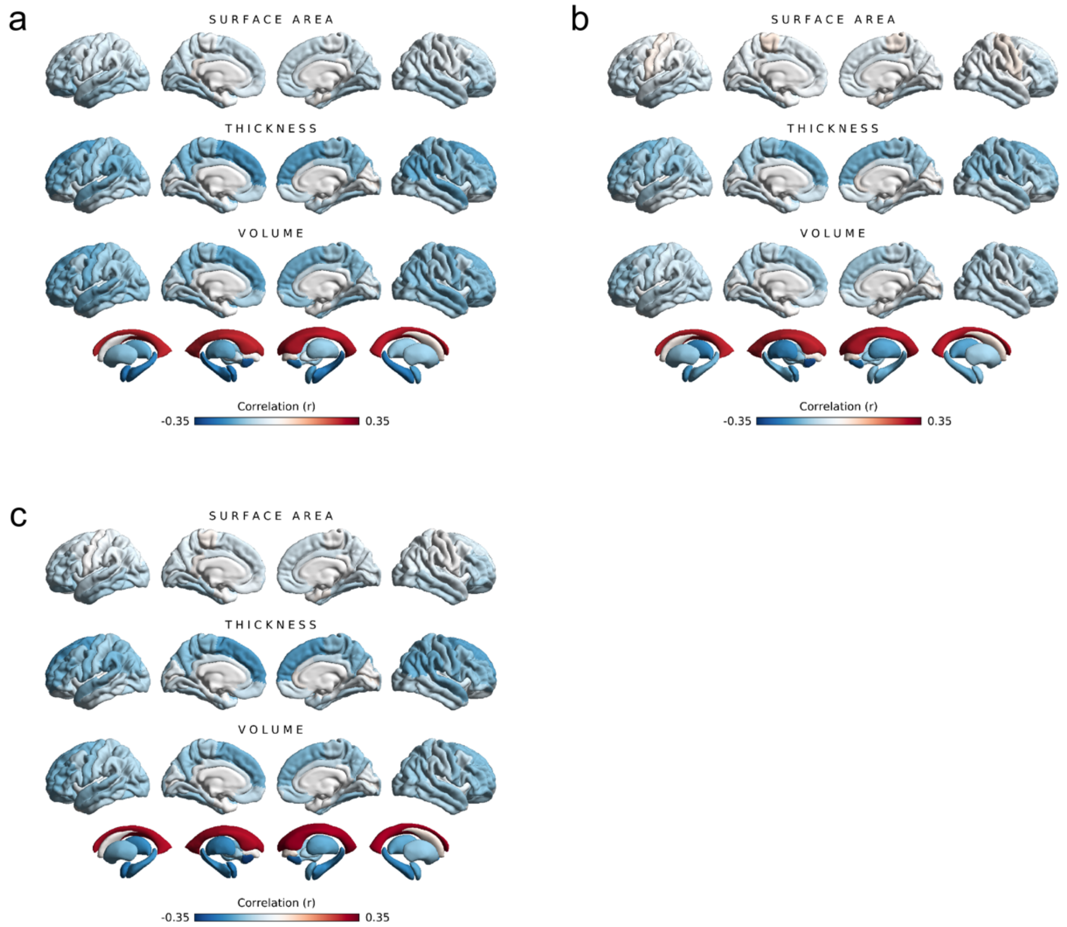

Fig. A3 Surface plots showing the correlations between brain age gap and 220 FreeSurfer brain structure variables. Analyses are based on $n = 32{,}278$ individuals from the discovery sample. Brain structure variables were obtained from the FreeSurfer aparc and aseg output (surface area, cortical thickness, and cortical volume of 34 bilateral cortical segmentations, as well as volume of 16 bilateral subcortical segmentations). Colors reflect the strength and direction of partial product-moment correlations (adjusted for sex, age, age^2, scanner site, total intracranial volume). Plots have been created using the ENIGMA toolbox in MATLAB. **(a)** grey matter brain age gap, **(b)** white matter brain age gap, **(c)** combined grey and white matter brain age gap

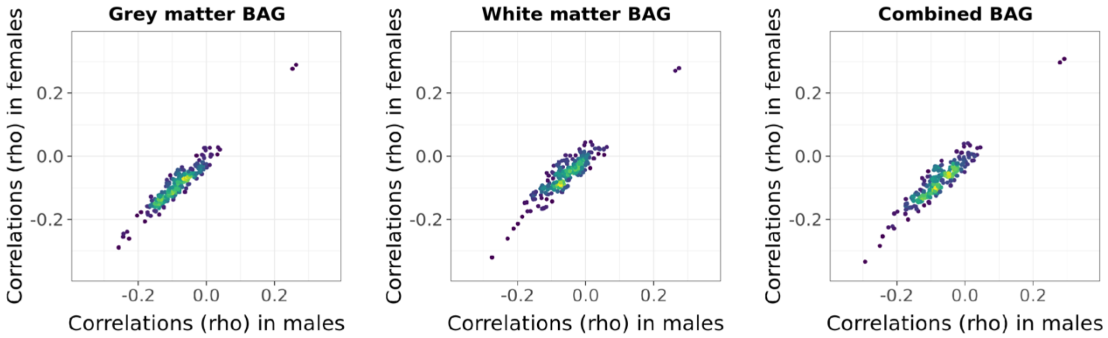

Fig. A4 Comparison of FreeSurfer associations in male and female individuals from the discovery sample. The x- and y-axes represent product-moment correlation coefficients (ρ) between brain age gap (BAG) and 220 FreeSurfer cortical and subcortical brain structure measures. Correlations were corrected for age, age^2, total intracranial volume, and scanner site. The results are based on $n = 15{,}317$ male and $n = 16{,}961$ female individuals. The scatterplot demonstrates a strong positive linear relationship between male and female correlation coefficients, indicating that the associations between BAG and regional brain measures are relatively consistent across sexes.

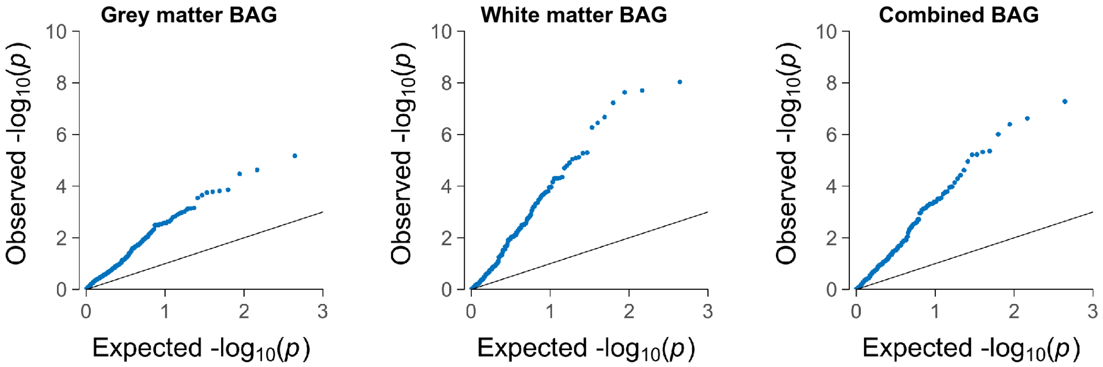

Fig. A5 Quantile-quantile (qq) plots of differential FreeSurfer associations in male and female individuals from the discovery sample. The QQ plots compare observed p-values from the differential association analysis to expected p-values under the null hypothesis of no effect, plotted on a -log10 scale. Differential effects were assessed by comparing the product-moment correlation coefficients in males and females using Fisher's r-to-z transformation and significance testing implemented in R package 'cocor'. A total of 220 associations between brain age gap (BAG) and FreeSurfer cortical and subcortical measures were analyzed, using data from $n = 15{,}317$ male and $n = 16{,}961$. The qq-plots exhibit a marked left-deflation from the solid diagonal, indicating that a substantial proportion of BAG-FreeSurfer associations significantly differ between males and females. Please note that due to high statistical power, significant results may not indicate large differences in correlations between males and females.

Discovery GWAS:
Identification of 25 associated loci

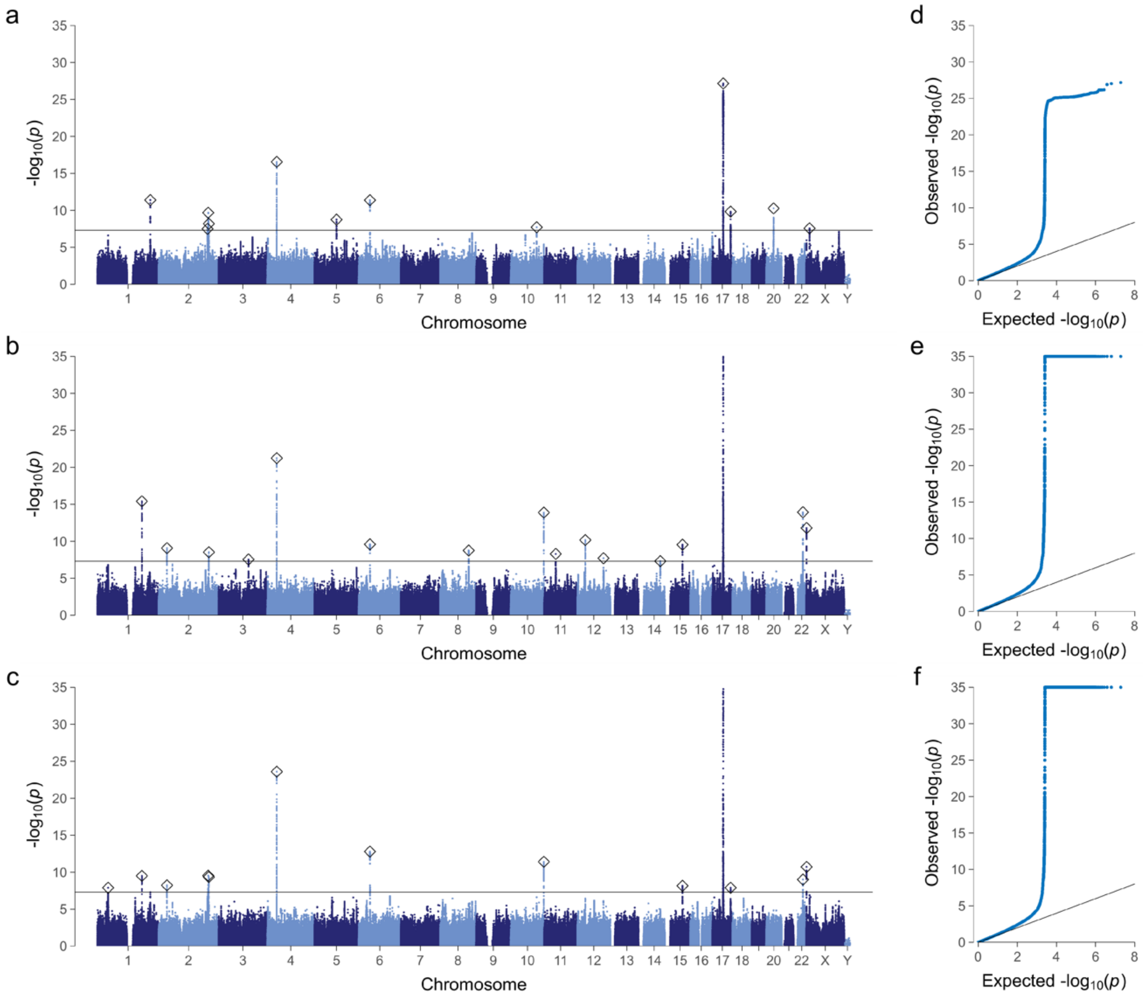

Fig. A6 Manhattan and quantile-quantile plots showing the results of the discovery genome-wide association analyses for the three brain age gap traits in $n = 32{,}634$ white-British UK Biobank individuals. Manhattan plots (**a-c**) show the p-values ($-\log_{10}$ scale) of the tested genetic variations on the y-axis and base-pair positions along the chromosomes on the x-axis. The solid horizontal line indicates the threshold of genome-wide significance ($p = 5.0\text{e-}08$). Index variations of identified loci are highlighted by diamonds. Results of pseudoautosomal variations have been added to chromosome 'X'. Quantile-quantile plots (**d-f**) show the observed p-values from the association analysis vs. the expected p-values under the null hypothesis of no effect ($-\log_{10}$ scale). For illustrative reasons, the y-axis has been truncated at $p = 1.0\text{e-}35$. **a,d** grey matter brain age gap; **b,e** white matter brain age gap; **c,f** combined grey and white matter brain age gap

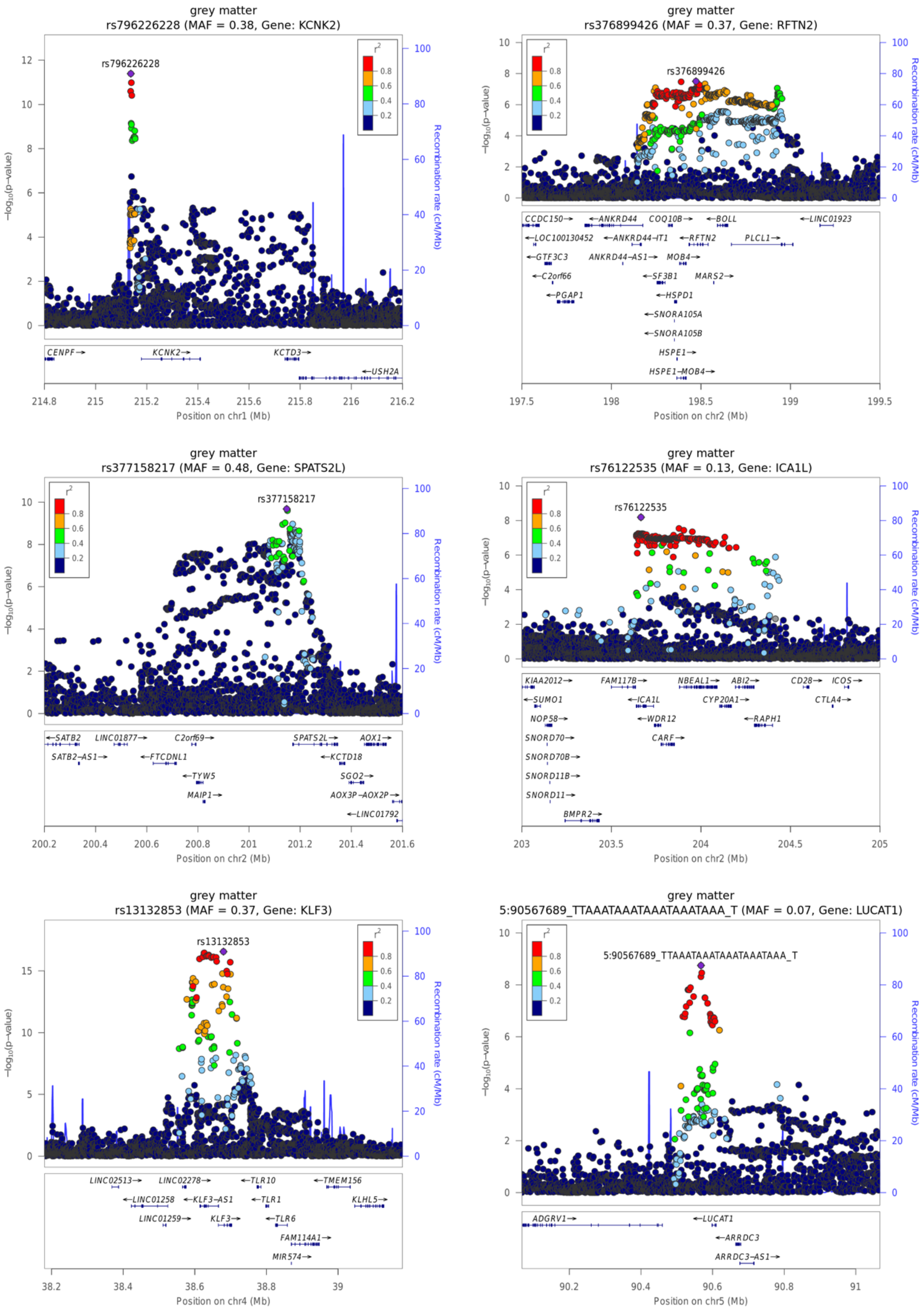

Fig. A7 Regional association plots for index variations 1-6 from the discovery genome-wide association analysis of grey matter brain age gap in $n = 32{,}634$ white-British ancestry individuals. Regional association plots were created using Locuszoom Standalone v1.4. SNP positions (dbSNP build 151) and refFlat gene locations (2020-08-17) are based on human genome build hg19 and were accessed via UCSC Genome Browser. Recombination rates were derived from HapMap phase II build GRCh37 (2011-01-19). MAF: Minor allele frequency, r^2: linkage disequilibrium between index variation and other variation in locus.

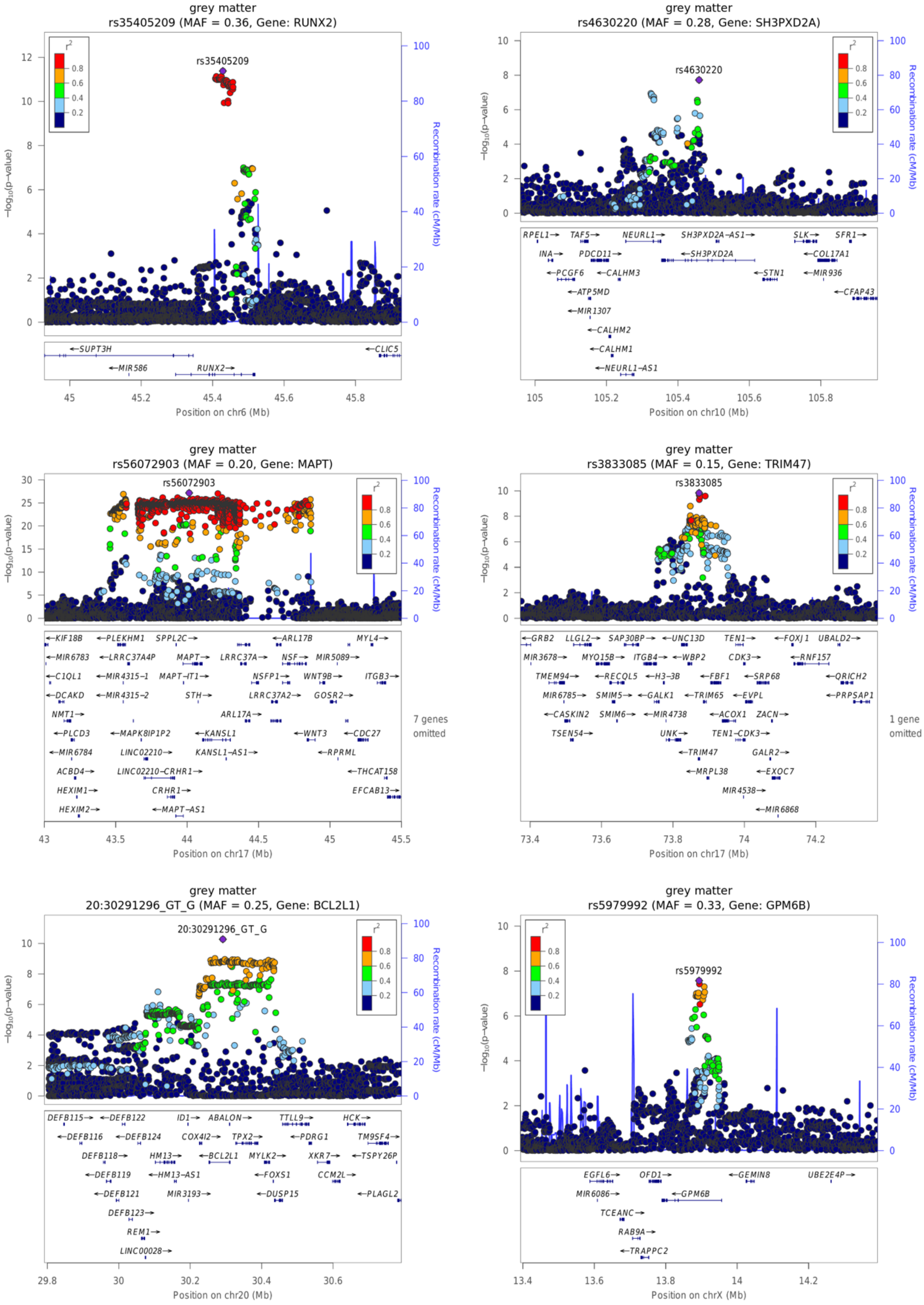

Fig. A8 Regional association plots for index variations 7-12 from the discovery genome-wide association analysis of grey matter brain age gap in $n = 32{,}634$ white-British ancestry individuals. Regional association plots were created using Locuszoom Standalone v1.4. SNP positions (dbSNP build 151) and refFlat gene locations (2020-08-17) are based on human genome build hg19 and were accessed via UCSC Genome Browser. Recombination rates were derived from HapMap phase II build GRCh37 (2011-01-19). MAF: Minor allele frequency, r^2: linkage disequilibrium between index variation and other variation in locus.

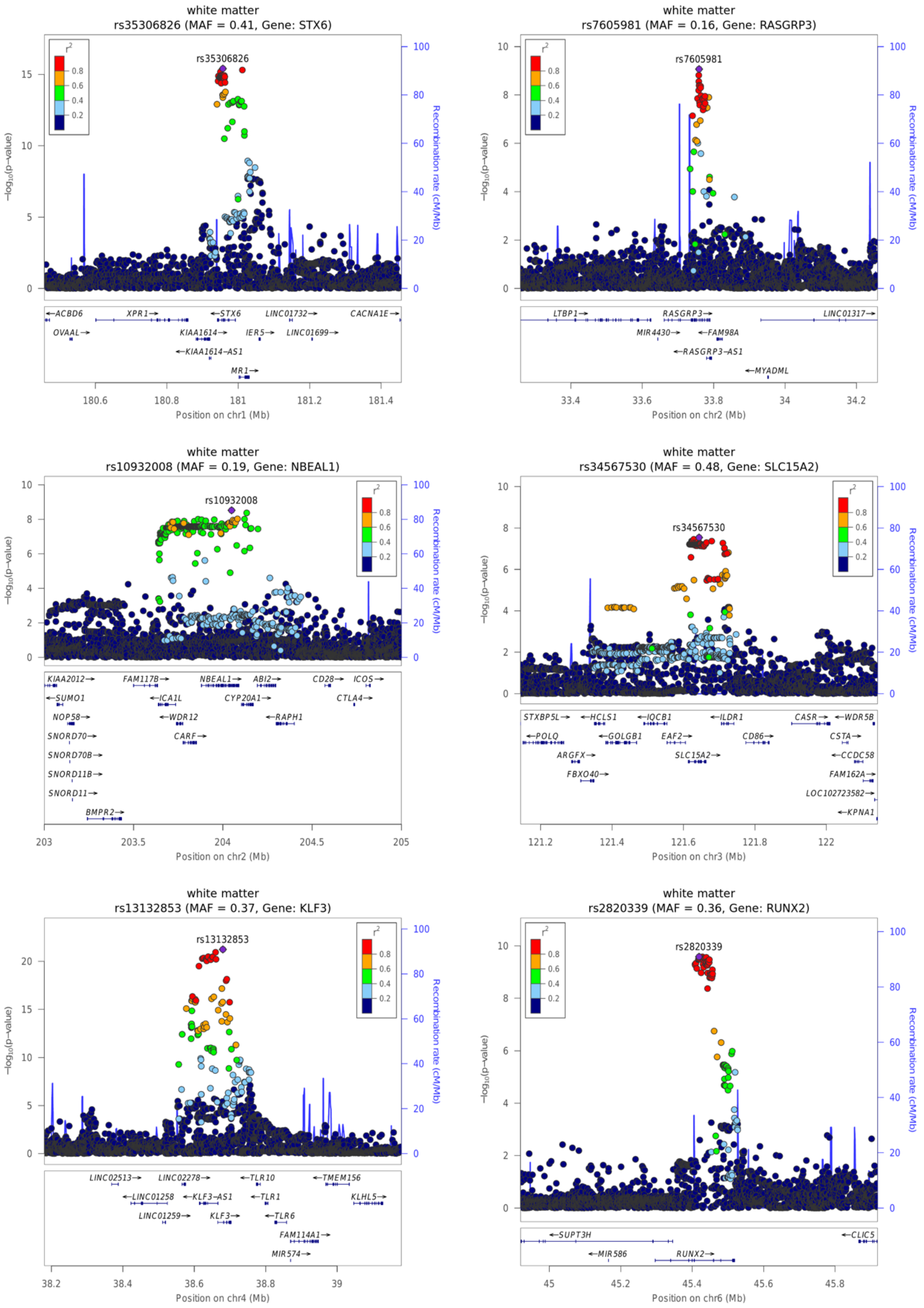

Fig. A9 Regional association plots for index variations 1-6 from the discovery genome-wide association analysis of white matter brain age gap in $n = 32{,}634$ white-British ancestry individuals. Regional association plots were created using Locuszoom Standalone v1.4. SNP positions (dbSNP build 151) and refFlat gene locations (2020-08-17) are based on human genome build hg19 and were accessed via UCSC Genome Browser. Recombination rates were derived from HapMap phase II build GRCh37 (2011-01-19). MAF: Minor allele frequency, r^2: linkage disequilibrium between index variation and other variation in locus.

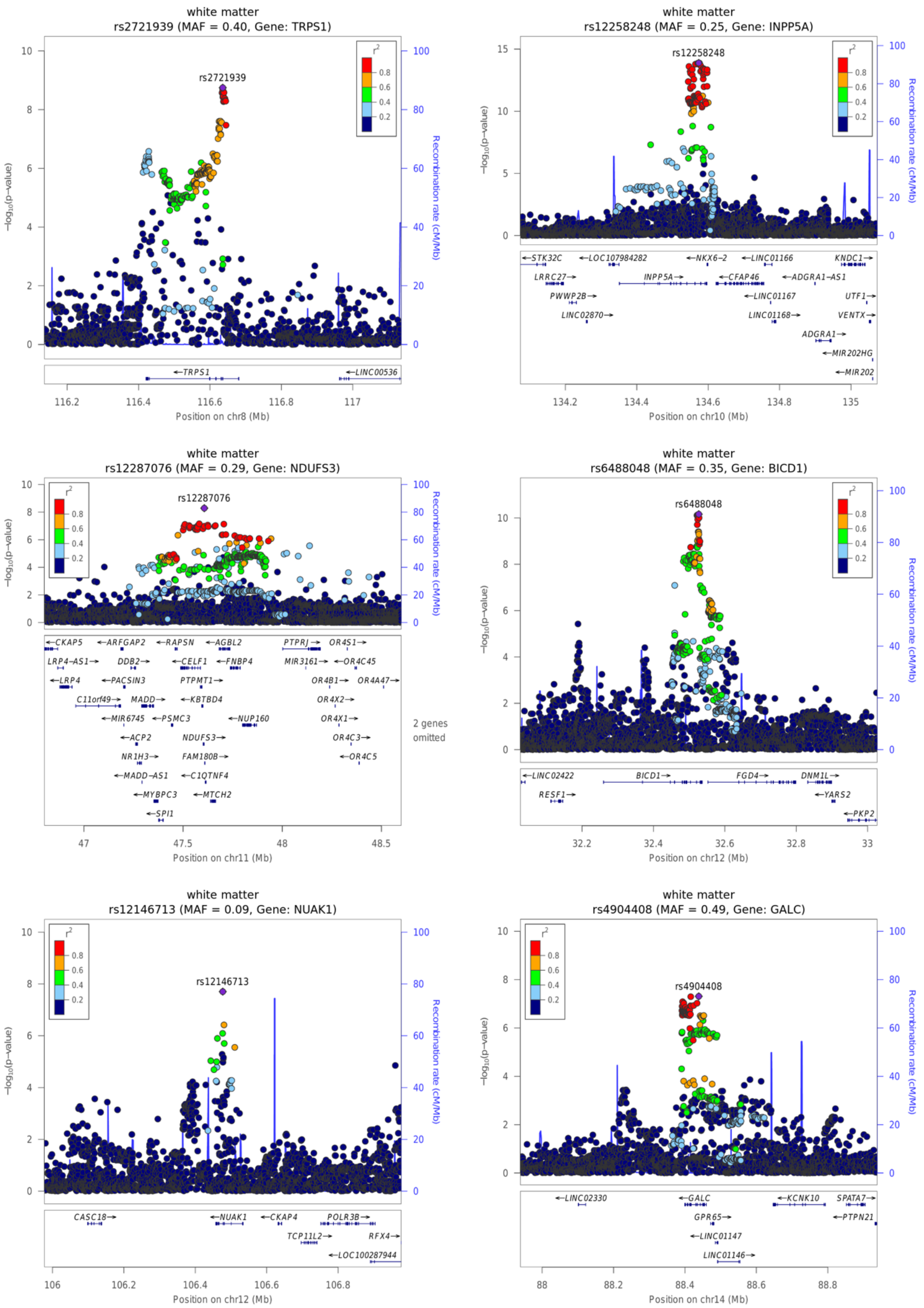

Fig. A10 Regional association plots for index variations 7-12 from the discovery genome-wide association analysis of white matter brain age gap in $n = 32{,}634$ white-British ancestry individuals. Regional association plots were created using Locuszoom Standalone v1.4. SNP positions (dbSNP build 151) and refFlat gene locations (2020-08-17) are based on human genome build hg19 and were accessed via UCSC Genome Browser. Recombination rates were derived from HapMap phase II build GRCh37 (2011-01-19). MAF: Minor allele frequency, r^2: linkage disequilibrium between index variation and other variation in locus.

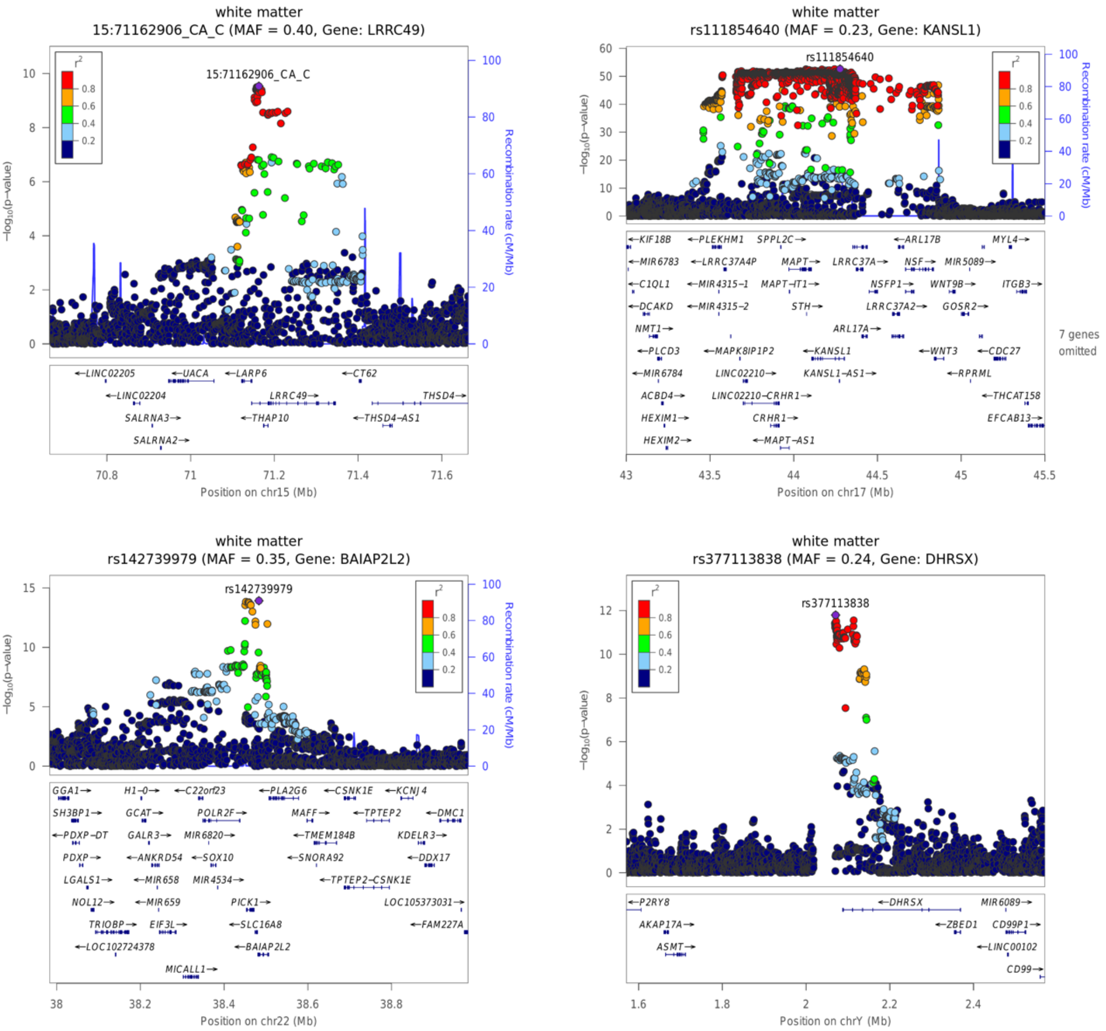

Fig. A11 Regional association plots for index variations 13-16 from the discovery genome-wide association analysis of white matter brain age gap in n = 32,634 white-British ancestry individuals. Regional association plots were created using Locuszoom Standalone v1.4. SNP positions (dbSNP build 151) and refFlat gene locations (2020-08-17) are based on human genome build hg19 and were accessed via UCSC Genome Browser. Recombination rates were derived from HapMap phase II build GRCh37 (2011-01-19). MAF: Minor allele frequency, r^2: linkage disequilibrium between index variation and other variation in locus.

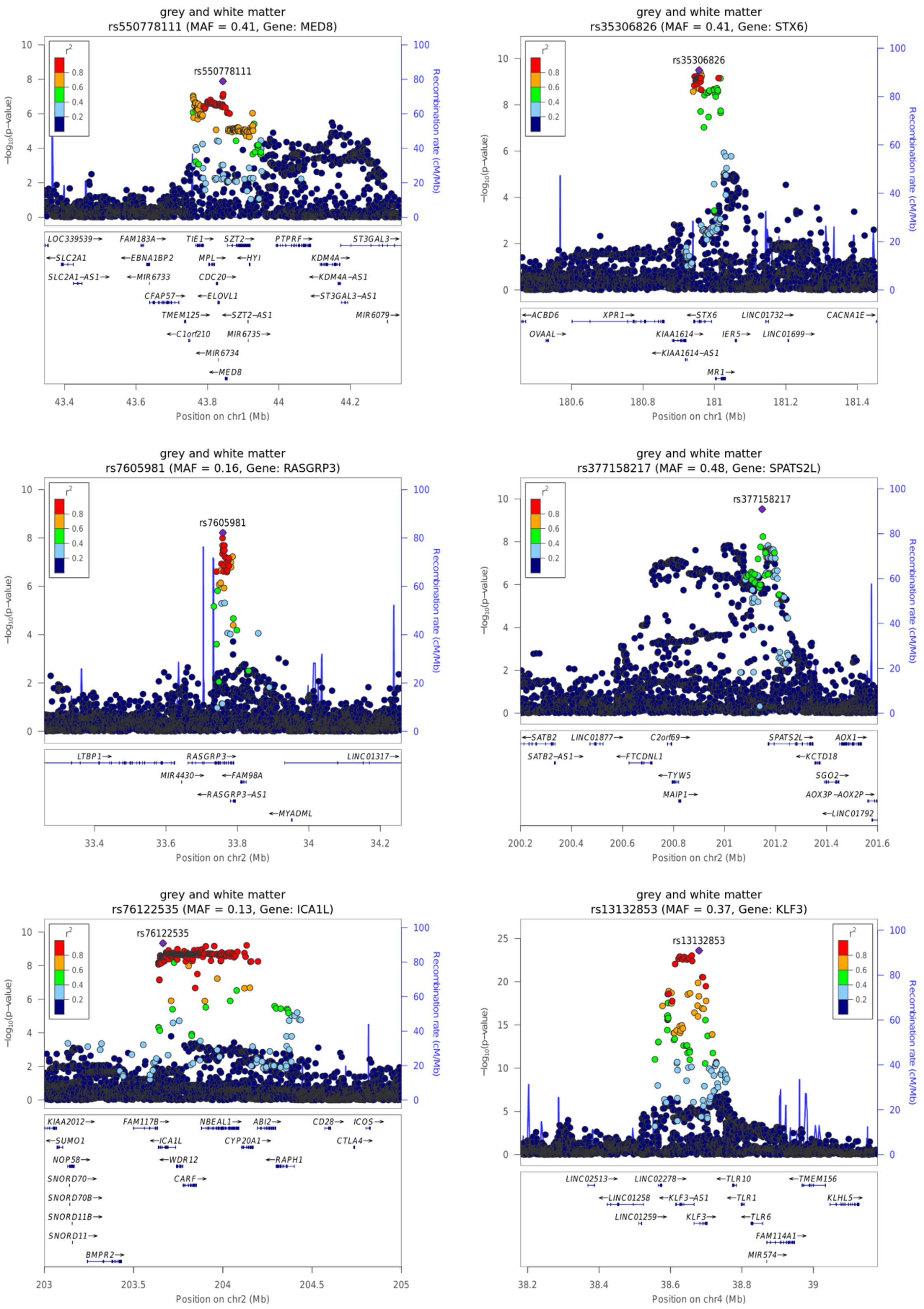

Fig. A12 Regional association plots for index variations 1-6 from the discovery genome-wide association analysis of combined grey and white matter brain age gap in $n = 32{,}634$ white-British ancestry individuals. Regional association plots were created using Locuszoom Standalone v1.4. SNP positions (dbSNP build 151) and refFlat gene locations (2020-08-17) are based on human genome build hg19 and were accessed via UCSC Genome Browser. Recombination rates were derived from HapMap phase II build GRCh37 (2011-01-19). MAF: Minor allele frequency, r^2: linkage disequilibrium between index variation and other variation in locus.

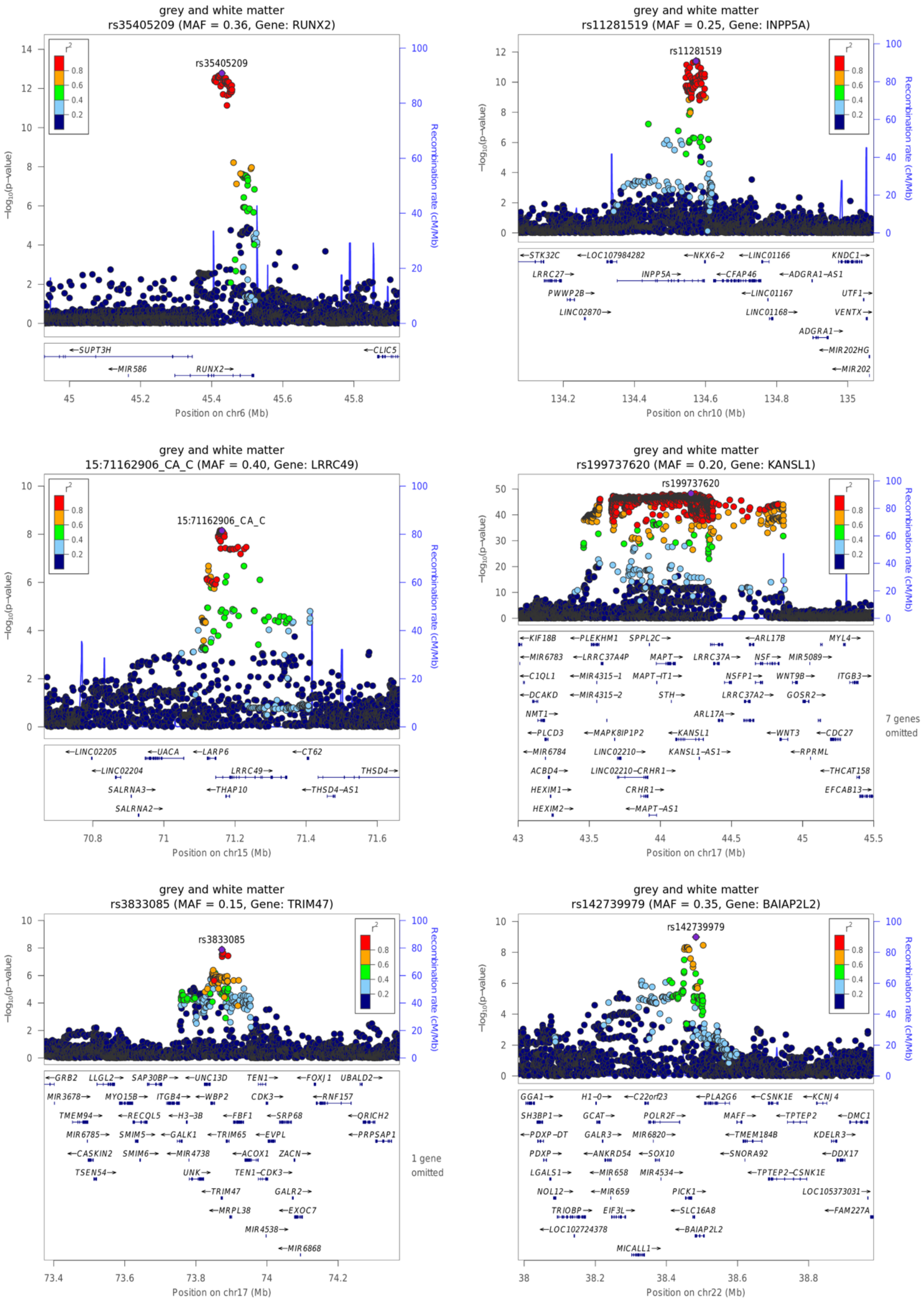

Fig. A13 Regional association plots for index variations 7-12 from the discovery genome-wide association analysis of combined grey and white matter brain age gap in $n = 32{,}634$ white-British ancestry individuals. Regional association plots were created using Locuszoom Standalone v1.4. SNP positions (dbSNP build 151) and refFlat gene locations (2020-08-17) are based on human genome build hg19 and were accessed via UCSC Genome Browser. Recombination rates were derived from HapMap phase II build GRCh37 (2011-01-19). MAF: Minor allele frequency, r^2: linkage disequilibrium between index variation and other variation in locus.

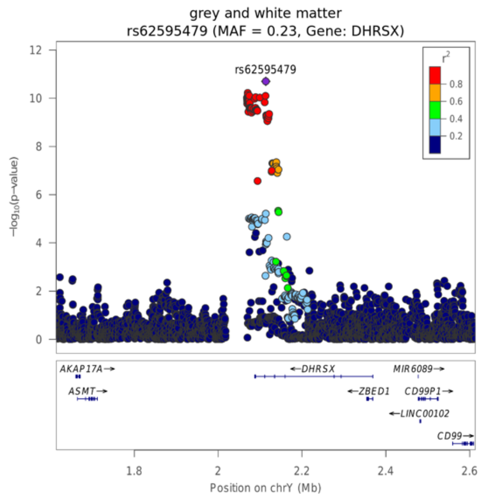

Fig. A14 Regional association plot for index variation 13 from the discovery genome-wide association analysis of combined grey and white matter brain age gap in $n = 32{,}634$ white-British ancestry individuals. Regional association plots were created using Locuszoom Standalone v1.4. SNP positions (dbSNP build 151) and refFlat gene locations (2020-08-17) are based on human genome build hg19 and were accessed via UCSC Genome Browser. Recombination rates were derived from HapMap phase II build GRCh37 (2011-01-19). MAF: Minor allele frequency, r^2: linkage disequilibrium between index variation and other variation in locus.

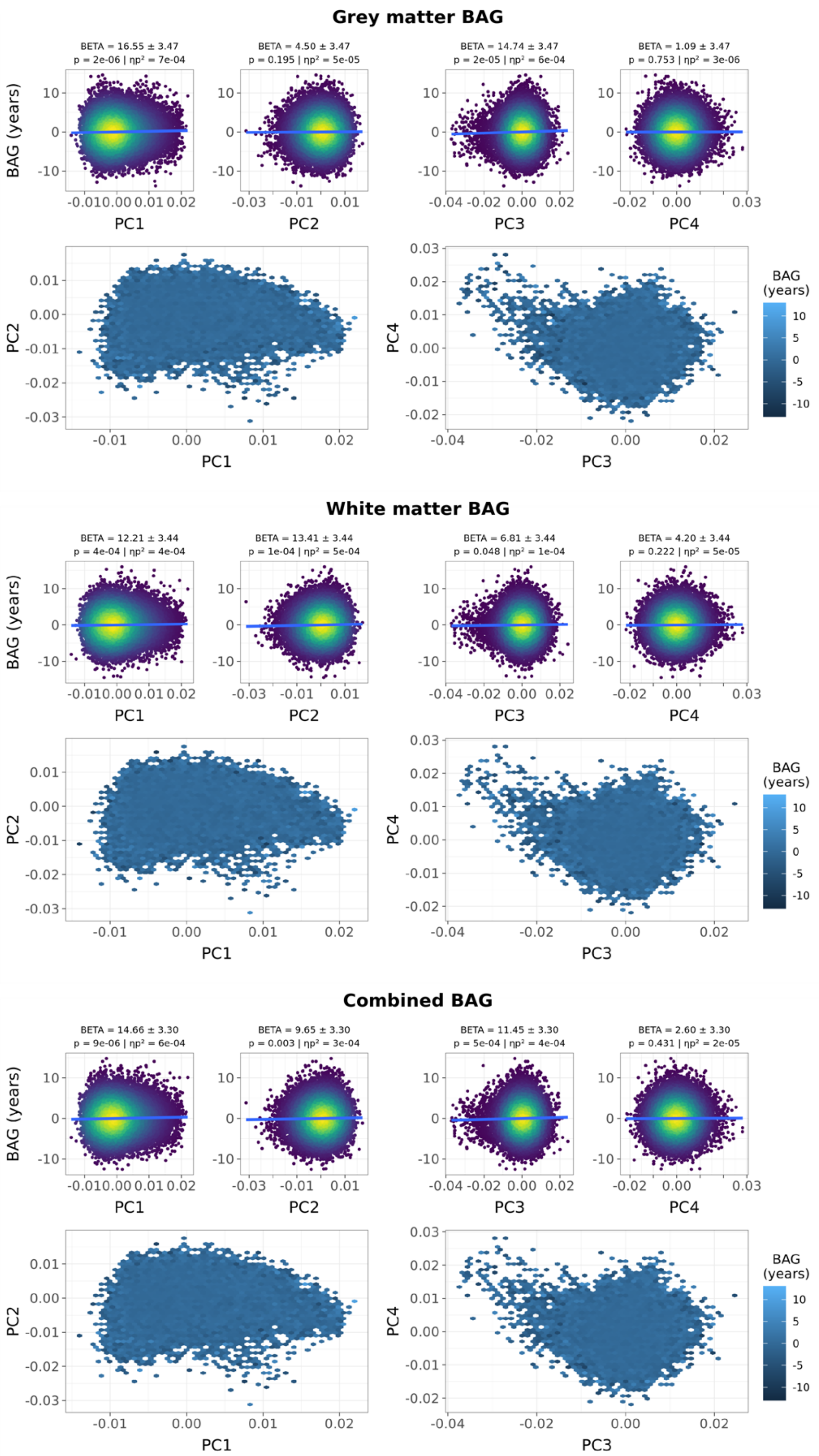

Fig. A15 Relationships between the four strongest genetic principal components (PC1-PC4) and brain age gap (BAG) in $n = 32{,}634$ individuals of the discovery sample. The top row shows point-density plots (scatterplots with color-coded local density) for PC1–PC4 vs. BAG across three BAG phenotypes (grey matter, white matter, combined), with a blue regression line indicating the linear model fit. Each plot includes regression statistics (BETA ± SE, p-value, partial η^2). The bottom row presents hexagonal bin plots, visualizing relationships between principal components, with color intensity reflecting BAG values. BAG and genetic principal components were residualized for sex, age, age^2, total intracranial volume, and genotyping array. Overall, results suggest that genetic principal components have only minor effects on BAG, with the strongest evidence observed for PC1 on grey matter BAG ($p = $ 2e-06, $\eta^2 = 0.0007$).

Replication GWAS:
Corroborating discovered loci

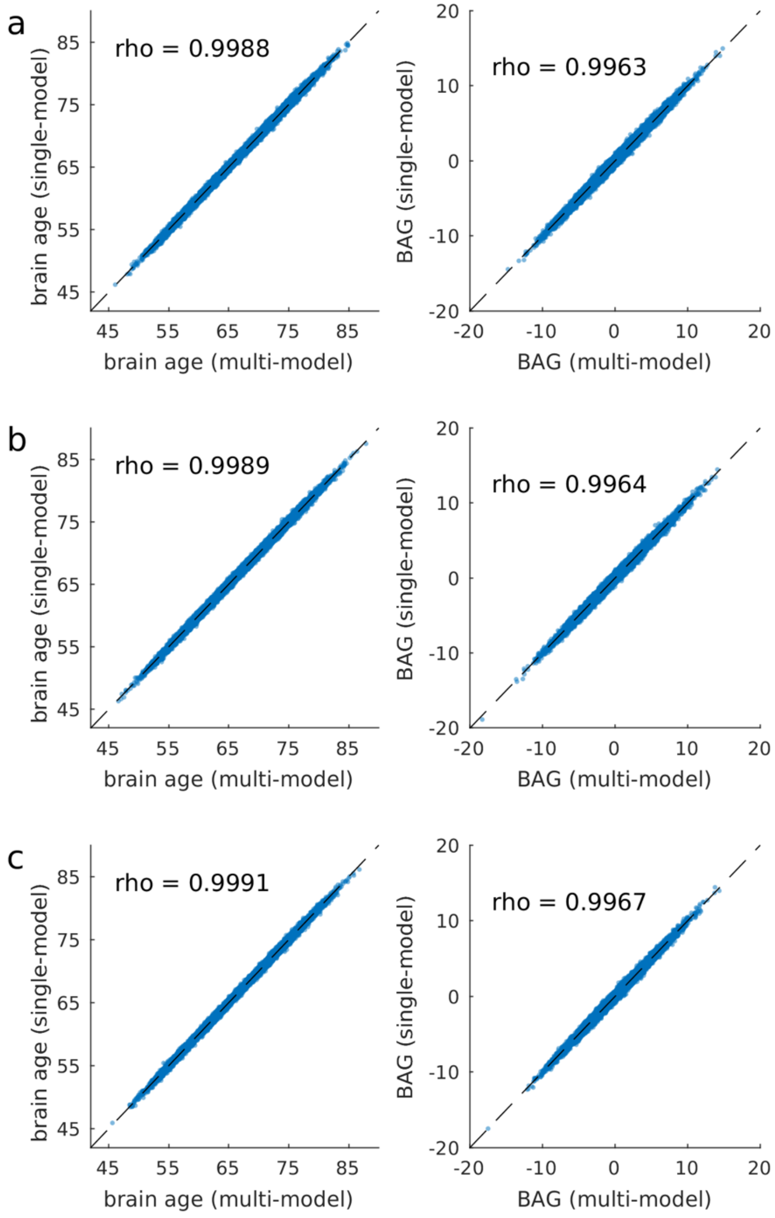

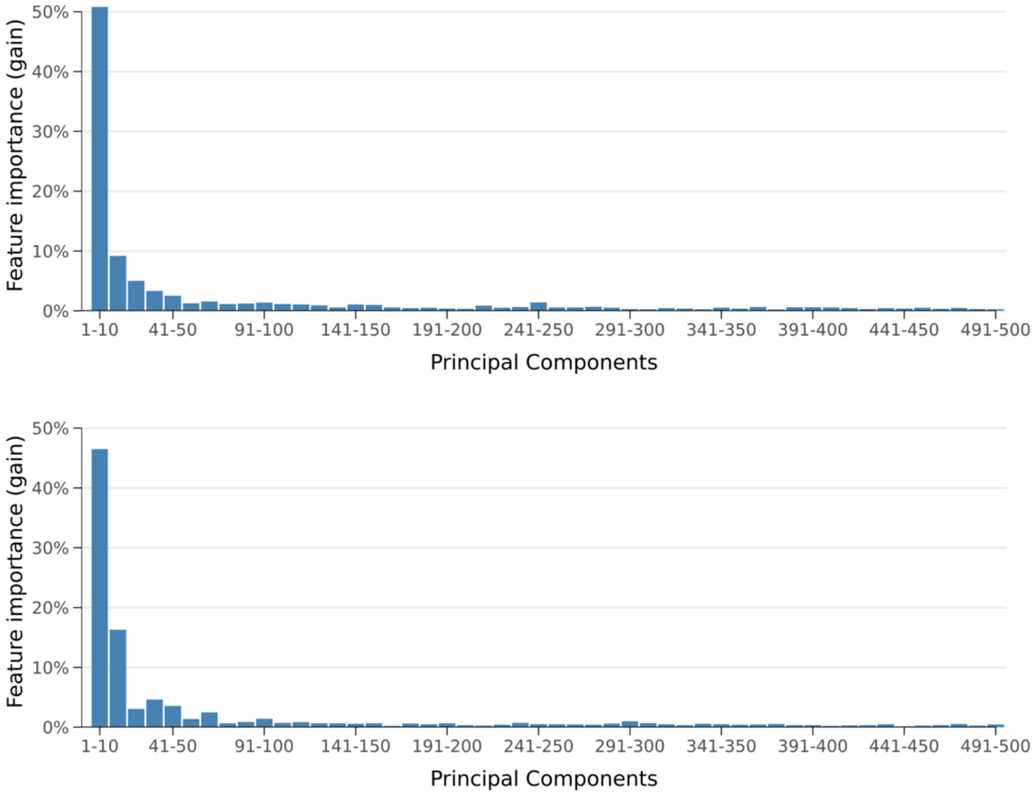

Fig. A16 Comparison of age predictions and brain age gap (BAG) estimates between two modeling approaches (single-model vs. multi-model) in the multi-ancestry replication sample of $n = 21{,}881$ UK Biobank participants. The single-model approach utilized a single ensemble model trained on the full discovery sample, whereas the multi-model approach combined results from 10-fold cross-validation with 100 repeats. From left to right: The first plot compares brain age estimates (i.e., age predictions) from both modeling approaches, demonstrating strong agreement. The second plot compares derived BAG estimates, residualized for sex, age, age^2, total intracranial volume, and scanner site, again showing high consistency. Due to this agreement and improved practicability, results from the single-model approach were chosen for subsequent genetic analyses. The third plot shows feature importance scores from XGBoost models trained on 500 principal components derived from grey- and white-matter brain segmentations (single-model approach), highlighting that top components contribute most to prediction improvement (gain). **(a)** grey matter, **(b)** white matter, **(c)** combined grey and white matter

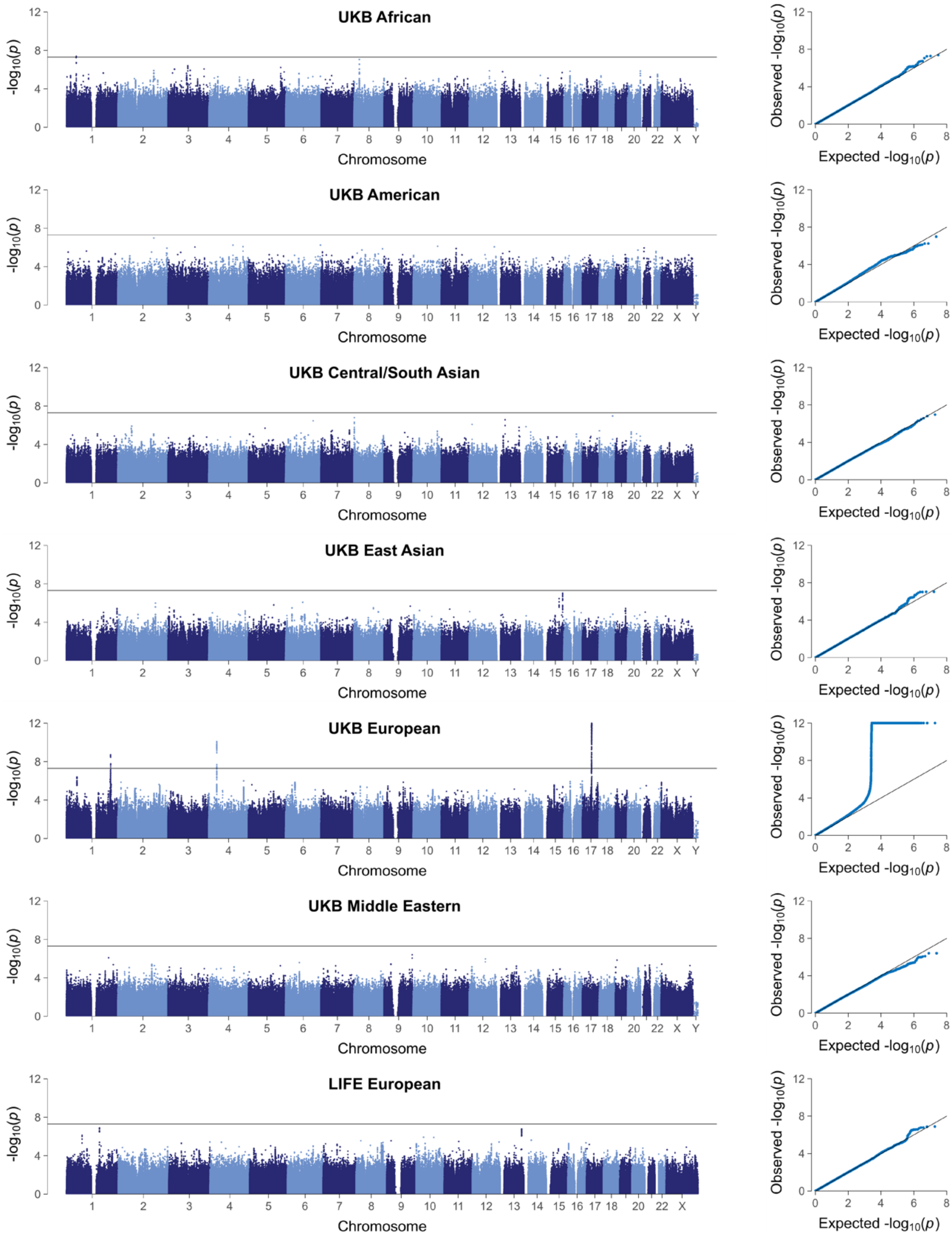

Fig. A17 Manhattan plots (left) and quantile-quantile plots (right) showing the results of the genome-wide association analyses for grey matter brain age gap in the seven replication samples: UKB African ancestry ($n = 337$), UKB Admixed American ancestry ($n = 94$); UKB Central/South Asian ancestry ($n = 638$), UKB East Asian ancestry ($n = 291$), UKB European ancestry ($n = 20{,}423$), UKB Middle Eastern ancestry ($n = 98$), and LIFE-Adult European ancestry ($n = 1{,}833$). Manhattan plots show the p-values (-$\log_{10}$ scale) of the tested genetic variations on the y-axis and base-pair positions along the chromosomes on the x-axis. The solid horizontal line indicates the threshold of genome-wide significance ($p = 5\text{E-}8$). Pseudoautosomal variations have been added to chromosome 'X'. Quantile-quantile plots show the observed p-values from the association analysis vs. the expected p-values under the null hypothesis of no effect (-$\log_{10}$ scale).

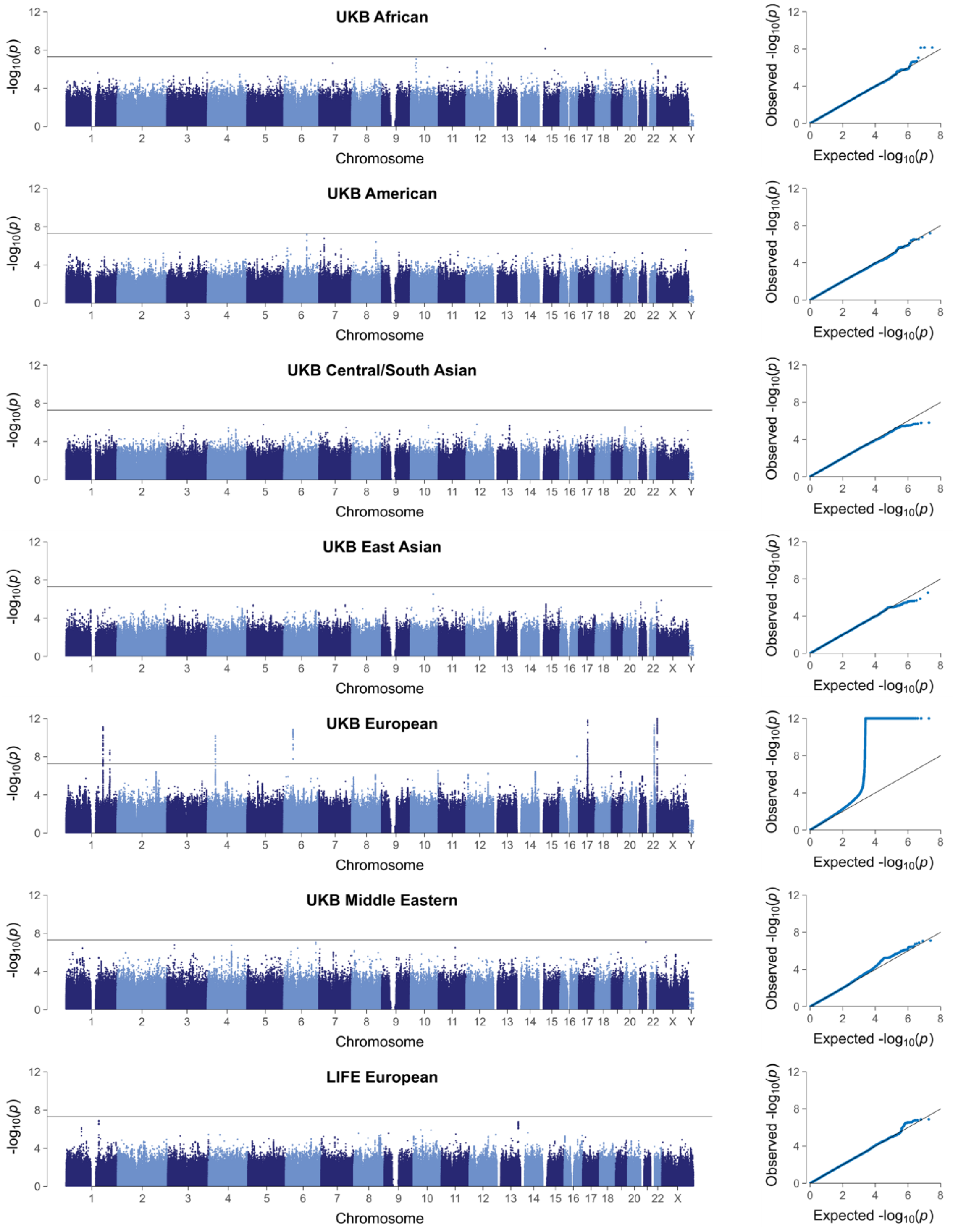

Fig. A18 Manhattan plots (left) and quantile-quantile plots (right) showing the results of the genome-wide association analyses for white matter brain age gap in the seven replication samples: UKB African ancestry ($n = 337$), UKB Admixed American ancestry ($n = 94$); UKB Central/South Asian ancestry ($n = 638$), UKB East Asian ancestry ($n = 291$), UKB European ancestry ($n = 20{,}423$), UKB Middle Eastern ancestry ($n = 98$), and LIFE-Adult European ancestry ($n = 1{,}833$). Manhattan plots show the p-values (-$\log_{10}$ scale) of the tested genetic variations on the y-axis and base-pair positions along the chromosomes on the x-axis. The solid horizontal line indicates the threshold of genome-wide significance ($p = $ 5E-8). Pseudoautosomal variations have been added to chromosome 'X'. Quantile-quantile plots show the observed p-values from the association analysis vs. the expected p-values under the null hypothesis of no effect (-$\log_{10}$ scale).

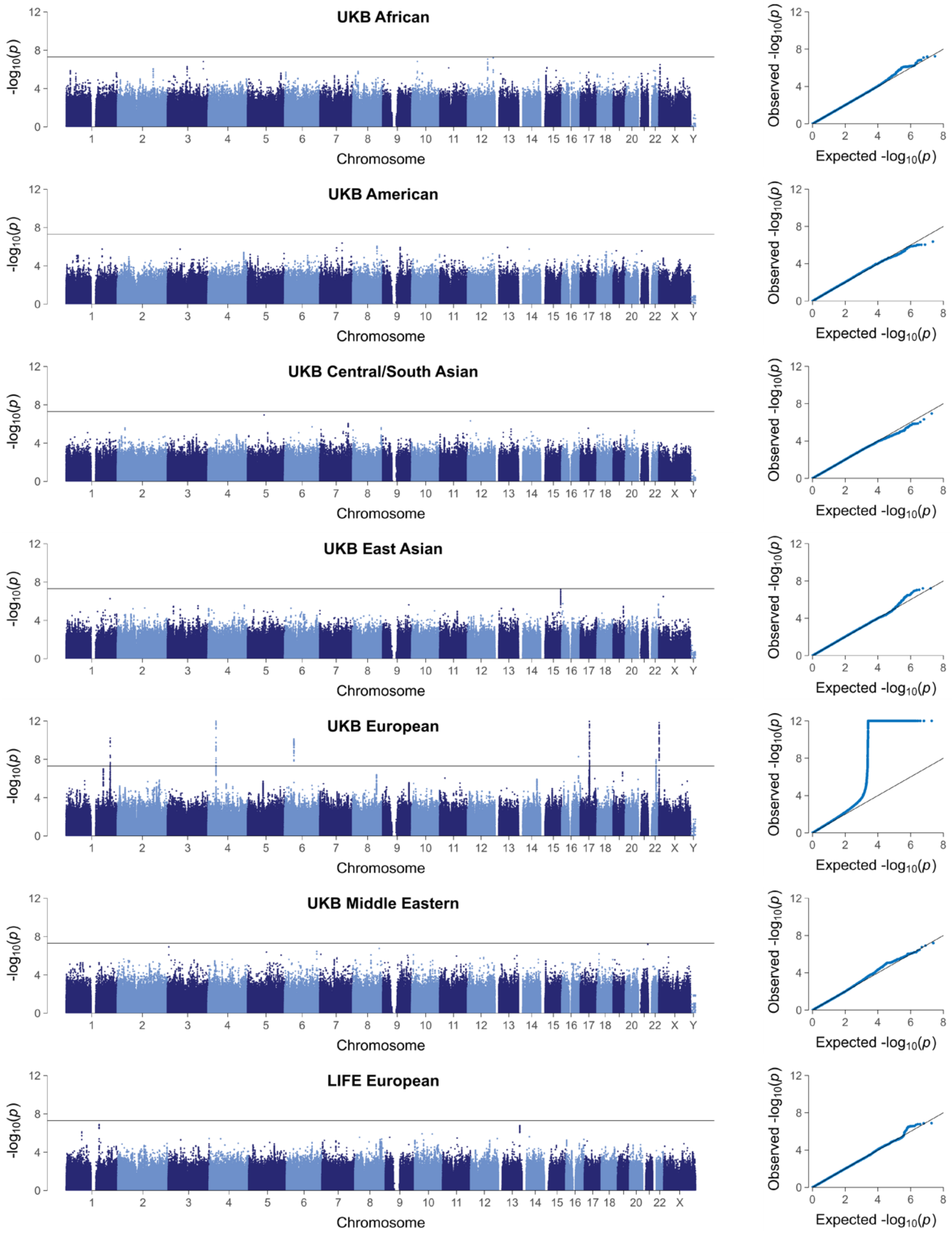

Fig. A19 Manhattan plots (left) and quantile-quantile plots (right) showing the results of the genome-wide association analyses for combined grey and white matter brain age gap in the seven replication samples: UKB African ancestry (n = 337), UKB Admixed American ancestry (n = 94); UKB Central/South Asian ancestry (n = 638), UKB East Asian ancestry (n = 291), UKB European ancestry (n = 20,423), UKB Middle Eastern ancestry (n = 98), and LIFE-Adult European ancestry (n = 1,833). Manhattan plots show the p-values ($-\log_{10}$ scale) of the tested genetic variations on the y-axis and base-pair positions along the chromosomes on the x-axis. The solid horizontal line indicates the threshold of genome-wide significance (p = 5E-8). Pseudoautosomal variations have been added to chromosome 'X'. Quantile-quantile plots show the observed p-values from the association analysis vs. the expected p-values under the null hypothesis of no effect ($-\log_{10}$ scale).

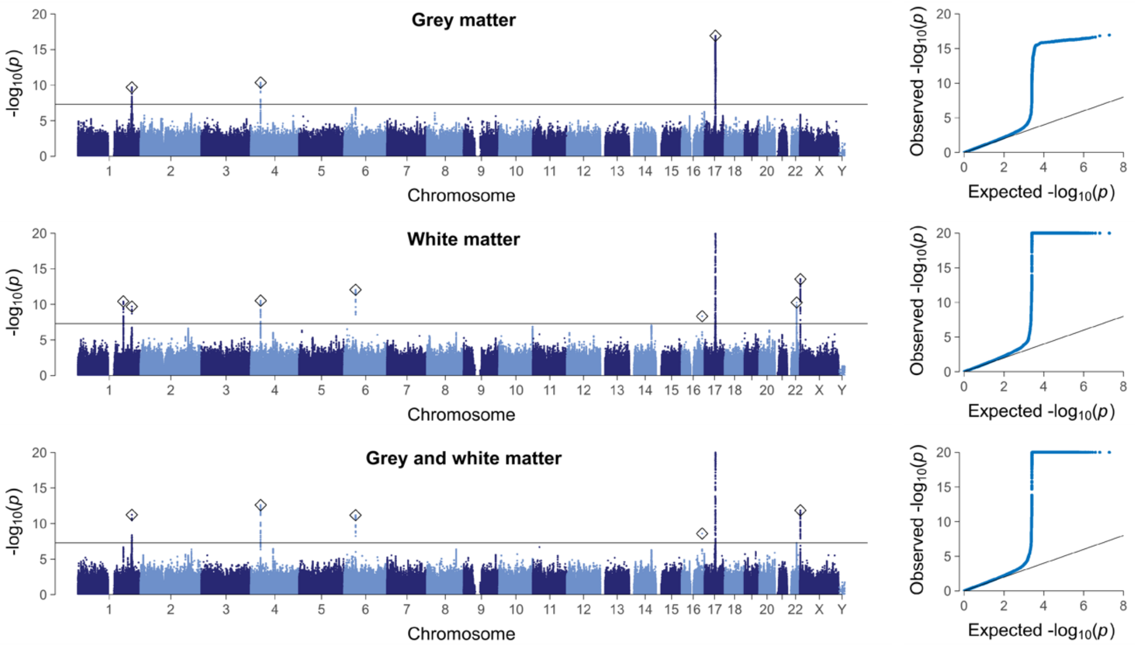

Fig. A20 Manhattan plots (left) and quantile-quantile plots (right) showing the results of the European ancestry replication genome-wide association meta-analyses (GWAMAs) for the three brain age gap traits. European ancestry replication GWAMAs combine results from two samples (total n = 22,256): the UKB European ancestry replication sample (n = 20,423), and LIFE-Adult (n = 1,833). Manhattan plots show the p-values (-$\log_{10}$ scale) of the tested genetic variations on the y-axis and base-pair positions along the chromosomes on the x-axis. The solid horizontal line indicates the threshold of genome-wide significance (p = 5E-8). Pseudoautosomal variations have been added to chromosome 'X'. Quantile-quantile plots show the observed p-values from the association analysis vs. the expected p-values under the null hypothesis of no effect (-$\log_{10}$ scale). For illustrative reasons, the y-axis has been truncated at p = 1.0e-20.

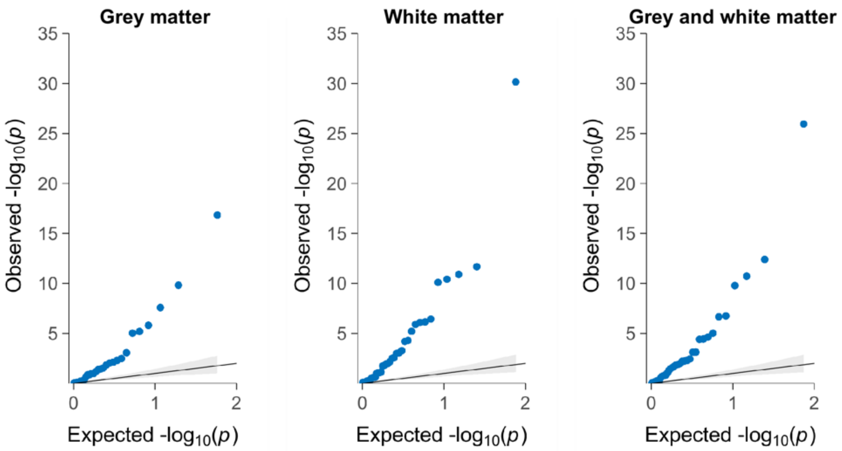

Fig. A21 Quantile-quantile plots showing the European ancestry replication results (up to n = 22,256) for independent variants with discovery p < 1.0e-06. Quantile-quantile plots show the observed p-values from the association analysis vs. the expected p-values under the null hypothesis of no effect (-$\log_{10}$ scale). Blue dots reflect the observed p-values sorted from largest to smallest and plotted against the expected p-values under the null hypothesis of no effect (-$\log_{10}$ scale). The solid diagonal line reflects the mean expected p-values. The lower and upper bound of the grey shaded area represent the 5th and 95th percentile of the expected p-values. The quantile-quantile plots show an excess of low p-values observed in replication analyses, suggesting stronger evidence than expected under the null hypothesis of no effect.

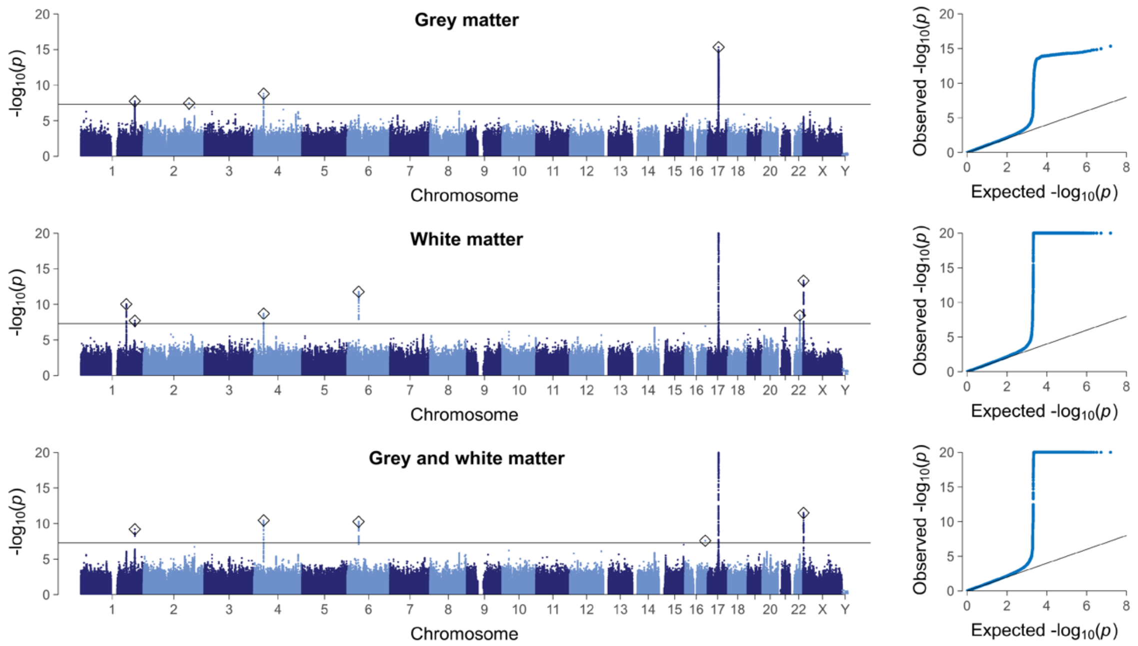

Fig. A22 Manhattan plots (left) and quantile-quantile plots (right) showing the results of the multi-ancestry replication meta-regression (MR-MEGA) for the three brain age gap traits. Multi-ancestry replication analyses combine results from up to seven samples (total $n = 23{,}714$): UKB African ancestry ($n = 337$), UKB Admixed American ancestry ($n = 94$); UKB Central/South Asian ancestry ($n = 638$), UKB East Asian ancestry ($n = 291$), UKB European ancestry ($n = 20{,}423$), UKB Middle Eastern ancestry ($n = 98$), and LIFE-Adult (European ancestry; $n = 1{,}833$). Manhattan plots show the p-values (-$\log_{10}$ scale) of the tested genetic variations on the y-axis and base-pair positions along the chromosomes on the x-axis. The solid horizontal line indicates the threshold of genome-wide significance (p = 5E-8). Pseudoautosomal variations have been added to chromosome 'X'. Quantile-quantile plots show the observed p-values from the association analysis vs. the expected p-values under the null hypothesis of no effect (-$\log_{10}$ scale).

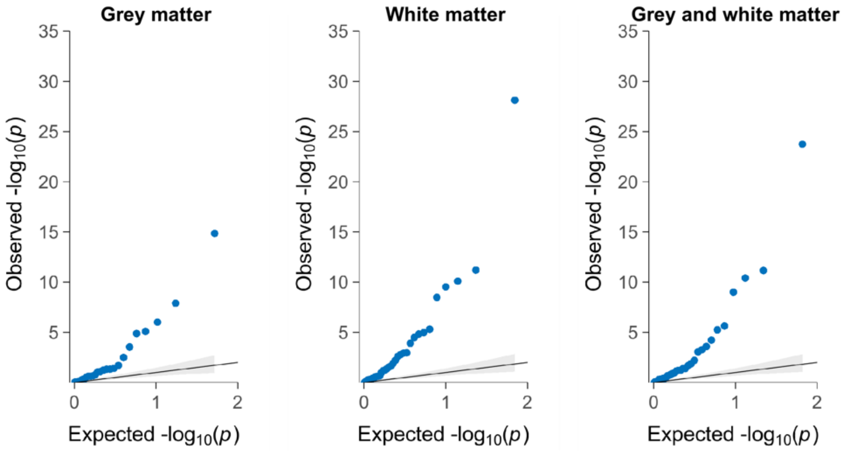

Fig. A23 Quantile-quantile plots showing the multi-ancestry replication results (up to $n = 23{,}714$) for independent variants with discovery $p <$ 1.0e-06. Quantile-quantile plots show the observed p-values from the association analysis vs. the expected p-values under the null hypothesis of no effect (-$\log_{10}$ scale). Blue dots reflect the observed p-values sorted from largest to smallest and plotted against the expected p-values under the null hypothesis of no effect (-$\log_{10}$ scale). The solid diagonal line reflects the mean expected p-values. The lower and upper bound of the grey shaded area represent the 5th and 95th percentile of the expected p-values. The quantile-quantile plots show an excess of low p-values observed in replication analyses, suggesting stronger evidence than expected under the null hypothesis of no effect.

GWAS across discovery and replication: Identification of 59 associated loci

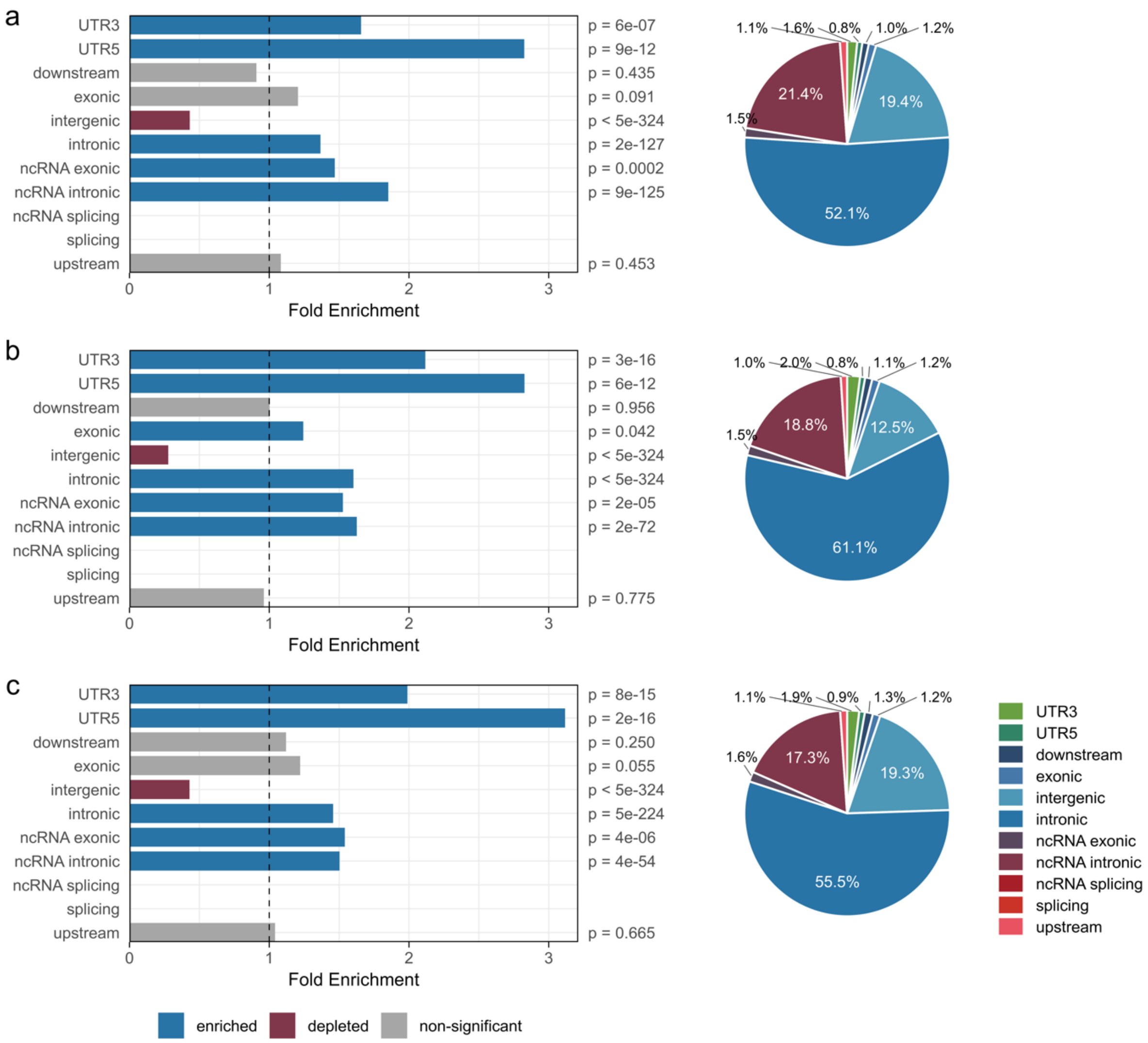

Fig. A24 Results of the ANNOVAR enrichment test of functional consequences of discovered variants in $n = 54{,}890$ European ancestry individuals. All ‘candidate-SNPs’ (see methods for definition) identified by FUMA in genome-wide significant loci have been considered for the ANNOVAR enrichment test. Bar diagrams show the Fold Enrichment (*FE*), i.e., the ratio of the observed proportion vs. the expected proportion of candidate-SNPs annotated with the respective functional consequence. *FE* values higher than 1 indicate an enrichment, whereas *FE* values lower than 1 indicate a depletion of the respective functional consequence. Pie charts show the distribution of variations annotated with the respective functional consequences. **(a)** grey matter brain age gap (7,230 candidate-SNPs), **(b)** white matter brain age gap (7,384 candidate-SNPs), and **(c)** combined grey and white matter brain age gap (8,244 candidate-SNPs)

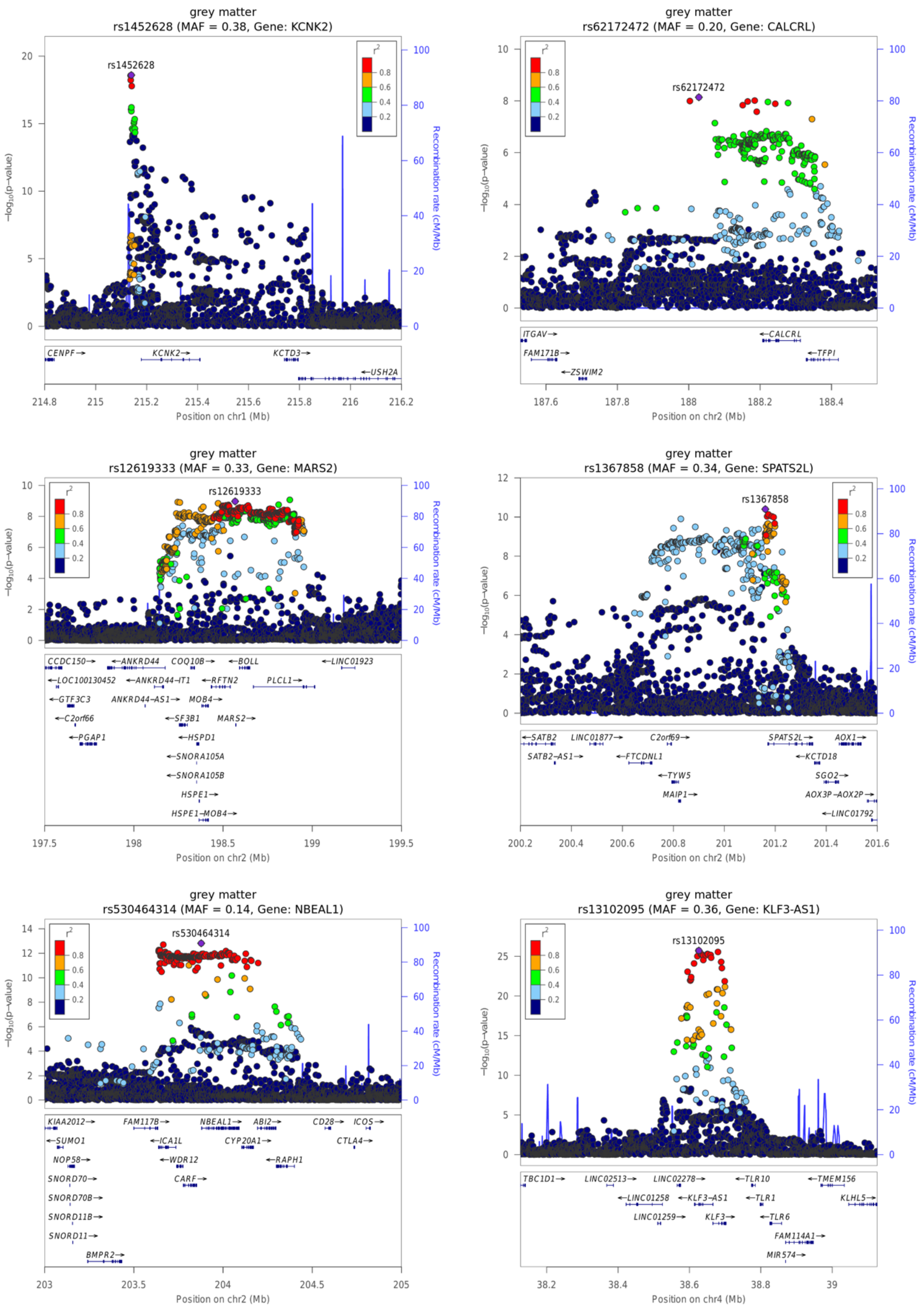

Fig. A25 Regional association plots for index variations 1-6 from the genome-wide association meta-analysis of grey matter brain age gap in up to $n = 54{,}890$ European ancestry individuals. Regional association plots were created using Locuszoom Standalone (v1.4). SNP positions (dbSNP build 151) and refFlat gene locations (2020-08-17) are based on human genome build hg19 and were accessed via UCSC Genome Browser. Recombination rates were derived from HapMap phase II build GRCh37 (2011-01-19). MAF: Minor allele frequency, r^2: linkage disequilibrium between index variation and other variation in locus.

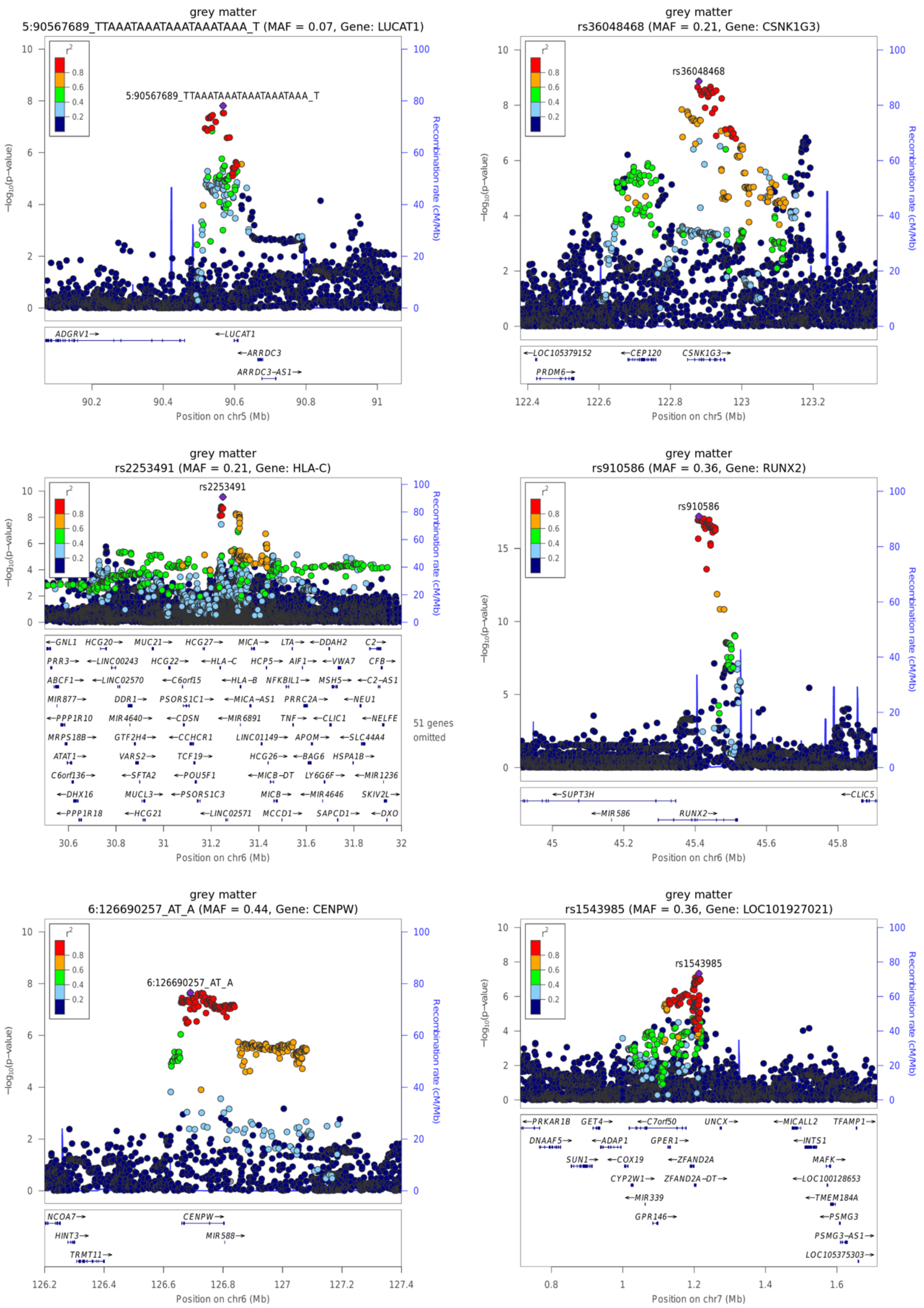

Fig. A26 Regional association plots for index variations 7-12 from the genome-wide association meta-analysis of grey matter brain age gap in up to $n = 54{,}890$ European ancestry individuals. Regional association plots were created using Locuszoom Standalone (v1.4). SNP positions (dbSNP build 151) and refFlat gene locations (2020-08-17) are based on human genome build hg19 and were accessed via UCSC Genome Browser. Recombination rates were derived from HapMap phase II build GRCh37 (2011-01-19). MAF: Minor allele frequency, r^2: linkage disequilibrium between index variation and other variation in locus

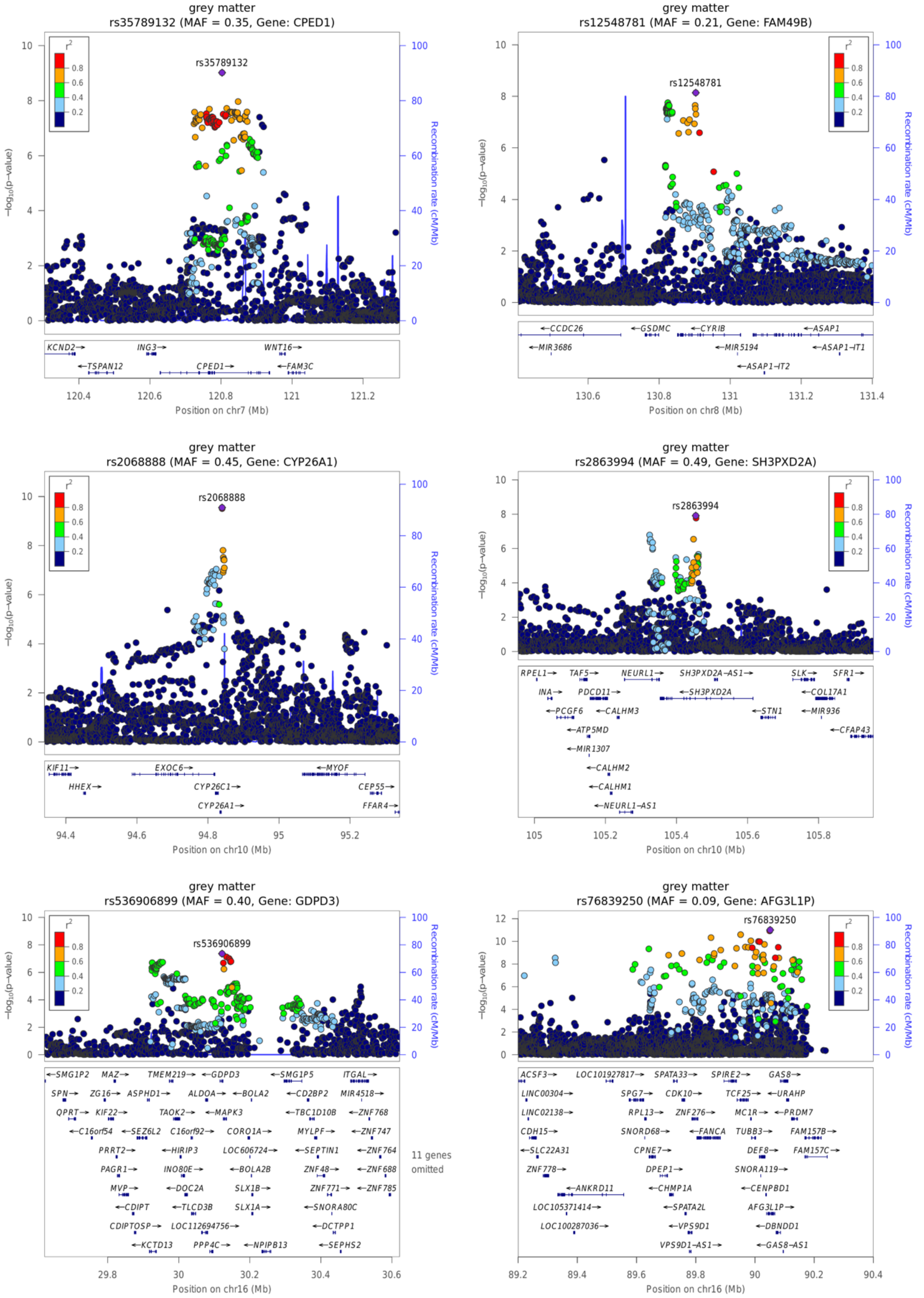

Fig. A27 Regional association plots for index variations 13-18 from the genome-wide association meta-analysis of grey matter brain age gap in up to $n = 54{,}890$ European ancestry individuals. Regional association plots were created using Locuszoom Standalone (v1.4). SNP positions (dbSNP build 151) and refFlat gene locations (2020-08-17) are based on human genome build hg19 and were accessed via UCSC Genome Browser. Recombination rates were derived from HapMap phase II build GRCh37 (2011-01-19). MAF: Minor allele frequency, r^2: linkage disequilibrium between index variation and other variation in locus

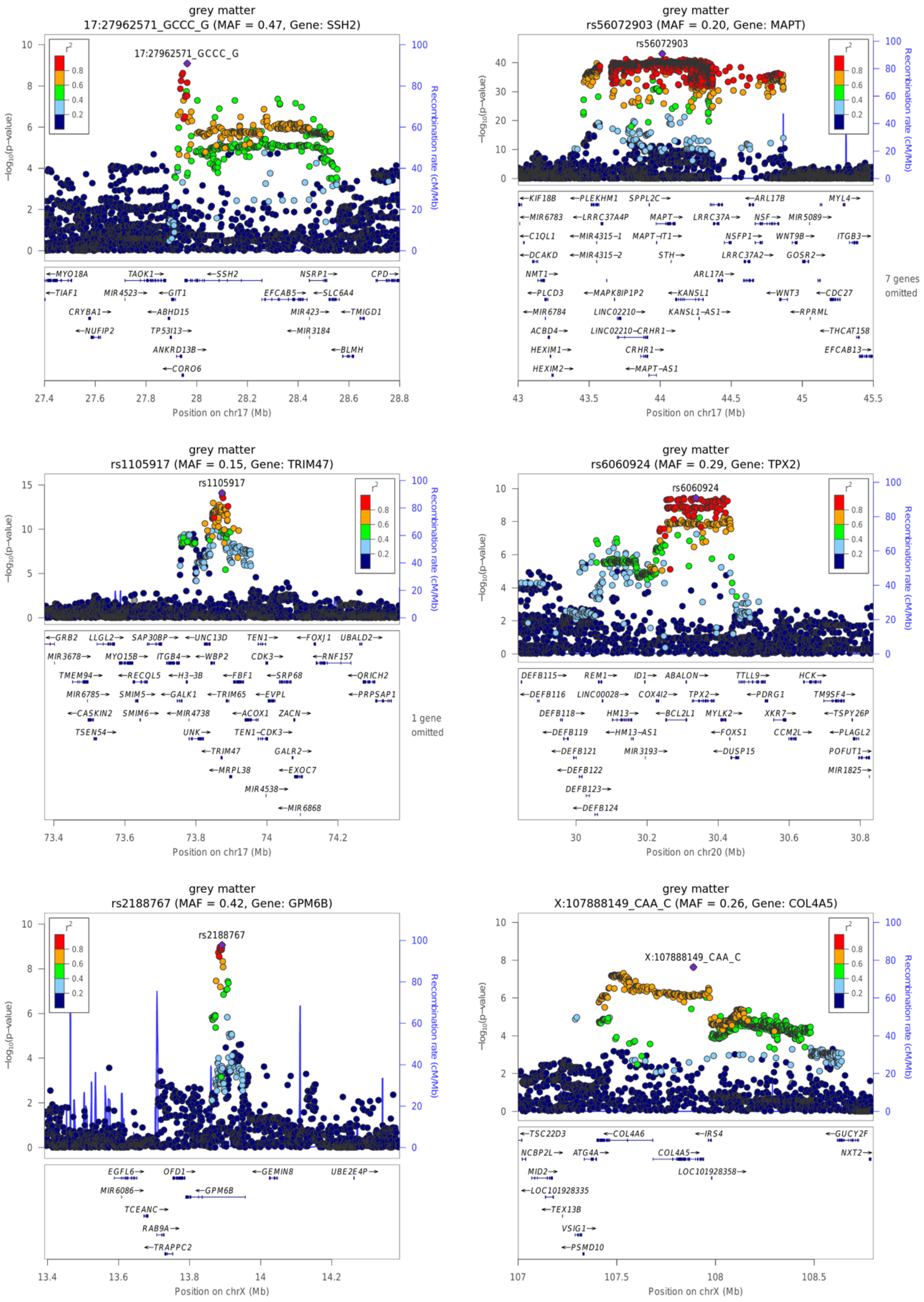

Fig. A28 Regional association plots for index variations 19-24 from the genome-wide association meta-analysis of grey matter brain age gap in up to $n = 54{,}890$ European ancestry individuals. Regional association plots were created using Locuszoom Standalone (v1.4). SNP positions (dbSNP build 151) and refFlat gene locations (2020-08-17) are based on human genome build hg19 and were accessed via UCSC Genome Browser. Recombination rates were derived from HapMap phase II build GRCh37 (2011-01-19). MAF: Minor allele frequency, r^2: linkage disequilibrium between index variation and other variation in locus

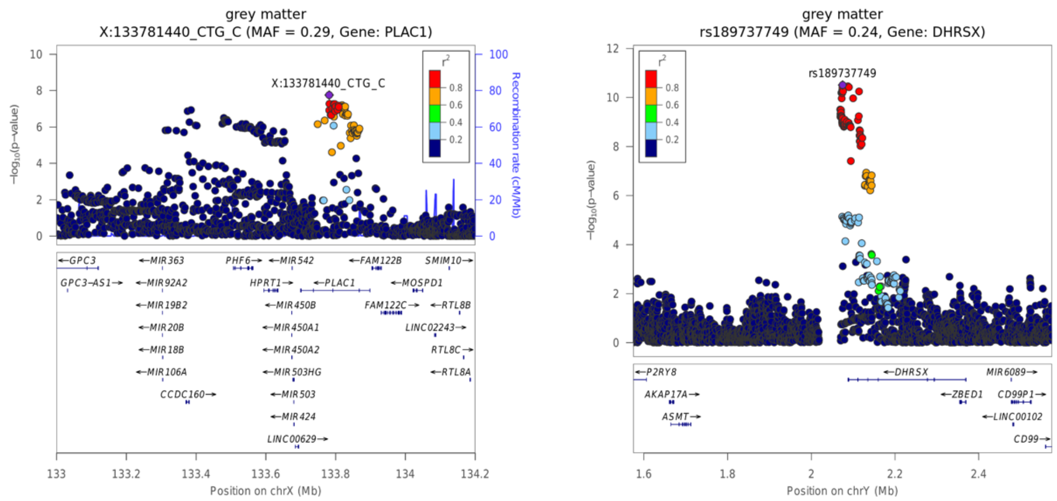

Fig. A29 Regional association plots for index variations 25-26 from the genome-wide association meta-analysis of grey matter brain age gap in up to $n = 54{,}890$ European ancestry individuals. Regional association plots were created using Locuszoom Standalone (v1.4). SNP positions (dbSNP build 151) and refFlat gene locations (2020-08-17) are based on human genome build hg19 and were accessed via UCSC Genome Browser. Recombination rates were derived from HapMap phase II build GRCh37 (2011-01-19). MAF: Minor allele frequency, r^2: linkage disequilibrium between index variation and other variation in locus

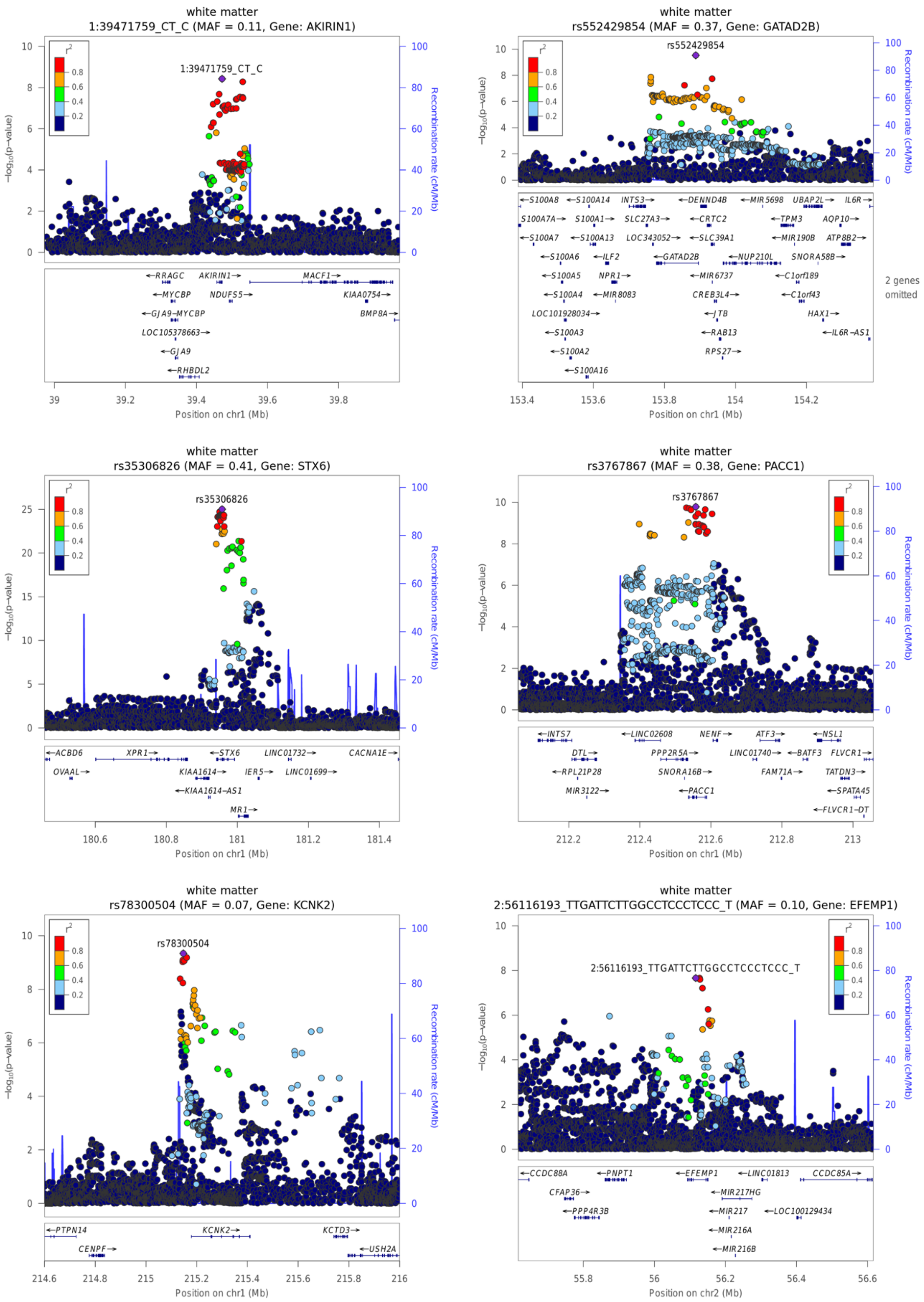

Fig. A30 Regional association plots for index variations 1-6 from the genome-wide association meta-analysis of white matter brain age gap in up to $n = 54{,}890$ European ancestry individuals. Regional association plots were created using Locuszoom Standalone (v1.4). SNP positions (dbSNP build 151) and refFlat gene locations (2020-08-17) are based on human genome build hg19 and were accessed via UCSC Genome Browser. Recombination rates were derived from HapMap phase II build GRCh37 (2011-01-19). MAF: Minor allele frequency, r^2: linkage disequilibrium between index variation and other variation in locus

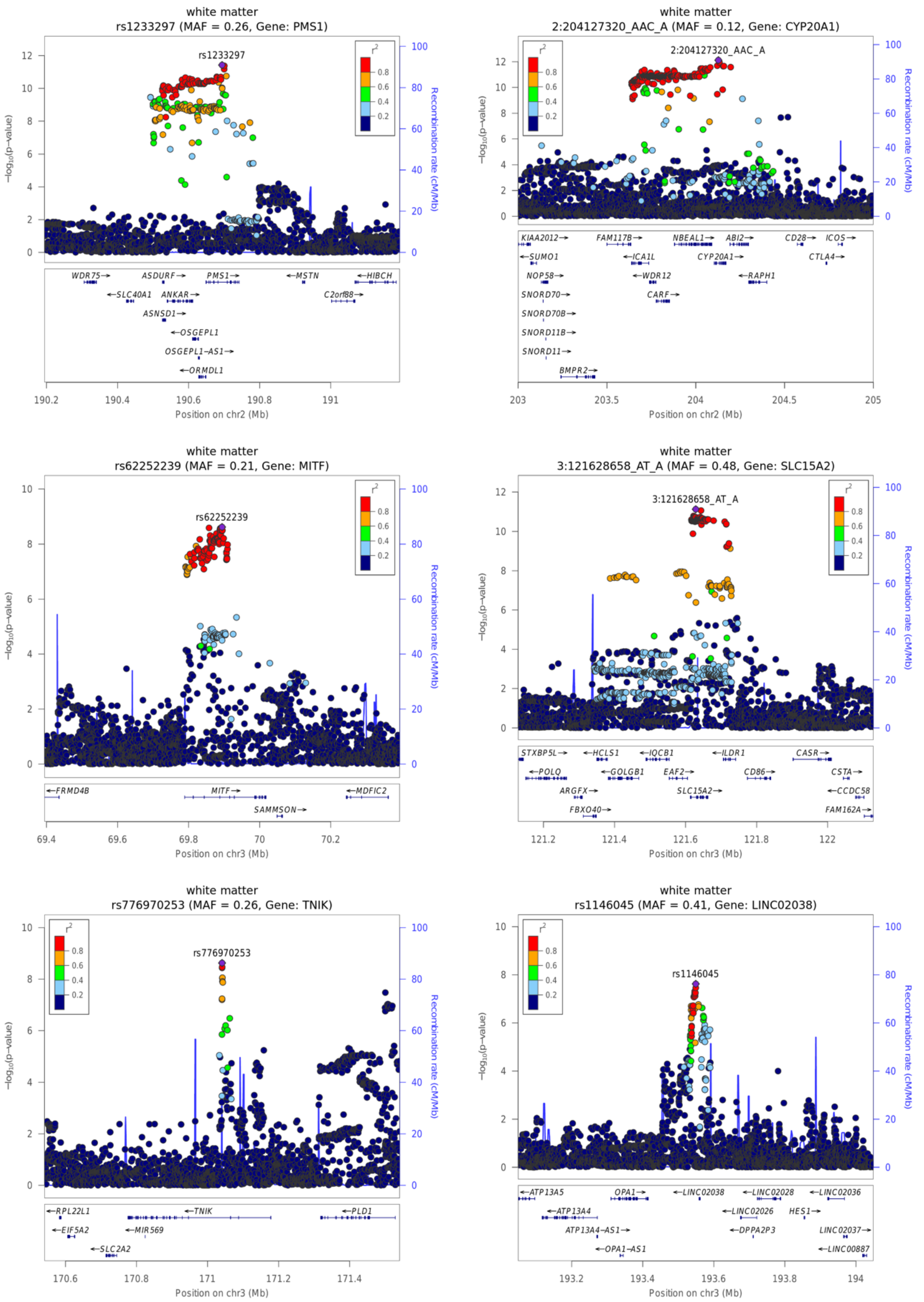

Fig. A31 Regional association plots for index variations 7-12 from the genome-wide association meta-analysis of white matter brain age gap in up to $n = 54{,}890$ European ancestry individuals. Regional association plots were created using Locuszoom Standalone (v1.4). SNP positions (dbSNP build 151) and refFlat gene locations (2020-08-17) are based on human genome build hg19 and were accessed via UCSC Genome Browser. Recombination rates were derived from HapMap phase II build GRCh37 (2011-01-19). MAF: Minor allele frequency, r^2: linkage disequilibrium between index variation and other variation in locus

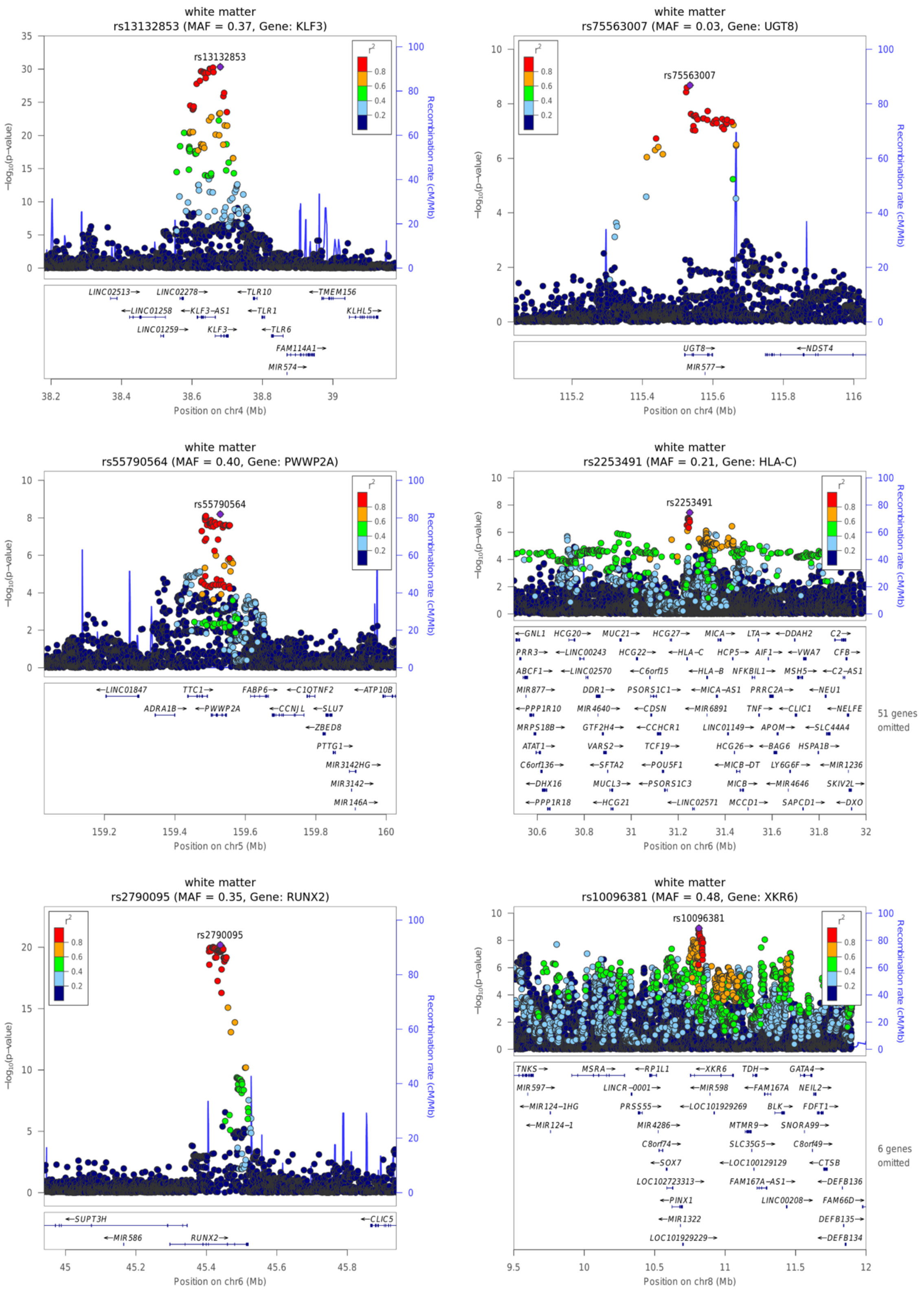

Fig. A32 Regional association plots for index variations 13-18 from the genome-wide association meta-analysis of white matter brain age gap in up to $n = 54{,}890$ European ancestry individuals. Regional association plots were created using Locuszoom Standalone (v1.4). SNP positions (dbSNP build 151) and refFlat gene locations (2020-08-17) are based on human genome build hg19 and were accessed via UCSC Genome Browser. Recombination rates were derived from HapMap phase II build GRCh37 (2011-01-19). MAF: Minor allele frequency, r^2: linkage disequilibrium between index variation and other variation in locus

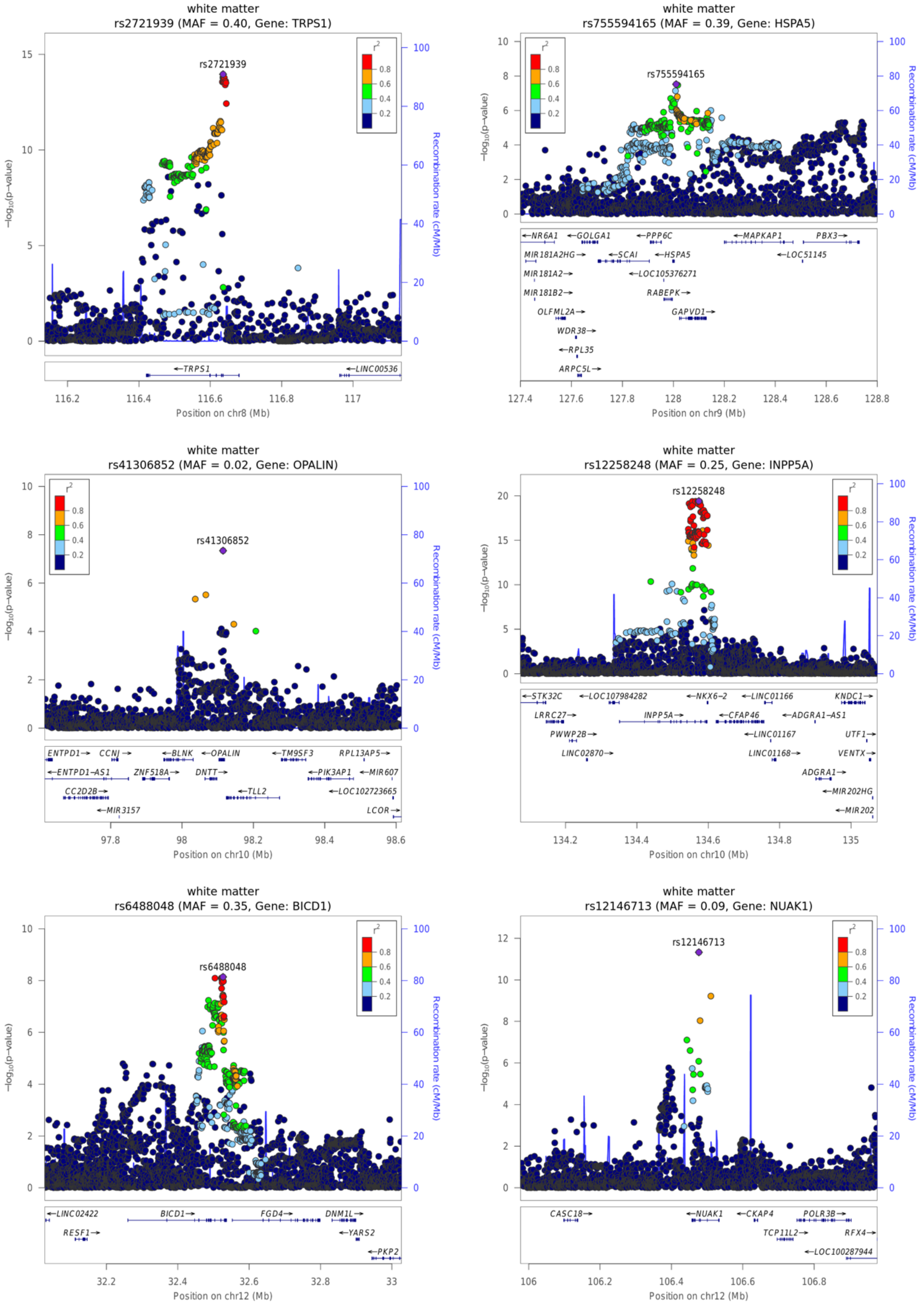

Fig. A33 Regional association plots for index variations 19-24 from the genome-wide association meta-analysis of white matter brain age gap in up to $n = 54{,}890$ European ancestry individuals. Regional association plots were created using Locuszoom Standalone (v1.4). SNP positions (dbSNP build 151) and refFlat gene locations (2020-08-17) are based on human genome build hg19 and were accessed via UCSC Genome Browser. Recombination rates were derived from HapMap phase II build GRCh37 (2011-01-19). MAF: Minor allele frequency, r^2: linkage disequilibrium between index variation and other variation in locus

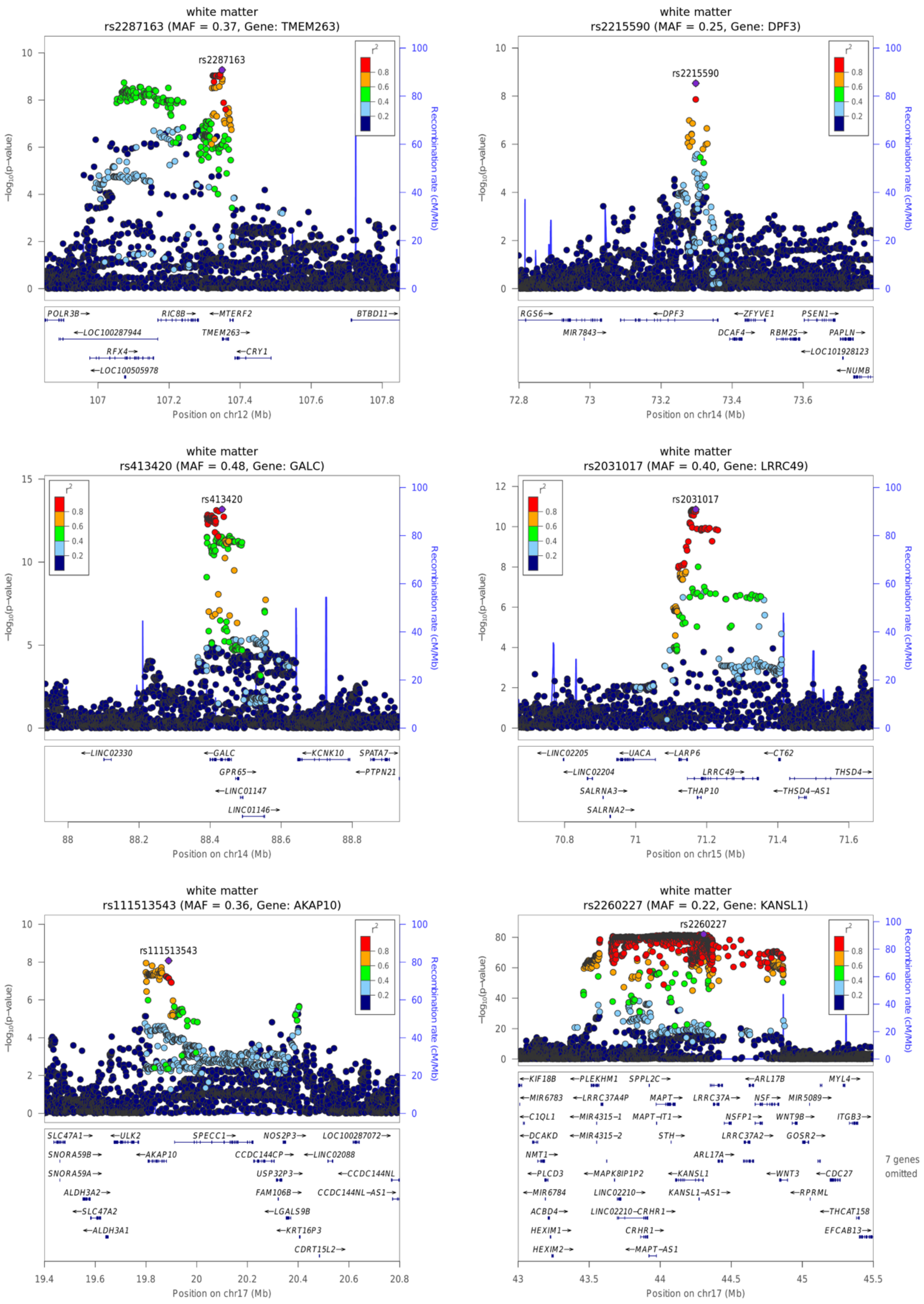

Fig. A34 Regional association plots for index variations 25-30 from the genome-wide association meta-analysis of white matter brain age gap in up to $n = 54{,}890$ European ancestry individuals. Regional association plots were created using Locuszoom Standalone (v1.4). SNP positions (dbSNP build 151) and refFlat gene locations (2020-08-17) are based on human genome build hg19 and were accessed via UCSC Genome Browser. Recombination rates were derived from HapMap phase II build GRCh37 (2011-01-19). MAF: Minor allele frequency, r^2: linkage disequilibrium between index variation and other variation in locus

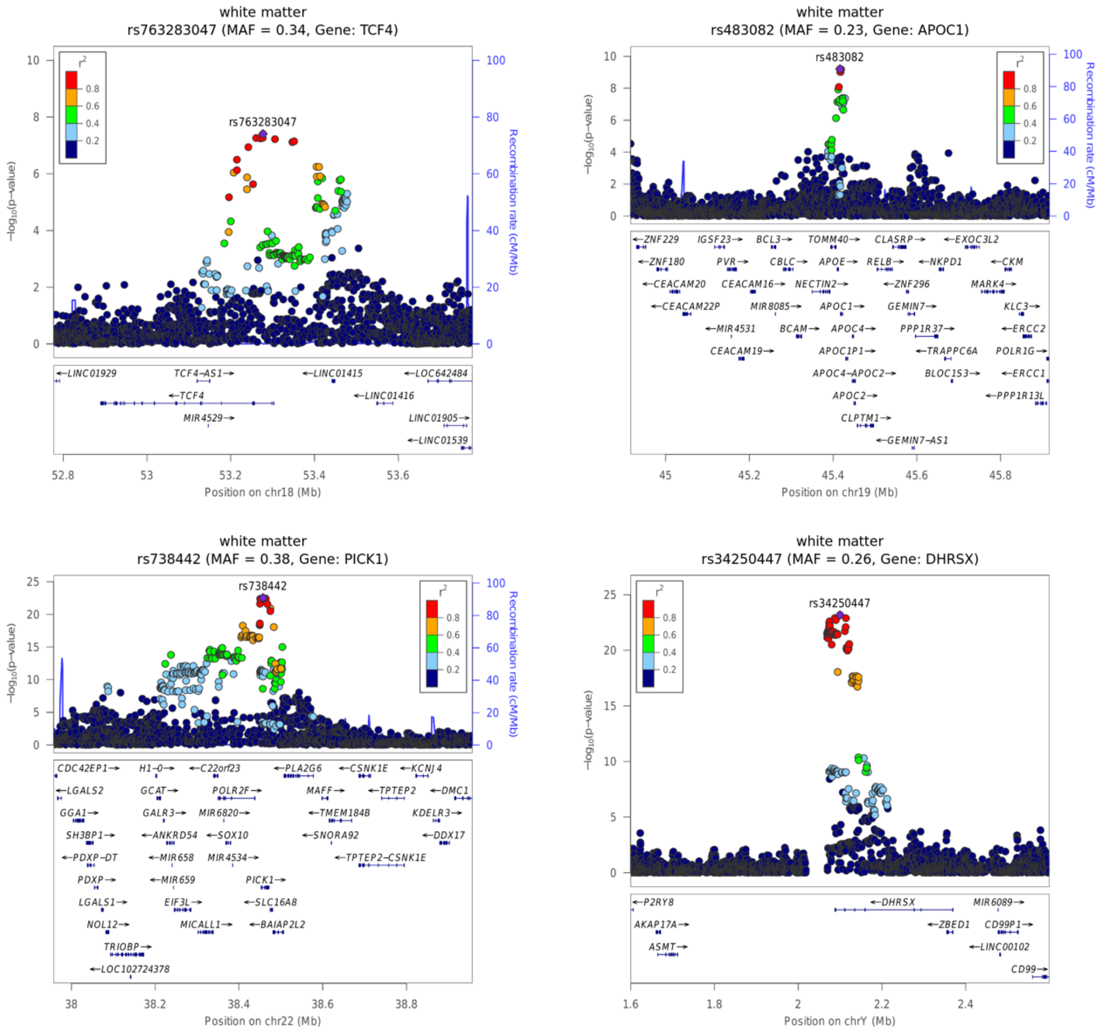

Fig. A35 Regional association plots for index variations 31-34 from the genome-wide association meta-analysis of white matter brain age gap in up to $n = 54{,}890$ European ancestry individuals. Regional association plots were created using Locuszoom Standalone (v1.4). SNP positions (dbSNP build 151) and refFlat gene locations (2020-08-17) are based on human genome build hg19 and were accessed via UCSC Genome Browser. Recombination rates were derived from HapMap phase II build GRCh37 (2011-01-19). MAF: Minor allele frequency, r^2: linkage disequilibrium between index variation and other variation in locus

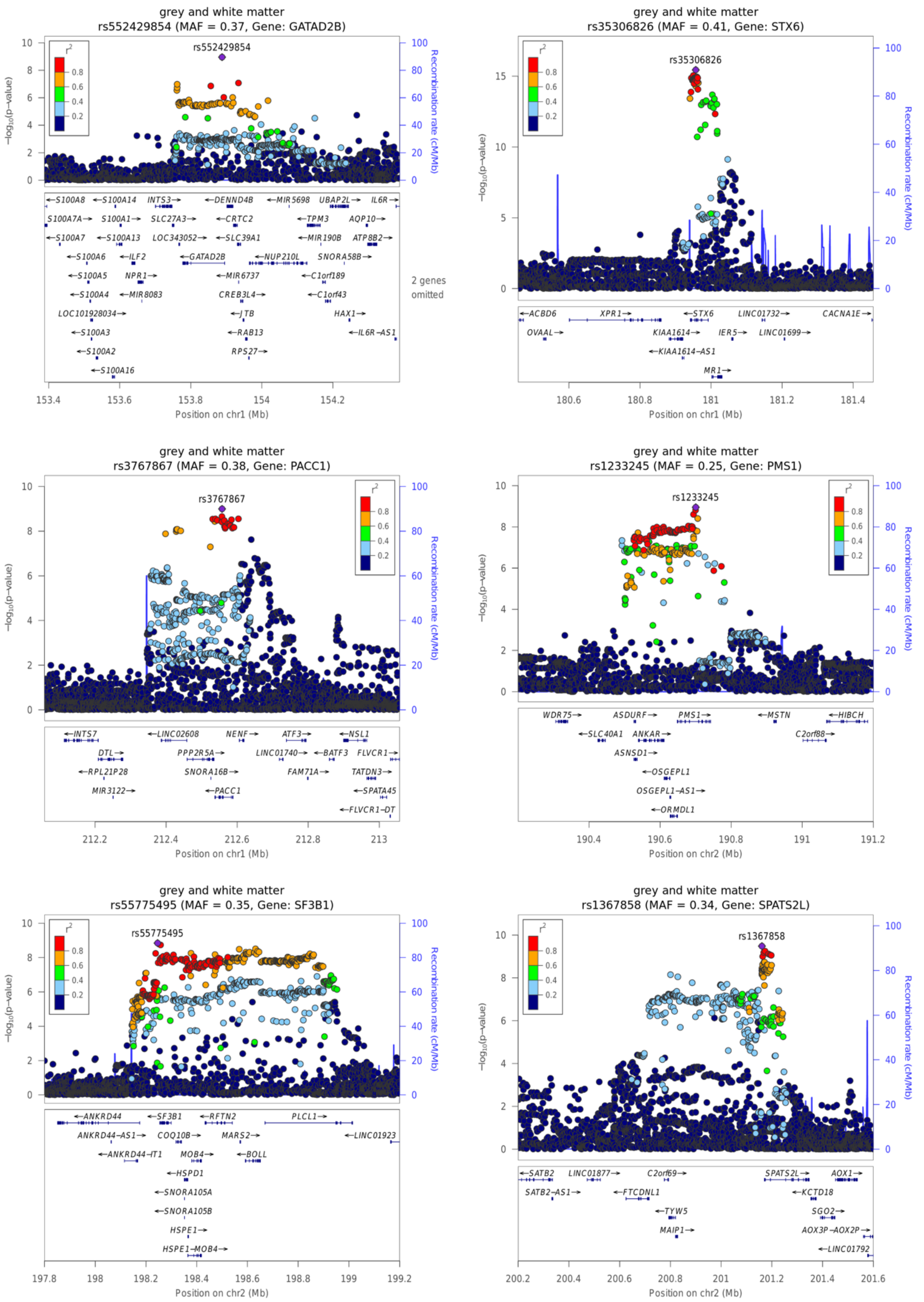

Fig. A36 Regional association plots for index variations 1-6 from the genome-wide association meta-analysis of combined grey and white matter brain age gap in up to $n = 54{,}890$ European ancestry individuals. Regional association plots were created using Locuszoom Standalone (v1.4). SNP positions (dbSNP build 151) and refFlat gene locations (2020-08-17) are based on human genome build hg19 and were accessed via UCSC Genome Browser. Recombination rates were derived from HapMap phase II build GRCh37 (2011-01-19). MAF: Minor allele frequency, r^2: linkage disequilibrium between index variation and other variation in locus

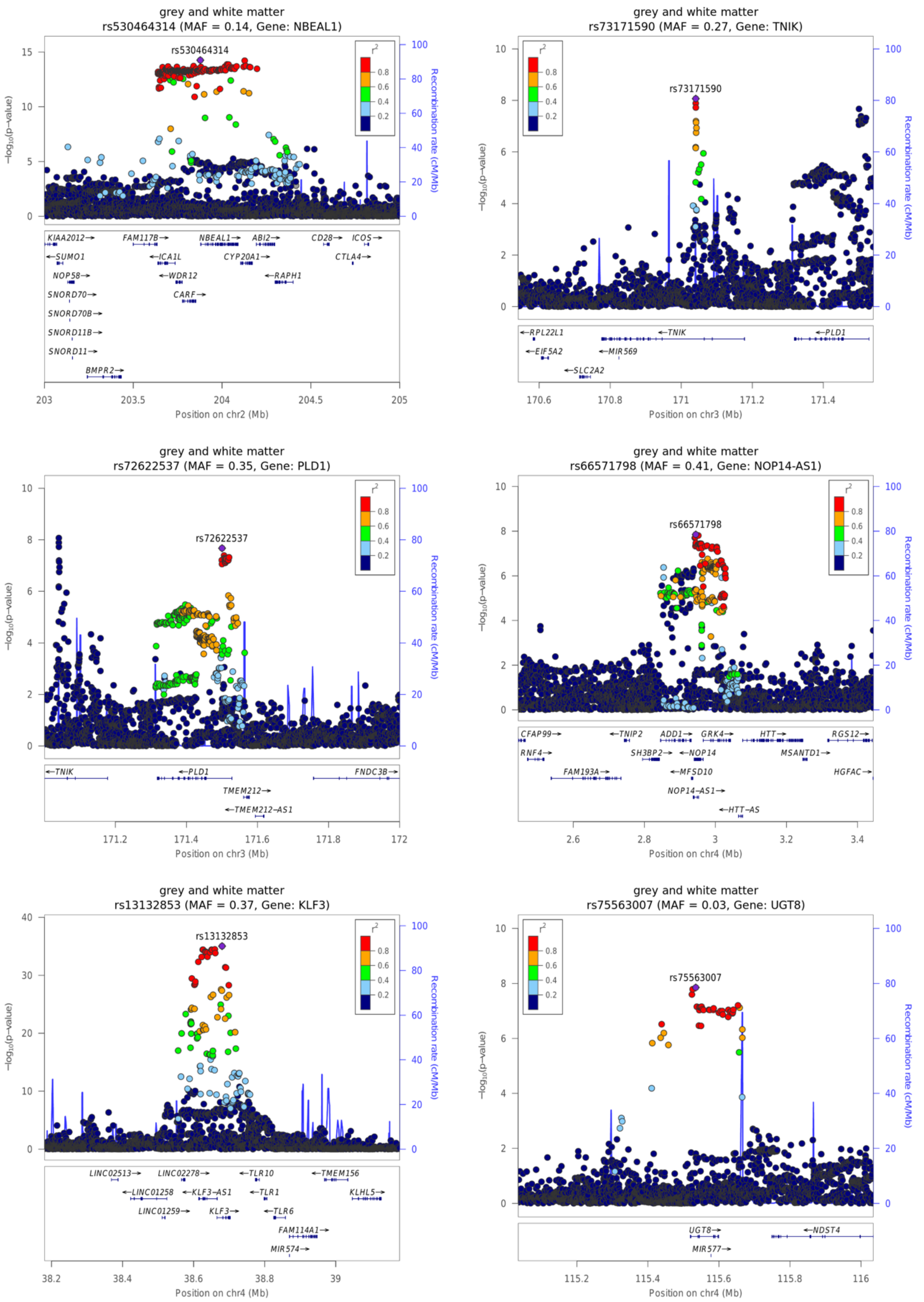

Fig. A37 Regional association plots for index variations 7-12 from the genome-wide association meta-analysis of combined grey and white matter brain age gap in up to $n = 54{,}890$ European ancestry individuals. Regional association plots were created using Locuszoom Standalone (v1.4). SNP positions (dbSNP build 151) and refFlat gene locations (2020-08-17) are based on human genome build hg19 and were accessed via UCSC Genome Browser. Recombination rates were derived from HapMap phase II build GRCh37 (2011-01-19). MAF: Minor allele frequency, r^2: linkage disequilibrium between index variation and other variation in locus

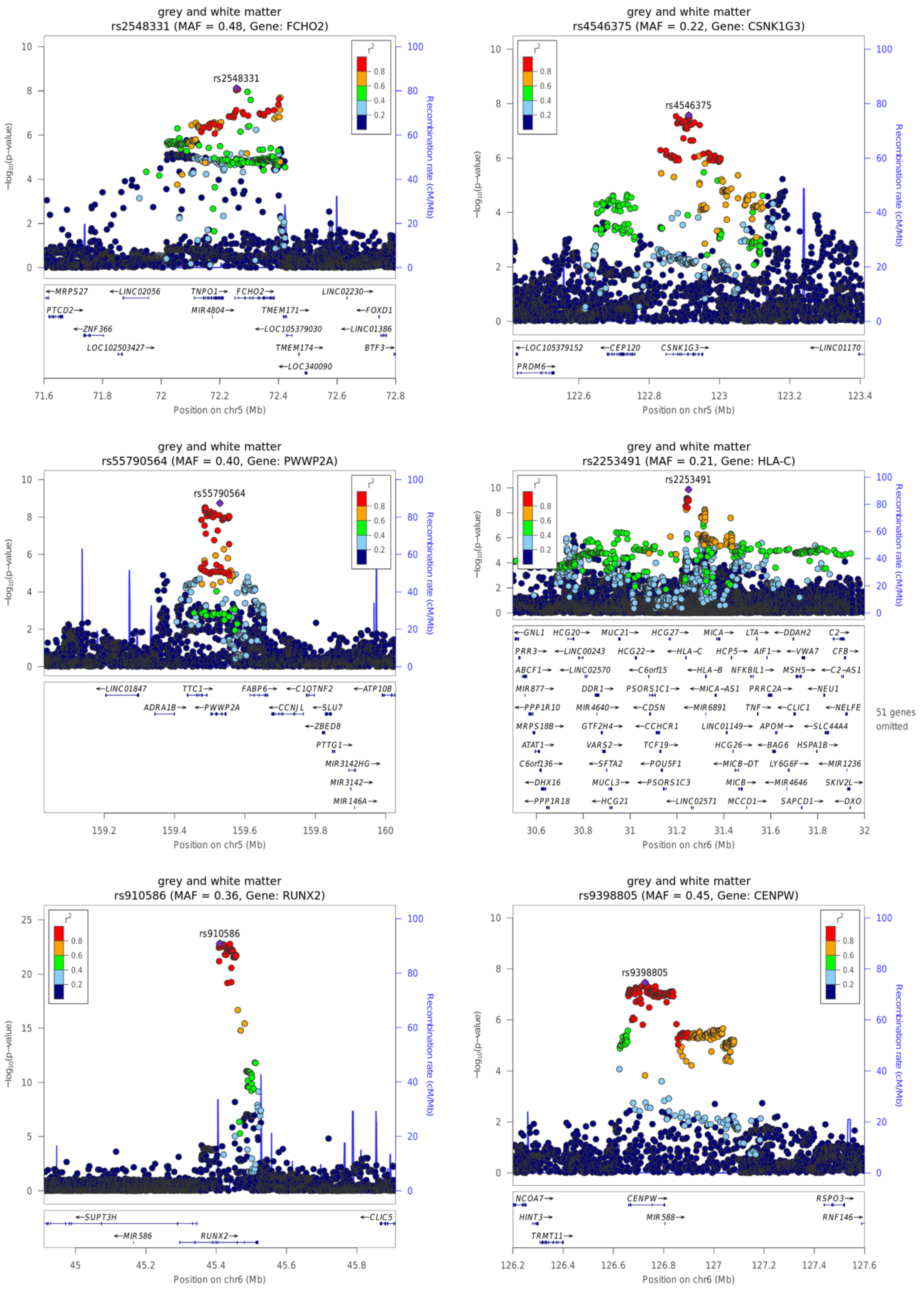

Fig. A38 Regional association plots for index variations 13-18 from the genome-wide association meta-analysis of combined grey and white matter brain age gap in up to $n = 54{,}890$ European ancestry individuals. Regional association plots were created using Locuszoom Standalone (v1.4). SNP positions (dbSNP build 151) and refFlat gene locations (2020-08-17) are based on human genome build hg19 and were accessed via UCSC Genome Browser. Recombination rates were derived from HapMap phase II build GRCh37 (2011-01-19). MAF: Minor allele frequency, r^2: linkage disequilibrium between index variation and other variation in locus

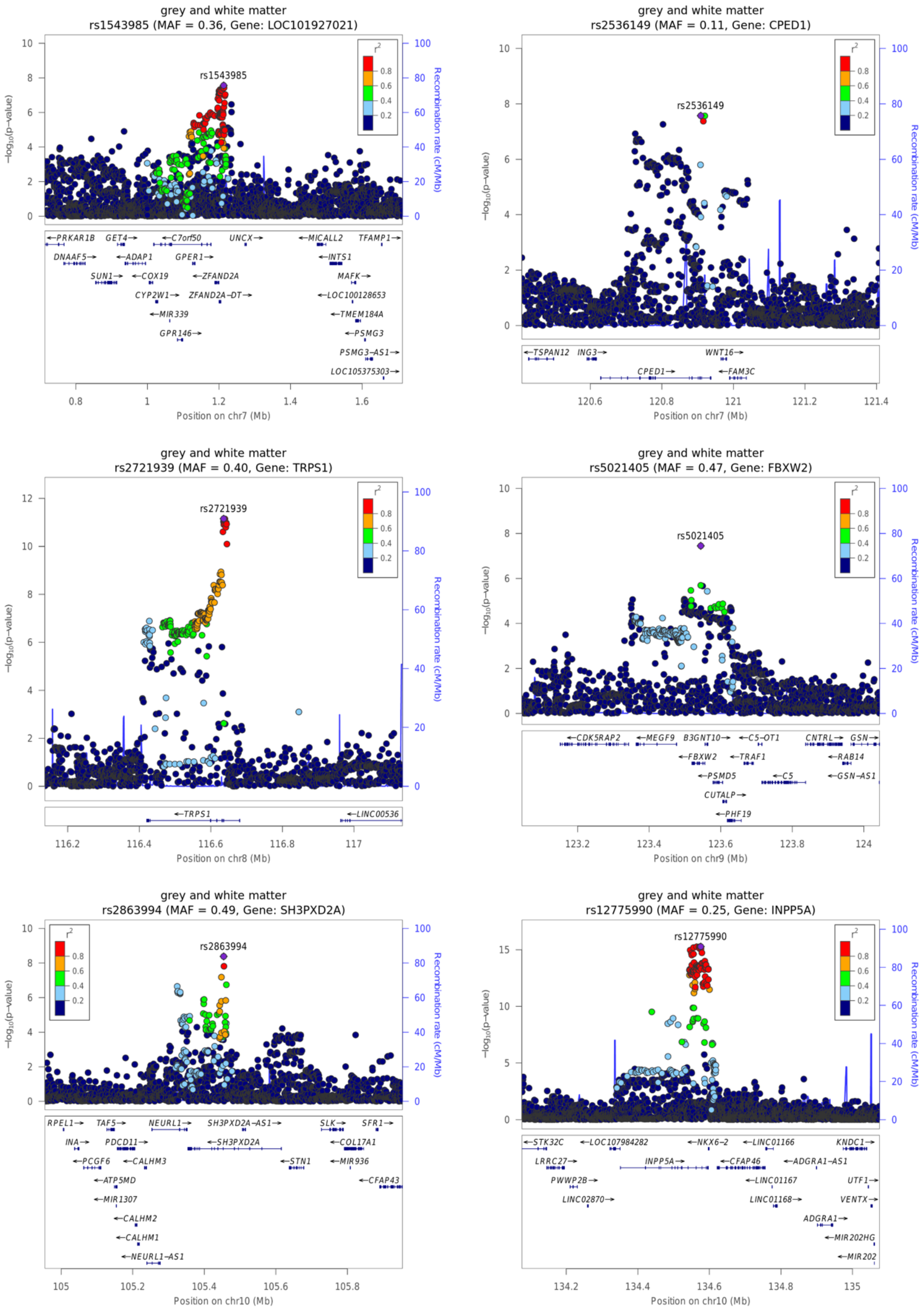

Fig. A39 Regional association plots for index variations 19-24 from the genome-wide association meta-analysis of combined grey and white matter brain age gap in up to n= 54,890 European ancestry individuals. Regional association plots were created using Locuszoom Standalone (v1.4). SNP positions (dbSNP build 151) and refFlat gene locations (2020-08-17) are based on human genome build hg19 and were accessed via UCSC Genome Browser. Recombination rates were derived from HapMap phase II build GRCh37 (2011-01-19). MAF: Minor allele frequency, r^2: linkage disequilibrium between index variation and other variation in locus.

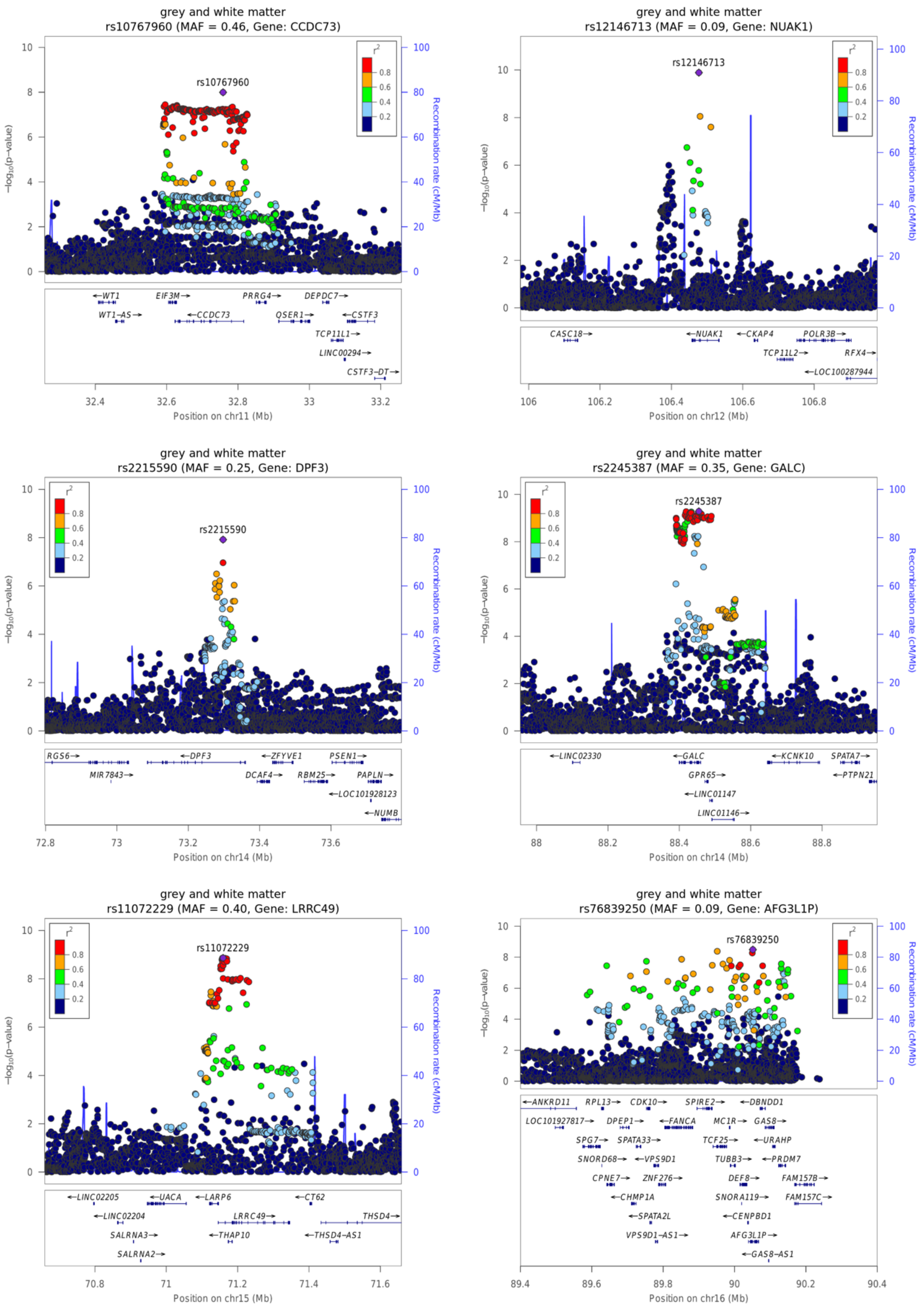

Fig. A40 Regional association plots for index variations 25-30 from the genome-wide association meta-analysis of combined grey and white matter brain age gap in up to $n = 54{,}890$ European ancestry individuals. Regional association plots were created using Locuszoom Standalone (v1.4). SNP positions (dbSNP build 151) and refFlat gene locations (2020-08-17) are based on human genome build hg19 and were accessed via UCSC Genome Browser. Recombination rates were derived from HapMap phase II build GRCh37 (2011-01-19). MAF: Minor allele frequency, r^2: linkage disequilibrium between index variation and other variation in locus

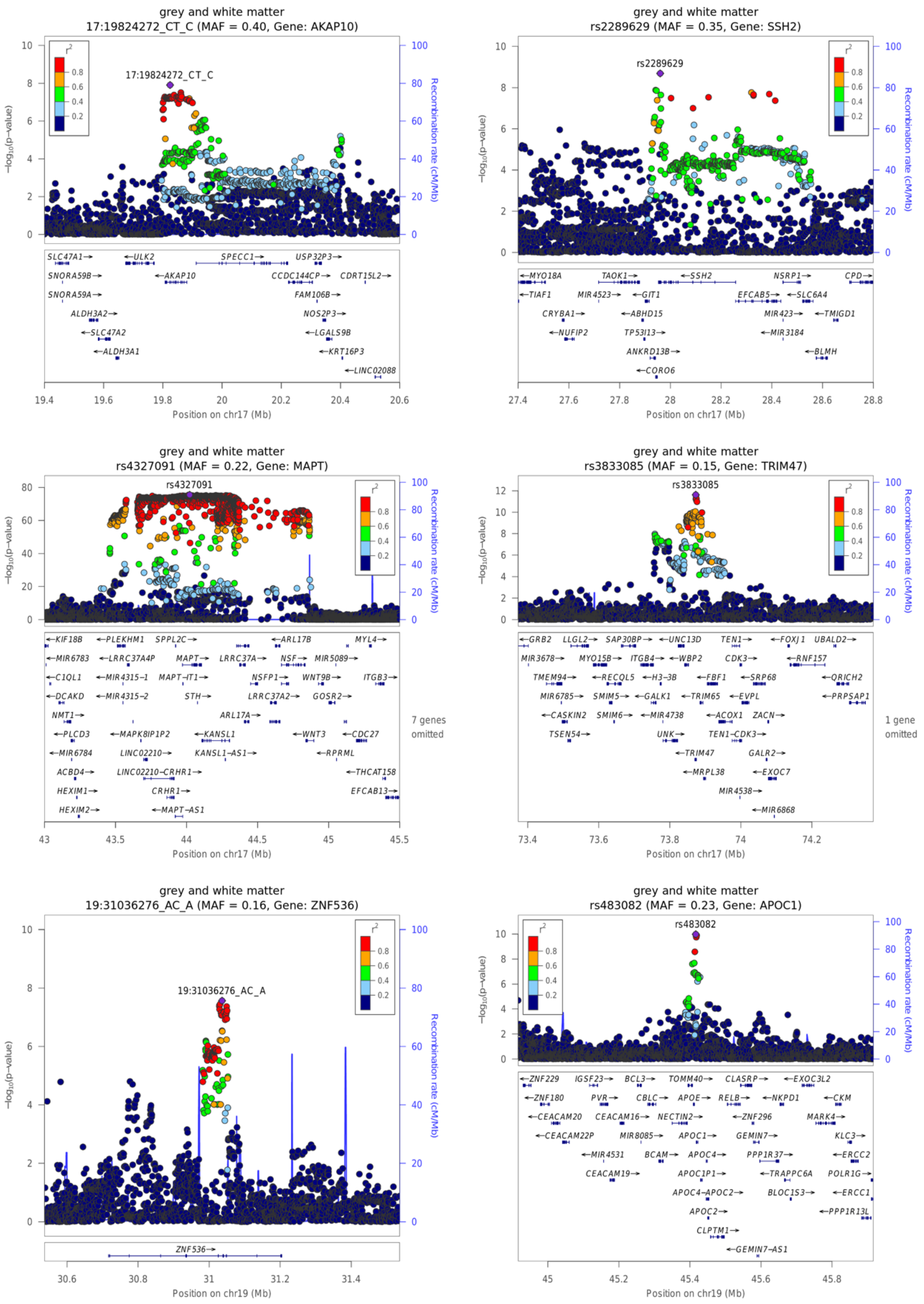

Fig. A41 Regional association plots for index variations 31-36 from the genome-wide association meta-analysis of combined grey and white matter brain age gap in up to $n = 54{,}890$ European ancestry individuals. Regional association plots were created using Locuszoom Standalone (v1.4). SNP positions (dbSNP build 151) and refFlat gene locations (2020-08-17) are based on human genome build hg19 and were accessed via UCSC Genome Browser. Recombination rates were derived from HapMap phase II build GRCh37 (2011-01-19). MAF: Minor allele frequency, r^2: linkage disequilibrium between index variation and other variation in locus

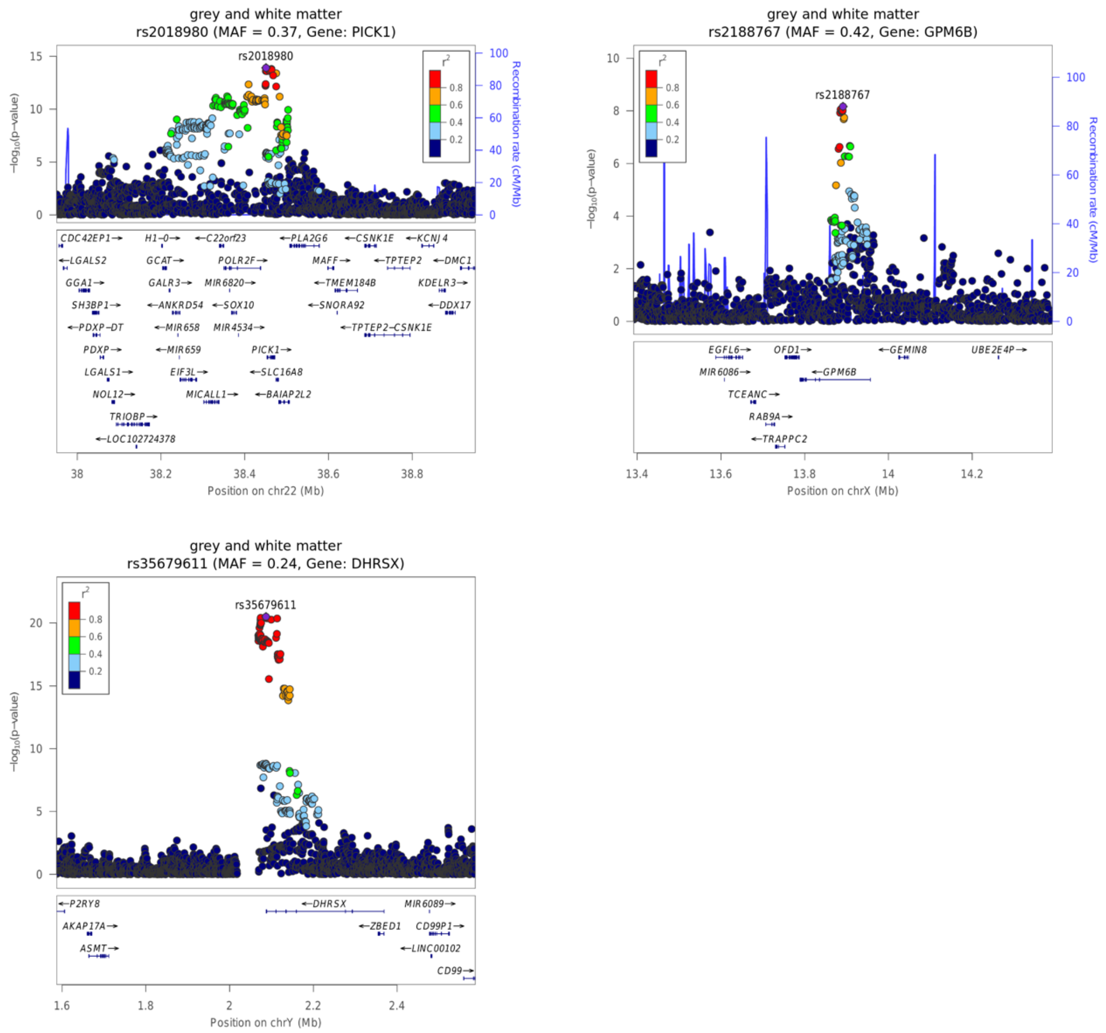

Fig. A42 Regional association plots for index variations 37-39 from the genome-wide association meta-analysis of combined grey and white matter brain age gap in up to $n = 54{,}890$ European ancestry individuals. Regional association plots were created using Locuszoom Standalone (v1.4). SNP positions (dbSNP build 151) and refFlat gene locations (2020-08-17) are based on human genome build hg19 and were accessed via UCSC Genome Browser. Recombination rates were derived from HapMap phase II build GRCh37 (2011-01-19). MAF: Minor allele frequency, r^2: linkage disequilibrium between index variation and other variation in locus

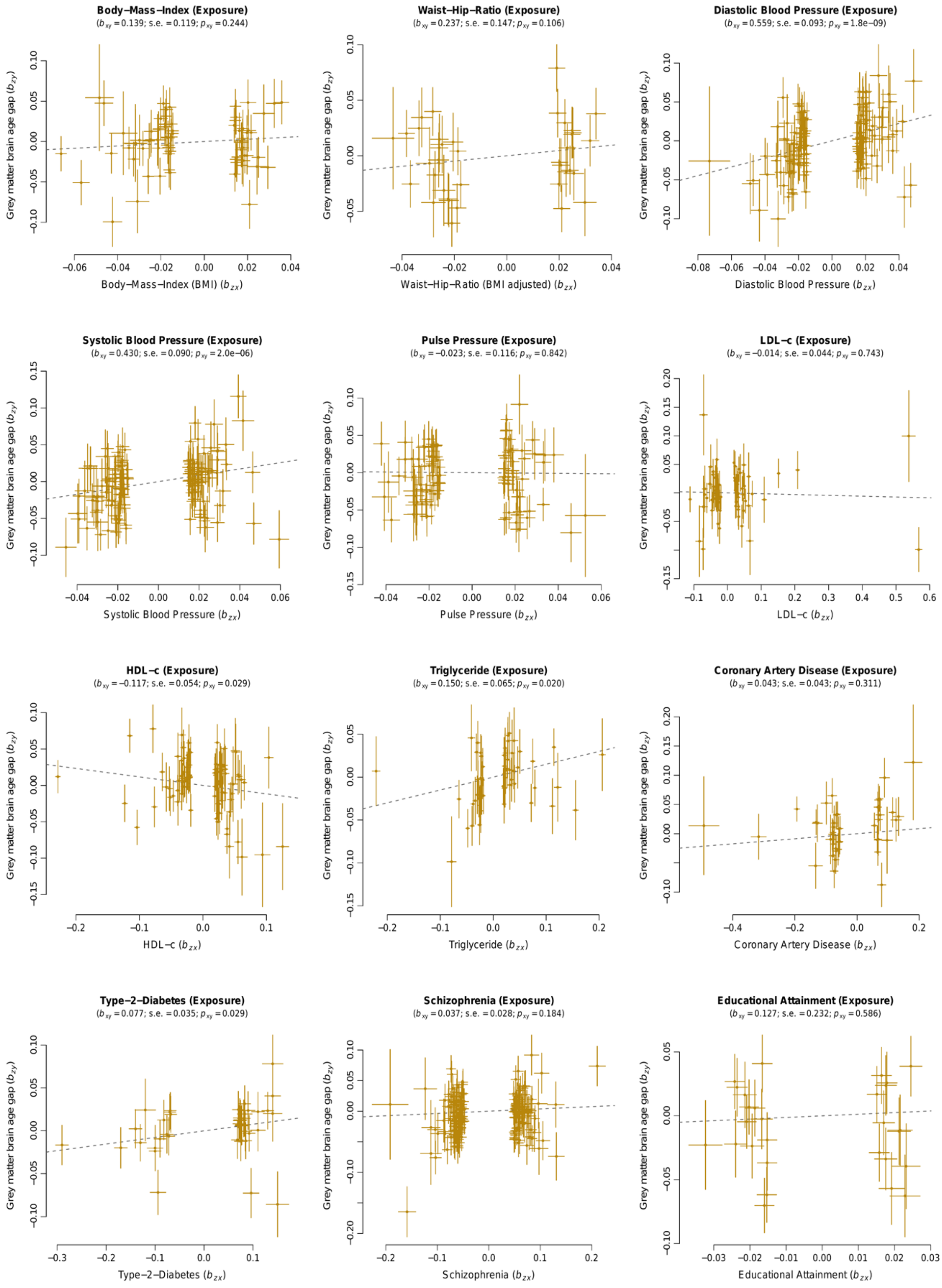

Fig. A43 Results of the generalized summary-data-based Mendelian Randomization (GSMR) analyses for 12 modifiable risk factors (exposure) and grey matter brain age gap (outcome). Each plot shows multiple genetic variants serving as instruments to test for causality between the exposure and outcome variable. Under a causal model, variant effects on the outcome (b_{zy}; y-axis) are expected to be linearly proportional to the variant effects on the exposure variable (b_{zx}; x-axis). The ratio between b_{zy} and b_{zx} provides an estimate of the mediation effect of x on y (b_{xy}). Variants with potential horizontal pleiotropic effects were identified using the HEIDI-outlier method and were removed in advance. s.e. standard error of the mediation effect; p_{xy} p-value of the mediation effect

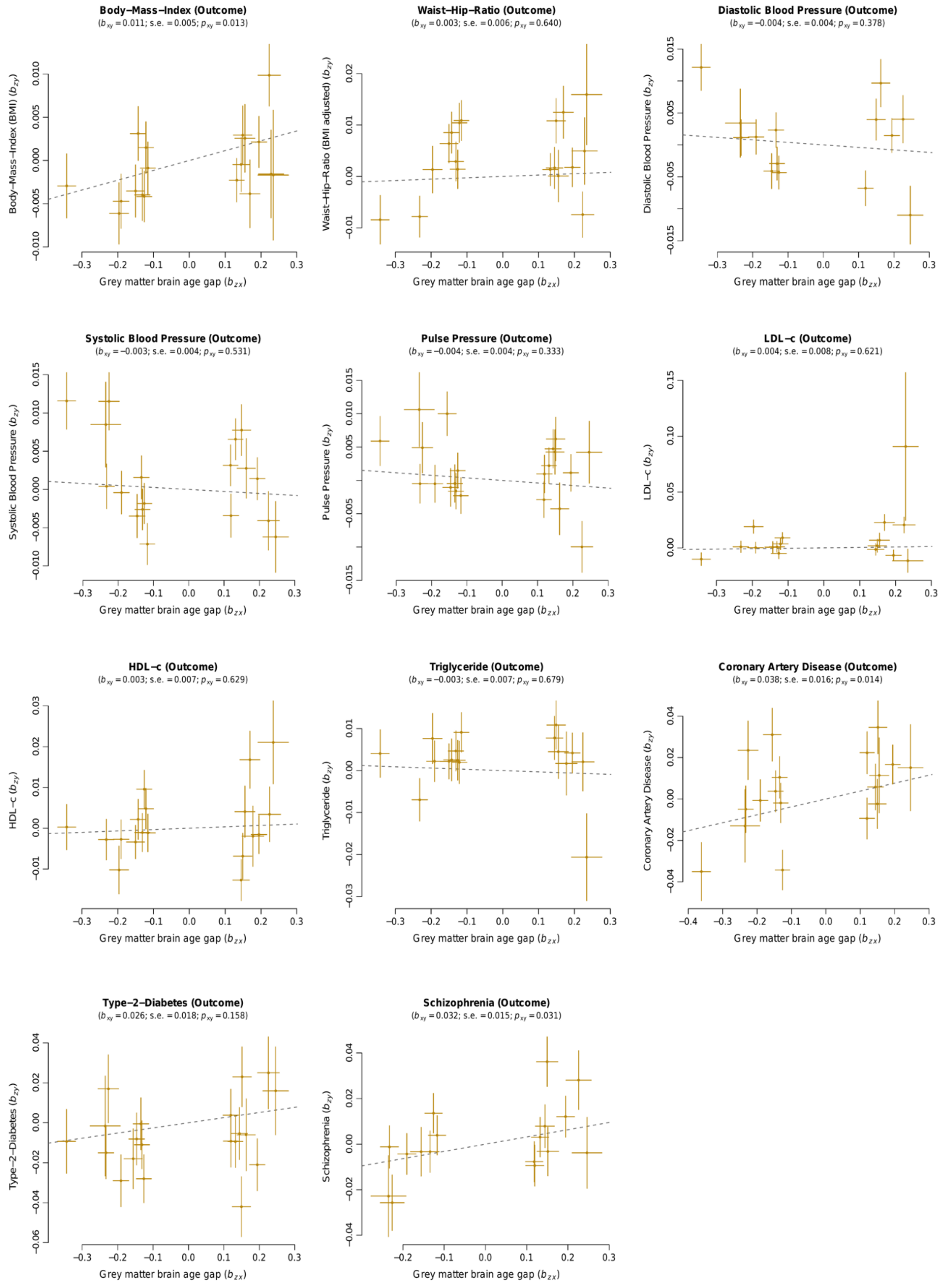

Fig. A44 Results of the reversed generalized summary-data-based Mendelian Randomization (GSMR) analyses for grey matter brain age gap (exposure) and 11 risk factors (outcome). Each plot shows multiple genetic variants serving as instruments to test for causality between the exposure and outcome variable. Under a causal model, variant effects on the outcome (b_{zy}; y-axis) are expected to be linearly proportional to the variant effects on the exposure variable (b_{zx}; x-axis). The ratio between b_{zy} and b_{zx} provides an estimate of the mediation effect of x on y (b_{xy}). Variants with potential horizontal pleiotropic effects were identified using the HEIDI-outlier method and were removed in advance. s.e. standard error of the mediation effect; p_{xy} p-value of the mediation effect

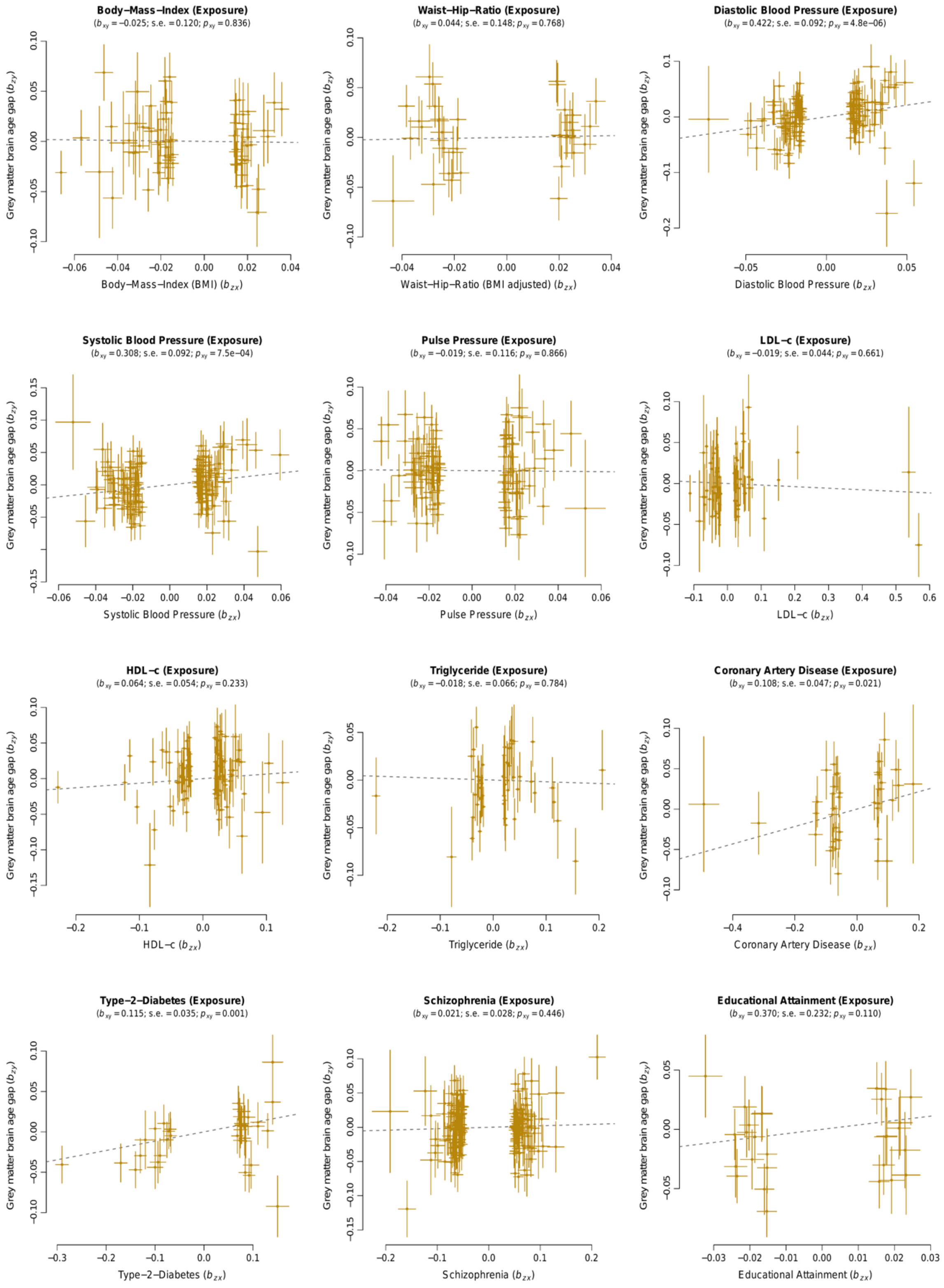

Fig. A45 Results of the generalized summary-data-based Mendelian Randomization (GSMR) analyses for 12 modifiable risk factors (exposure) and white matter brain age gap (outcome). Each plot shows multiple genetic variants serving as instruments to test for causality between the exposure and outcome variable. Under a causal model, variant effects on the outcome (b_{zy}; y-axis) are expected to be linearly proportional to the variant effects on the exposure variable (b_{zx}; x-axis). The ratio between b_{zy} and b_{zx} provides an estimate of the mediation effect of x on y (b_{xy}). Variants with potential horizontal pleiotropic effects were identified using the HEIDI-outlier method and were removed in advance. s.e. standard error of the mediation effect; p_{xy} p-value of the mediation effect

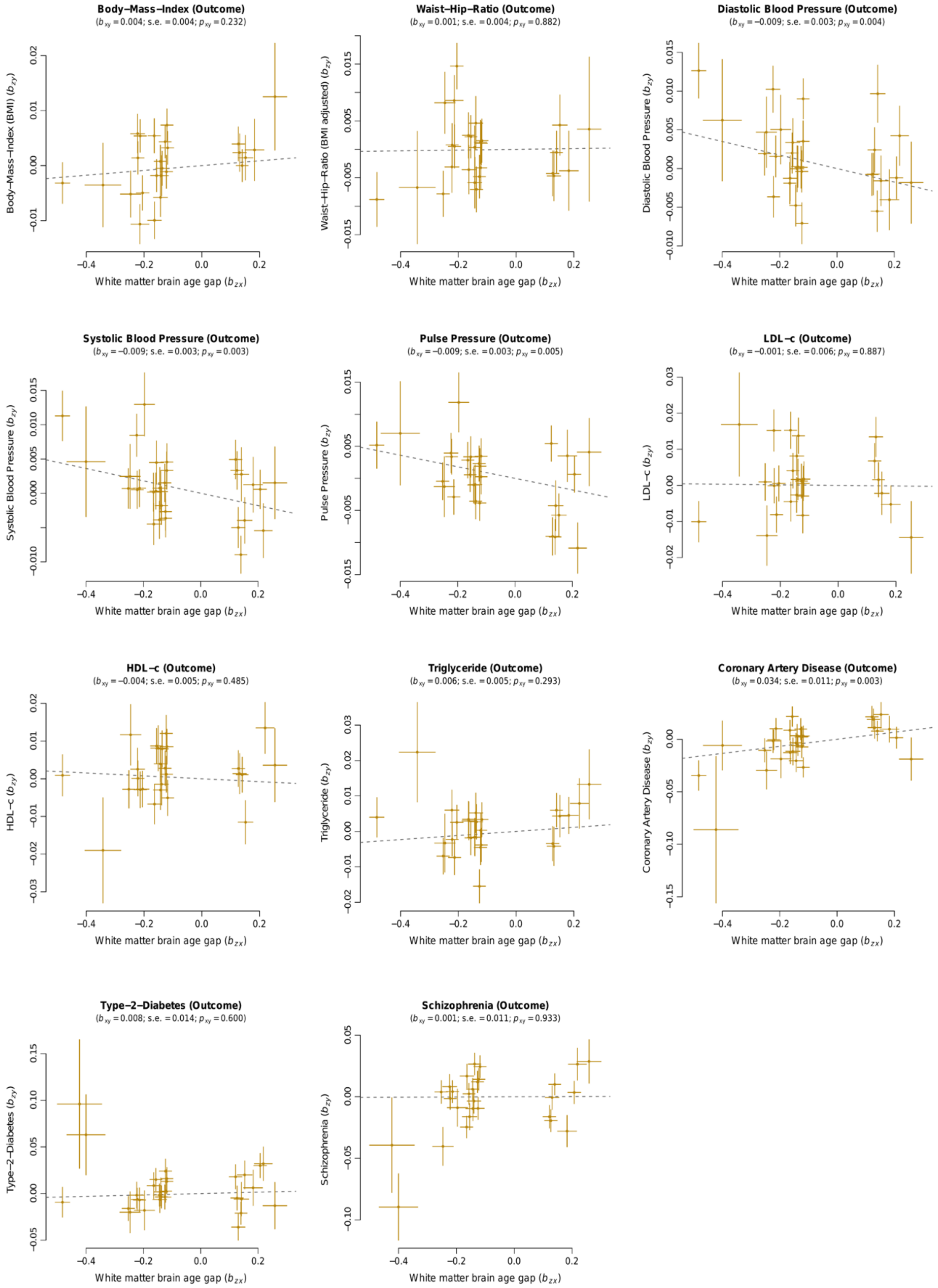

Fig. A46 Results of the reversed generalized summary-data-based Mendelian Randomization (GSMR) analyses for white matter brain age gap (exposure) and 11 risk factors (outcome). Each plot shows multiple genetic variants serving as instruments to test for causality between the exposure and outcome variable. Under a causal model, variant effects on the outcome (b_{zy}; y-axis) are expected to be linearly proportional to the variant effects on the exposure variable (b_{zx}; x-axis). The ratio between b_{zy} and b_{zx} provides an estimate of the mediation effect of x on y (b_{xy}). Variants with potential horizontal pleiotropic effects were identified using the HEIDI-outlier method and were removed in advance. s.e. standard error of the mediation effect; p_{xy} p-value of the mediation effect

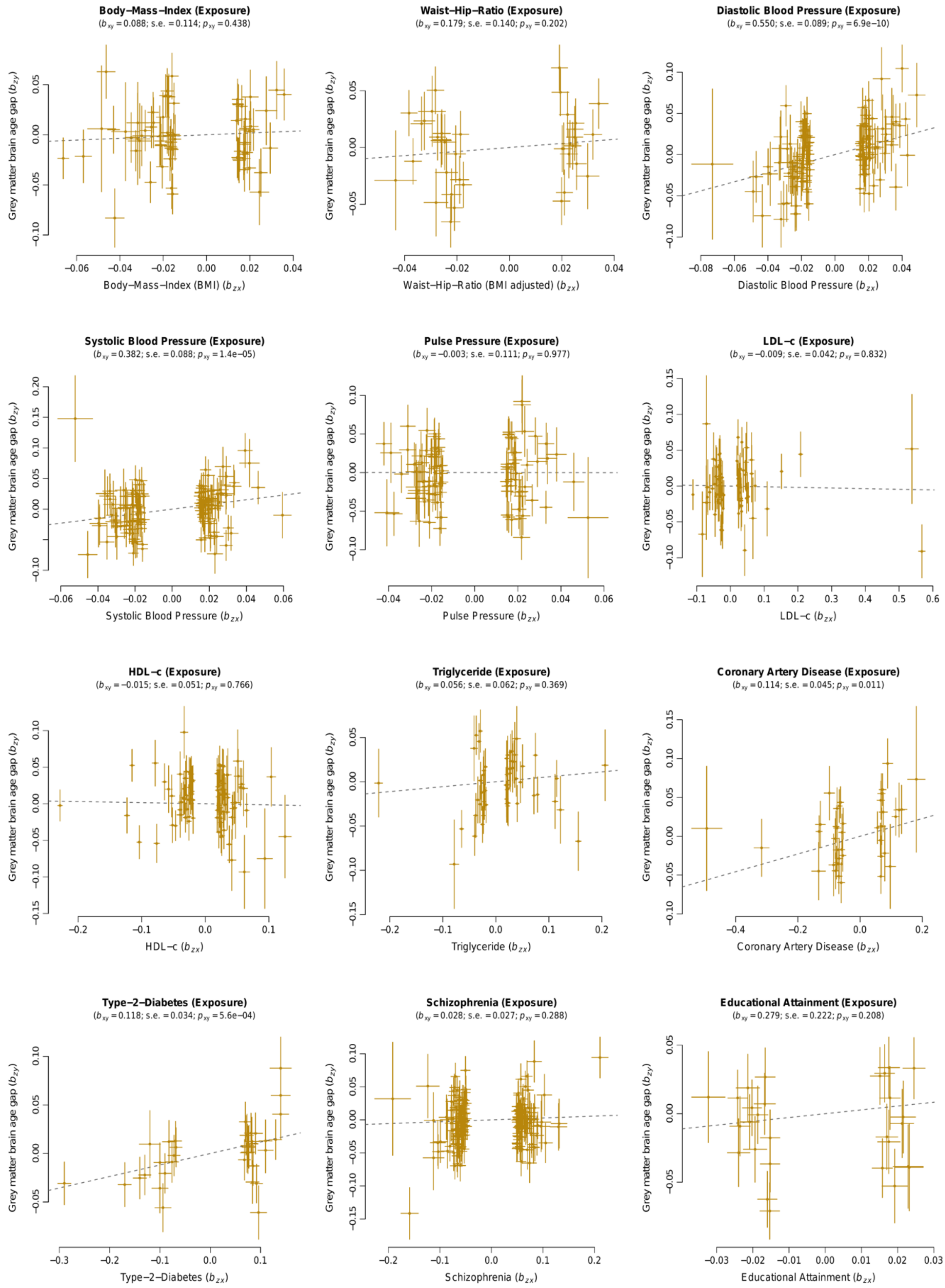

Fig. A47 Results of the generalized summary-data-based Mendelian Randomization (GSMR) analyses for 12 modifiable risk factors (exposure) and combined grey and white matter brain age gap (outcome). Each plot shows multiple genetic variants serving as instruments to test for causality between the exposure and outcome variable. Under a causal model, variant effects on the outcome (b_{zy}; y-axis) are expected to be linearly proportional to the variant effects on the exposure variable (b_{zx}; x-axis). The ratio between b_{zy} and b_{zx} provides an estimate of the mediation effect of x on y (b_{xy}). Variants with potential horizontal pleiotropic effects were identified using the HEIDI-outlier method and were removed in advance. s.e. standard error of the mediation effect; p_{xy} p-value of the mediation effect

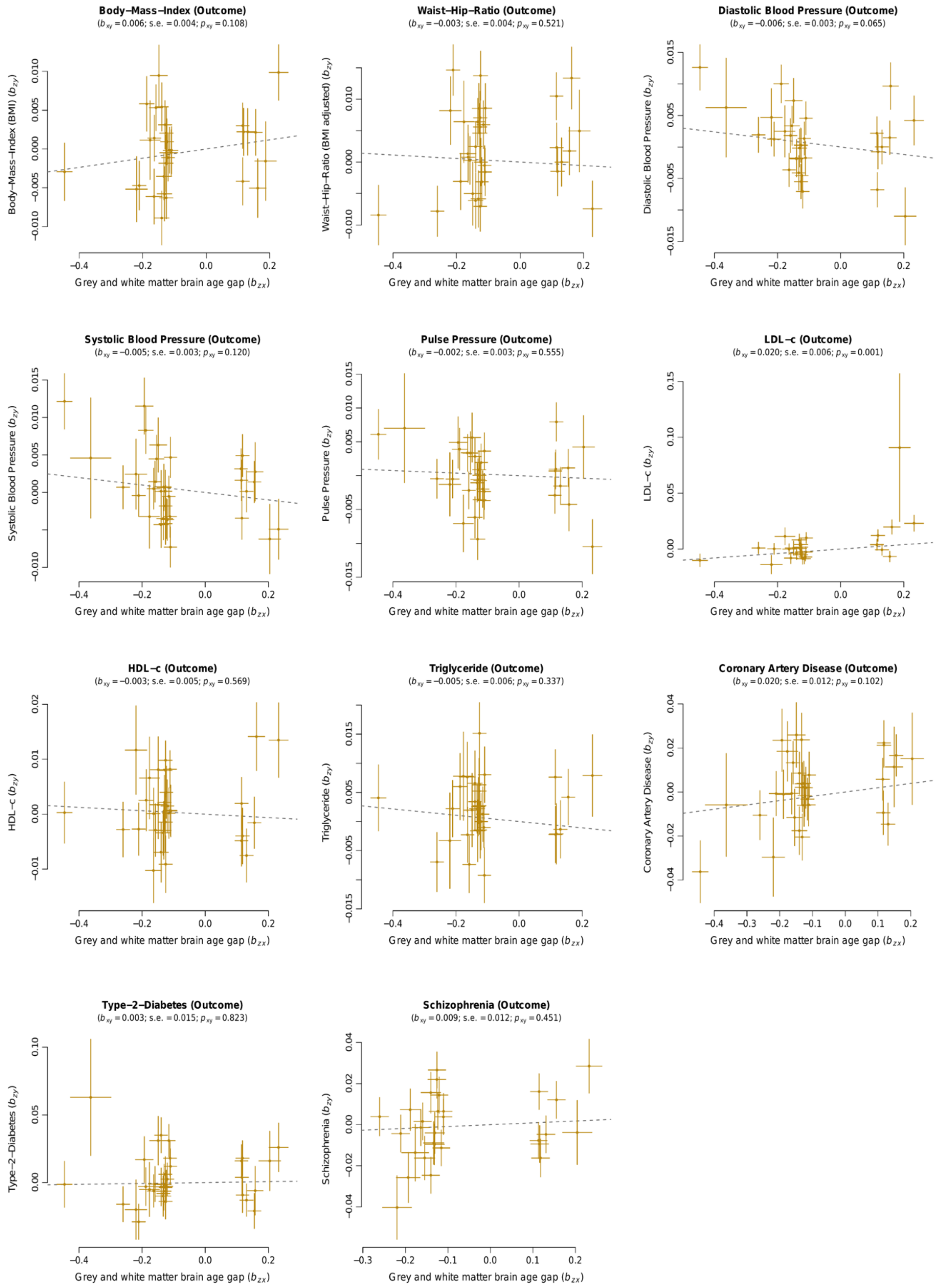

Fig. A48 Results of the reversed generalized summary-data-based Mendelian Randomization (GSMR) analyses for combined grey and white matter brain age gap (exposure) and 11 risk factors (outcome). Each plot shows multiple genetic variants serving as instruments to test for causality between the exposure and outcome variable. Under a causal model, variant effects on the outcome (b_{zy}; y-axis) are expected to be linearly proportional to the variant effects on the exposure variable (b_{zx}; x-axis). The ratio between b_{zy} and b_{zx} provides an estimate of the mediation effect of x on y (b_{xy}). Variants with potential horizontal pleiotropic effects were identified using the HEIDI-outlier method and were removed in advance. s.e. standard error of the mediation effect; p_{xy} p-value of the mediation effect
