# Supplementary material for: Genome-wide analysis of brain age identifies 59 associated loci and unveils relationships with mental and physical health
Source: Nat Aging. 2025 Oct 3;5(10):2086–103. doi: 10.1038/s43587-025-00962-7 (PMC12532595; doi:10.1038/s43587-025-00962-7)
Supplement: Supplementary file 2 — Reporting Summary [file 43587_2025_962_MOESM2_ESM.pdf]

nature portfolio

Corresponding author(s): Philippe Jawinski

Last updated by author(s): Jul 10, 2025

# Reporting Summary

Nature Portfolio wishes to improve the reproducibility of the work that we publish. This form provides structure for consistency and transparency in reporting. For further information on Nature Portfolio policies, see our Editorial Policies and the Editorial Policy Checklist.

## Statistics

For all statistical analyses, confirm that the following items are present in the figure legend, table legend, main text, or Methods section.

| n/a | Confirmed | |
|---|---|---|
| ☐ | ☒ | The exact sample size (*n*) for each experimental group/condition, given as a discrete number and unit of measurement |
| ☐ | ☒ | A statement on whether measurements were taken from distinct samples or whether the same sample was measured repeatedly |
| ☐ | ☒ | The statistical test(s) used AND whether they are one- or two-sided<br>*Only common tests should be described solely by name; describe more complex techniques in the Methods section.* |
| ☐ | ☒ | A description of all covariates tested |
| ☐ | ☒ | A description of any assumptions or corrections, such as tests of normality and adjustment for multiple comparisons |
| ☐ | ☒ | A full description of the statistical parameters including central tendency (e.g. means) or other basic estimates (e.g. regression coefficient) AND variation (e.g. standard deviation) or associated estimates of uncertainty (e.g. confidence intervals) |
| ☐ | ☒ | For null hypothesis testing, the test statistic (e.g. *F*, *t*, *r*) with confidence intervals, effect sizes, degrees of freedom and *P* value noted<br>*Give P values as exact values whenever suitable.* |
| ☐ | ☒ | For Bayesian analysis, information on the choice of priors and Markov chain Monte Carlo settings |
| ☒ | ☐ | For hierarchical and complex designs, identification of the appropriate level for tests and full reporting of outcomes |
| ☐ | ☒ | Estimates of effect sizes (e.g. Cohen's *d*, Pearson's *r*), indicating how they were calculated |

*Our web collection on statistics for biologists contains articles on many of the points above.*

## Software and code

Policy information about availability of computer code

**Data collection**

Genetic, brain imaging, and phenotypic data were obtained from the UK Biobank and LIFE-Adult cohorts. No software was used by the authors for data collection in the present study.

**Data analysis**

All analysis scripts used in this work are available on GitHub (https://github.com/pjawinski/ukb_brainage), where we also provide conda environments with further details on R and python package versions. Analyses were run on Debian GNU/Linux 11 (bullseye) with kernel version 5.10.0-23-amd64. A list of required tools is provided below.

- R (3.5.1-4.41) | Statistical computing and plotting, included in conda environments
- MATLAB (R2021a) | MRI preprocessing and age-prediction | https://de.mathworks.com/
- SPM12 (r7487) | MRI preprocessing | https://www.fil.ion.ucl.ac.uk/spm/
- CAT12 (r1364) | MRI preprocessing | https://neuro-jena.github.io/cat/
- RVM-Matlab v1.0.0 | Age-prediciton | https://github.com/iqiukp/RVM-MATLAB
- XGBoost v0.82.1 | Age-prediction | https://xgboost.ai/
- PHESANT v1.1| Cross-trait (phenome-wide) association analysis in UK Biobank | https://github.com/MRCIEU/PHESANT
- ENIGMA toolbox v2.0.3 for MATLAB | Visualizing cortical and subcortical surface associations | https://github.com/MICA-MNI/ENIGMA
- PLINK v1.90b6.8 64-bit Intel | Genomic preprocessing | https://www.cog-genomics.org/plink/
- PLINK v2.00a2LM 64-bit Intel | Genomic preprocessing and genome-wide association analysis | https://www.cog-genomics.org/plink/2.0/
- METAL 2020-05-05 | Fixed-effects meta-analysis in European ancestry samples | https://csg.sph.umich.edu/abecasis/Metal/
- MR-MEGA v0.2 | Multi-ancestry meta-analysis (meta-regression) | https://genomics.ut.ee/en/tools
- GWAMA v2.2.2 | Multi-ancestry meta-analysis (random-effects) | https://genomics.ut.ee/en/tools
- LOCUSZOOM v1.4 standalone | Regional association plots | https://genome.sph.umich.edu/wiki/LocusZoom_Standalone

- GCTA v1.93.1f beta Linux | Conditional, gene-based, and MR analyses | https://yanglab.westlake.edu.cn/software/gcta/
- LD Score Regression v1.0.1 | Heritability & stratified heritability analysis; Genetic correlations | https://github.com/bulik/ldsc
- SMR v1.03 | Summary-data-based Mendelian Randomization | https://yanglab.westlake.edu.cn/software/smr/
- LDSTORE v2.0 x86/64 | Estimating and storing linkage-disequilibrium data | http://www.christianbenner.com/
- FINEMAP v1.4.2 | Identification of causal variants | http://www.christianbenner.com/
- susieR v0.12.35 | Identification of causal variants | https://github.com/stephenslab/susieR
- GCTB v2.5.2 | Identification of causal variants and polygenic score analysis
- PRSice2 v2.3.3 | Polygenic score analysis | https://choishingwan.github.io/PRSice/
- MAGMA v1.10 Linux Debian 64-bit | Gene-based analysis to produce input for PoPS | https://ctg.cncr.nl/software/magma
- PoPS v0.2 | Gene prioritization | https://github.com/FinucaneLab/pops
- GOfuncR 1.14.0 | Gene Set Enrichment Analysis | https://github.com/sgrote/GOfuncR
- GENESIS e4e6894 | Polygenicity analysis

For manuscripts utilizing custom algorithms or software that are central to the research but not yet described in published literature, software must be made available to editors and reviewers. We strongly encourage code deposition in a community repository (e.g. GitHub). See the Nature Portfolio guidelines for submitting code & software for further information.

# Data

Policy information about availability of data

All manuscripts must include a data availability statement. This statement should provide the following information, where applicable:

- Accession codes, unique identifiers, or web links for publicly available datasets
- A description of any restrictions on data availability
- For clinical datasets or third party data, please ensure that the statement adheres to our policy

The individual-level data used in this study were obtained from the UK Biobank (https://www.ukbiobank.ac.uk/) and LIFE-Adult study (https://www.uniklinikum-leipzig.de/einrichtungen/life). Access to these datasets is restricted to researchers with approved projects. The GWAS summary statistics and polygenic score weights generated from our analyses are publicly available on Zenodo (https://doi.org/10.5281/zenodo.14826943). Genetic correlation analyses involving UK Biobank traits were conducted using GWAS summary statistics provided by Neale and colleagues (https://doi.org/10.5281/zenodo.7186871). Additional GWAS summary statistics used for genetic correlation and Mendelian Randomization analyses are detailed in Supplementary Tables B28 and B31. All scripts used in this work are available on GitHub (https://github.com/pjawinski/ukb_brainage).

# Research involving human participants, their data, or biological material

Policy information about studies with human participants or human data. See also policy information about sex, gender (identity/presentation), and sexual orientation and race, ethnicity and racism.

| | |
|---|---|
| Reporting on sex and gender | Analyses were restricted to participants whose self-reported sex aligned with their genetic sex. Gender information was not collected. Sex was utilized as a covariate in our GWAS and cross-trait association analyses. Sex-stratified analyses were added during the revision in response to a reviewer's request. The discovery sample comprised 32,634 participants (17,084 female), while the full multi-ancestry replication sample included 23,714 participants (12,339 female). |
| Reporting on race, ethnicity, or other socially relevant groupings | Participants were assigned to ancestry groups to account for population stratification in GWAS analyses. In our discovery GWAS, only individuals of white-British ancestry were included (UKB data-field 22006). In replication analyses, individuals with a valid ancestry assignment from the Pan-ancestry UKB project were included (UKB return 2442; https://pan.ukbb.broadinstitute.org/). We define "ancestry" as a statistical concept based on the genetic variants inherited from ancestors, distinct from the social constructs of "race" and "ethnicity". Throughout our analyses, we specifically refer to genetic ancestry, utilizing the following broad categories:<br><br>- White-British ancestry (for discovery only)<br>- European ancestry<br>- Central/South Asian ancestry<br>- African ancestry<br>- East Asian ancestry<br>- Middle Eastern ancestry<br>- Admixed American ancestry<br><br>Ancestry assignment was conducted by comparing each participant's genome in the UK Biobank with data from two extensive reference datasets, the 1000 Genomes Project and the Human Genome Diversity Project (HGDP). These ancestry labels are used solely for GWAS purposes and may not align with other demographic or self-reported data. |
| Population characteristics | The discovery sample included 32,634 UKB participants of white-British ancestry (17,084 female, age range: 45.2-81.9 years, mean age: 64.3 years). The replication sample included 23,714 participants (12,339 female, age range: 45.2-81.9 years, mean age: 67.0 years) from 7 subsamples: 337 African, 94 Admixed American, 638 Central/South Asian, 219 East Asian, 20,423 European, and 98 Middle Eastern ancestry UK Biobank participants (11,451 female, age range: 45.5-81.9 years, mean age: 67.1 years), along with 1,833 European ancestry participants of the LIFE-Adult cohort (888 female, age range: 45.2-80.3 years, mean age: 65.3 years). |
| Recruitment | The UK Biobank recruited 500,000 volunteers aged 40 to 69 from various regions across the UK between 2006 and 2010. Assessments were conducted in 22 centers spread across Scotland, England, and Wales. Participants were identified through NHS patient registers if they fell within the specified age range and lived reasonably close to an assessment center. Out of nine million invited individuals, 5.5% (500,000) ultimately participated in the study. Approximately 50,000 individuals underwent brain MRI scans at one of four UK Biobank imaging centers located in Cheadle, Newcastle, Reading, and Bristol. |

Previous studies have shown that volunteers from the UK Biobank tend to have healthier lifestyles, higher educational attainment, and better overall health compared to the general UK population [doi: 10.1097/EDE.0000000000001316; 10.1192/bjo.2019.100].

The LIFE-Adult study is a population-based study of 10,000 randomly selected adult citizens of Leipzig. Recruitment and examination were conducted from August 2011 until November 2014. Randomly sampled citizens' address lists were obtained from the resident registration office of Leipzig, Germany. Among 29,535 citizens aged 40–79 invited to participate, 31.0% accepted, 29.0% declined, and 36.3% did not respond [doi: 10.1093/ije/dyac114]. The remaining 3.7% either could not be contacted or declined despite expressing initial interest. Analysis comparing the study participants to both the general Leipzig population and non-participants, utilizing official statistics and brief questionnaire data, revealed that study participants were less frequently elderly women and more commonly married, highly educated, employed, in better health, and more frequently current non-smokers [doi: 10.1186/s12874-019-0779-8]

GWAS and post-GWAS analyses have previously been shown to be sensitive to participation bias [doi: 10.1038/s41562-023-01579-9]. Although large volunteer-based biobanks are crucial for advancing genetic epidemiology, the presence of participation bias introduces the possibility of altered results (e.g., altered heritability estimates, genetic correlations, and Mendelian Randomization estimates). Notably, previous studies have observed such alterations more prominently in socio-behavioral traits rather than physical ones. Additionally, research suggests that increasing the representativeness of study samples may affect the magnitude of effects rather than their direction. Given these considerations, we maintain that our inferences and conclusions regarding brain age gap are less susceptible to the influence of participation bias.

Ethics oversight

The UK Biobank received ethical approval from the National Research Ethics Service Committee North West-Haydock (reference 11/NW/0382, 16/NW/0274, 21/NW/0157). The LIFE-Adult study was approved by the Ethics Committee of the University of Leipzig (263–2009-14122009, 263/09-ff, 201/17-ek). All participants provided written informed consent. LIFE-Adult participants received a fixed compensation of 20 EUR per visit to cover travel expenses, while UK Biobank participants could claim reimbursement for travel costs.

Note that full information on the approval of the study protocol must also be provided in the manuscript.

# Field-specific reporting

Please select the one below that is the best fit for your research. If you are not sure, read the appropriate sections before making your selection.

☒ Life sciences ☐ Behavioural & social sciences ☐ Ecological, evolutionary & environmental sciences

For a reference copy of the document with all sections, see nature.com/documents/nr-reporting-summary-flat.pdf

# Life sciences study design

All studies must disclose on these points even when the disclosure is negative.

Sample size

The majority of participants were drawn from the January 2020 release (v1.7) of the UK Biobank imaging study (www.ukbiobank.ac.uk). This release contained 40,681 participants with structural T1-weighted MRI data. After applying all data exclusions (see below), the final GWAS discovery sample included 32,634 individuals. For replication analyses, we selected all remaining non-white-British ancestry individuals of the January 2020 release, and added European and non-European ancestry participants whose imaging data were released until May 2024 (v1.10), along with individuals of the LIFE-Adult cohort, yielding a total replication sample of 23,714 participants. The success of a GWAS for a specific trait mainly depends on factors such as the strength of heritability, the number of contributing variants and the distribution of their effect sizes (i.e., the degree of polygenicity), and the achieved sample size. Brain age gap has previously been shown with a relatively strong SNP-based heritability of 20%. Additionally, previous GWAS with smaller sample sizes have been successful in identifying some initial genome-wide significant loci for brain age gap, although the trait is generally considered to have a high degree of polygenicity. Given that our study boasts a 79% increase in sample size (about 25,000 more) compared to the most recent GWAS on brain age gap (Wen et al. 2024, Yi et al. 2025), we believe that our sample size is adequate.

Data exclusions

We did not include T1-weighted MRI scans labelled as 'unusable' and those that did not successfully complete the CAT12 voxel-based morphometry preprocessing. For further downstream analyses, only MRI scans with a CAT12 overall image quality rating < 3.0 were considered. Analyses were limited to participants whose self-reported sex matched the genetic sex (UKB data-field 31 and 2200), who were without indications of sex aneuploidy (data-field 22019), and who were no outliers in heterozygosity and missingness (data-field 22027). We only included unrelated participants as suggested by pairwise kinship coefficients below 0.0442 (pre-calculated coefficients retrieved using the command line tool 'ukbgene' with the 'rel' parameter). In the discovery GWAS, only participants of white-British ancestry were included (data-field 22006). In replication analyses, all remaining individuals of non-white-British ancestry were included, along with individuals from UKB release v1.10 (meeting the same quality criteria). Furthermore, in replication analyses, only individuals with a valid ancestry assignment from the Pan-ancestry UKB project were included (UKB return 2442; https://pan.ukbb.broadinstitute.org/). From the LIFE-Adult cohort, we included only individuals within the same age range as UK Biobank participants, and who had available T1-weighted MRI data and passed the study's genetic quality control procedures, including checks for cryptic relatedness and consistency between reported and genetic sex.

Replication

Independent associations from the discovery GWAS of 32,634 individuals were tested for replication in both a European ancestry and multi-ancestry meta-analysis of up to 23,714 individuals. Replication analyses included index variations from the 25 genome-wide significant loci, and index variations from another 45 suggestive loci (conditional p-values ranging from 1.0e-06 to 5.0e 08). The degree of consistency between discovery and replication results was highly unlikely to occur by chance. Of the 25 discoveries, all showed consistent effect directions (binomial test: $p = 3.0e-08$) and 18 replicated at $p < 0.05$ (one-tailed nominal significance; binomial test: $p = 1.3e-18$). This finding aligns closely with the outcomes predicted by statistical power analyses, with 19 out of 25 loci expected to attain one-tailed nominal significance in replication analyses.

Randomization

This study was observational and non-experimental; thus, participants were not randomly assigned, and no formal blinding was applied.

| | |
|---|---|
| Randomization | Allocation of participants to genotype groups can be regarded as a result of random transmission of alleles from parents to offspring (caveat: assortative mating may be a potential source of bias). Several covariates were used in cross-trait analyses: sex, age, age2, scanner site, and total intracranial volume. The following covariates were used in GWAS analyses: sex, age, age2, scanner site, total intracranial volume, genotyping array, and up to 20 genetic principal components. |
| Blinding | This study was observational and non-experimental; thus, participants were not randomly assigned, and no formal blinding was applied. However, we assume that investigators involved in data collection were unaware of genotype information. Given the large number of variants analyzed (~9.6 million), and the hypothesis-free nature of GWAS, we do not consider blinding to be a relevant source of bias in data collection or analysis. |

# Reporting for specific materials, systems and methods

We require information from authors about some types of materials, experimental systems and methods used in many studies. Here, indicate whether each material, system or method listed is relevant to your study. If you are not sure if a list item applies to your research, read the appropriate section before selecting a response.

## Materials & experimental systems

| n/a | Involved in the study |
|---|---|
| ☒ | ☐ Antibodies |
| ☒ | ☐ Eukaryotic cell lines |
| ☒ | ☐ Palaeontology and archaeology |
| ☒ | ☐ Animals and other organisms |
| ☒ | ☐ Clinical data |
| ☒ | ☐ Dual use research of concern |
| ☒ | ☐ Plants |

## Methods

| n/a | Involved in the study |
|---|---|
| ☒ | ☐ ChIP-seq |
| ☒ | ☐ Flow cytometry |
| ☐ | ☒ MRI-based neuroimaging |

# Plants

| | |
|---|---|
| Seed stocks | No plants were used in this study. |
| Novel plant genotypes | No plants were used in this study. |
| Authentication | No plants were used in this study. |

# Magnetic resonance imaging

## Experimental design

| | |
|---|---|
| Design type | Only structural T1-weighted MRI data were used in this study. |
| Design specifications | We did not perform an MRI experiment involving varying task or stimulus conditions. However, to estimate the test-retest reliability of brain age estimates in the UK Biobank, we used structural MRI scans from the initial imaging visit (2014+) and the first repeat imaging visit (2019+). Only scans from the initial imaging visit were included in the genetic analyses. |
| Behavioral performance measures | No behavioral measures relevant to the present study were collected during the MRI sessions. |

## Acquisition

Imaging type(s) | structural

Field strength | 3T

Sequence & imaging parameters | In the UK Biobank cohort, T1-weighted structural MRI scans were acquired using a 3D MPRAGE sequence in the sagittal plane, with 1×1×1 mm voxel-size, 208×256×256 acquisition matrix, 2,000 ms repetition time (TR), 2.01 ms echo time (TE), 880 ms inversion time (TI), 6.1 ms echo spacing, 8° flip angle, 240 Hz/pixel bandwidth, in-plane acceleration factor of R = 2, and 4:54 min duration (https://www.fmrib.ox.ac.uk/ukbiobank/protocol/). In the LIFE-Adult study, T1-weighted structural images were obtained using a 3D MPRAGE sequence with 1×1×1 mm voxel-size, 256×240×176 acquisition matrix, TR = 2,300 ms, TE = 2.98 ms, TI = 900 ms, and 9° flip angle.

Area of acquisition | whole-brain

Diffusion MRI ☐ Used ☒ Not used

## Preprocessing

Preprocessing software | T1-weighted MRI scans in NIFTI-format were preprocessed using the voxel-based morphometry pipeline of CAT12 (r1364, http://dbm.neuro.uni-jena.de) for SPM12 (r7487) in MATLAB R2021a (The MathWorks Inc, Natick, MA, USA). CAT12 preprocessing involved affine and DARTEL registration of brain images to a reference brain, segmentation into grey matter, white matter, and cerebro-spinal fluid, bias correction for intensity inhomogeneity, and modulation of segmentations to account for the amount of volume changes due to spatial registration. Processed images were smoothed by applying an 8×8×8mm full-width-at-half-maximum (FWHM) gaussian kernel with subsequent resampling to 8mm3 voxel size. We only considered MRI scans with a CAT12 overall image quality rating < 3.0 for further downstream analyses.

Normalization | CAT12 employs DARTEL and the Geodesic Shooting normalization by means of existing templates in MNI space.

Normalization template | Predefined normalization templates were derived from 555 IXI individuals. DARTEL template: Template_1_IXI555_MNI152.nii; Geodesic Shooting template: Template_0_IXI555_MNI152_GS.nii

Noise and artifact removal | CAT12 incorporates a spatial adaptive non-local means (SANLM) denoising filtering procedure, followed by a Markov Random Field approach (MRF), bias-correction, local intensity transformation, and the use of an adaptive maximum a posterior techinque to account for intensity inhomogeneities.

Volume censoring | No volume censoring was applied.

## Statistical modeling & inference

Model type and settings | We did not perform conventional first and second-level MRI analyses. Instead, the preprocessed, smoothed, and down-sampled brain images underwent dimension-reduction (principal component analysis; PCA) and subsequently served to train our age-prediction models in a supervised machine learning approach.

Effect(s) tested | We did not perform an MRI experiment with task and stimulus conditions.

Specify type of analysis: ☒ Whole brain ☐ ROI-based ☐ Both

Statistic type for inference | We did not perform conventional MRI analyses, i.e., brain images did not serve as outcome but were used to extract features to predict chronological age. Hence, we did not employ either voxel-wise or cluster-wise methods.

(See Eklund et al. 2016)

Correction | For the different types of analyses in our GWAS (cross-trait associations, SNP-level analyses, gene-based analyses, genetic correlations, Mendelian Randomization analyses), we used both multiple-testing corrections based on the Benjamini-Hochberg FDR approach and Bonferroni corrections. This enabled us to identify both a larger number of very likely associations using a more liberal significance threshold and a smaller number of highly reliable results using a more conservative significance threshold.

## Models & analysis

| n/a | Involved in the study |
|---|---|
| ☒ | ☐ Functional and/or effective connectivity |
| ☒ | ☐ Graph analysis |
| ☐ | ☒ Multivariate modeling or predictive analysis |

Multivariate modeling and predictive analysis | Feature Set
CAT12 preprocessed, smoothed, and down-sampled brain images underwent dimension-reduction (principal component analysis; PCA) and subsequently served to train our age-prediction models in a supervised machine learning approach. We selected the first 500 principal components as features, which explained about 90% of the total variance in brain images and enabled model training in a reasonable period of time with advanced computational resources.

Machine Learning Algorithms
Age estimation models were built using the sparse Bayesian relevance vector machine (RVM) in MATLAB, and the extreme gradient boosting package 'xgboost' v.0.82.1 in R. We used XGBoost with both the decision tree ('gbtree') and linear gradient booster ('gblinear'). The learning rate was set to $\eta = 0.02$ with 5000 training iterations and an early stopping after 50 iterations in the case of no further model improvement. The maximum tree depth was set to 3. Default settings were used for all other training parameters.

Model Training and evaluation metrics
Age estimation models were trained with the brain image PCA scores serving as features and chronological age serving as outcome variable. Model training and application was carried out in a 10-fold cross-prediction manner with 100 repeats. Therefore, we randomly split the discovery sample into ten equal-sized subsets, of which nine subsets served for model training, and the remaining subset, the test sample, served for applying the model. Brain images of the training sample underwent PCA, and transformation parameters were subsequently applied to calculate PCA scores in the test sample. After the first model was trained and tested, the next subset served as test sample, while the other nine subsets were selected as training sample. This strategy was carried on until each subset served exactly once as test sample. The tenfold cross-prediction procedure was repeated 100 times, so that 100 predictions were made for each subject. This procedure was performed for each tissue (grey and white matter) and model type (relevance vector machine, xgboost tree, and xgboost linear), resulting in a total number of 600 brain-predicted age estimates per subject. In a nested 10-fold cross-validation approach, we stacked the estimates from the three different model types in an ensemble estimate, resulting in 100 brain-predicted age estimates for grey matter, white matter, and combined grey and white matter, respectively. Finally, these estimates were averaged, leaving one brain-predicted age estimate for grey matter, white matter, and combined grey and white matter for each subject. In the discovery sample, we observed accurate predictions of chronological age, with mean absolute errors (MAE) reaching MAE = 3.09 years and correlation coefficients attaining $r = .86$. Model performances (without retraining) were similar in the multi-ancestry UKB replication sample (MAE = 3.12 years; $r = .85$), and LIFE-Adult sample (MAE = 3.56 years; $r = .86$).
